# A phase 1 study of 4’-thio-2’-deoxycytidine in patients with solid tumors

**DOI:** 10.1007/s00280-026-04969-8

**Published:** 2026-11-05

**Authors:** Geraldine O’Sullivan Coyne, Shivaani Kummar, Larry Rubinstein, Larry Anderson, Kimberly Hill, Ashley Bruns, Nancy Moore, Katherine V. Ferry-Galow, Brandon Miller, Lihua Wang, Joseph M. Covey, Richard L. Piekarz, Laura Kuhlmann, Sarah B. Miller, Lamin Juwara, Jerry Collins, Robert J. Kinders, Ralph E. Parchment, Alice P. Chen, James H. Doroshow

**Affiliations:** 1 Division of Cancer Treatment and Diagnosis, National Cancer Institute, NIH, 31 Center Drive, Bldg. 31 Room 3A-44, Bethesda, 20892 MD USA; 2 Clinical Pharmacodynamic Biomarkers Program, Applied/Developmental Research Directorate, Frederick National Laboratory for Cancer Research, Frederick, MD USA; 3 Leidos Biomedical Research, Frederick National Laboratory for Cancer Research, Frederick, MD 21702 USA; 4 Clinical Monitoring Research Program, Clinical Research Directorate, Frederick National Laboratory for Cancer Research, Frederick, MD USA; 5 Developmental Therapeutics Program, Division of Cancer Treatment and Diagnosis, National Cancer Institute, Bethesda, MD USA; 6 Center for Cancer Research, National Cancer Institute, Bethesda, MD USA; 7 Knight Cancer Institute, Oregon Health and Science University, Portland, OR USA; 8 START Center for Cancer Research, Northwell Health Cancer Institute, NY, USA

**Keywords:** DNMT1 inhibitors, TdCyd, Cancer epigenetics, Circulating tumor cells, p16

## Abstract

**Purpose:**

The DNA methyltransferase (DNMT) inhibitor 4’-thio-2’-deoxycytidine (TdCyd), a second-generation cytidine analog, demonstrated potent preclinical antitumor activity and favorable pharmacokinetics, which led us to explore its safety, efficacy and anti-tumor activity in patients with solid tumors.

**Methods:**

Adult patients with advanced solid tumors received TdCyd orally on days 1–5 and 8–12 in 21-day cycles. Dose escalation proceeded according to design 4 of the Simon accelerated titration design, changing to a 3 + 3 dose escalation design in the event of dose-limiting toxicities. Blood samples were collected for circulating tumor cell (CTC) evaluation of p16 re-expression as a marker of TdCyd epigenetic modulation, and for pharmacokinetic analysis.

**Results:**

Twenty-seven patients were enrolled. The maximum tolerated dose was not reached. The best response was prolonged stable disease (SD), with 3 patients on treatment for ≥ 6 cycles, and there was no dose-response relationship. An increase in the proportion of p16-expressing cytokeratin-positive (CK+) CTCs was detected in 71% (15 of 21) of patients evaluable for p16 response at every dose level in which patients were assessable for pharmacodynamic response; p16 re-expression in vimentin-positive CTCs was reported in 38% (6 of 16) of evaluable patients. Plasma levels of TdCyd metabolites were largely dose proportional and higher levels were detected in patients with higher incidence and severity of study drug–related adverse events.

**Conclusion:**

Prolonged SD and p16 re-expression at lower TdCyd dose levels suggest that longer exposures to low concentrations is worth exploring in future trials, including studies of TdCyd in therapeutic combinations.

**Supplementary Information:**

The online version contains supplementary material available at https://doi.org/10.1007/s00280-026-04969-8.

## Introduction

Hypermethylation of cytosines at CpG sites in the regulatory sequences of tumor suppressor genes is an epigenetic mechanism of gene inactivation involved in cancer development [1–3]. Methylation patterns at CpG sites are established by DNA methyltransferases (DNMTs), of which the most abundant is the maintenance methyltransferase DNMT1 [4]. FDA-approved DNMT inhibitors, such as the DNA nucleoside analogues 5-azacytidine and 5-aza-2’-deoxycytidine, have been shown to be efficacious in the treatment of myelodysplastic syndromes and hematologic malignancies [5, 6], but have shown limited efficacy in solid tumors [7]. Nucleoside analogs reverse hypermethylation by becoming incorporated into DNA in place of cytosine where they engage the active site of DNMT1 and trap it in a covalent complex with the DNA; activation of proteasome-mediated degradation pathways further depletes the free enzyme and inhibits methylation of newly synthetized CpG sites [8, 9]. DNMT1-depleting agents require 2 or more generations of tumor cell growth to reactivate the expression of tumor suppressor genes [9], necessitating prolonged treatment periods, which are confounded by the hematopoietic toxicity associated with this class of drugs [8]. These limitations encouraged the development of alternative second-generation hypomethylating agents with improved therapeutic index, prolonged plasma half-lives and superior efficiency for a wide range of malignancies, including solid tumors.

4’-Thio-2’-deoxycytidine (TdCyd, NSC 764276), a second-generation cytidine analog in which the 4’-O moiety in deoxyribose has been replaced with sulfur, has growth-inhibitory and cytotoxic activity against several cancer cell lines [10] and has been shown to deplete DNMT1 in vitro and in vivo [8, 9]. A good substrate for deoxycytidine kinase, TdCyd is phosphorylated to 4′-thio-cytidine triphosphate (TdCTP) and incorporated into the DNA at rates similar to the natural nucleoside or ara-analog [11]. Specifically, TdCyd gets incorporated into the DNA sequence recognized by the bacterial C5 DNA methyltransferase M*Hha*I which leads to DNA methylation inhibition [12], without inhibition of DNA replication [9]. TdCyd is deaminated to 4’-thio-2’-deoxyuridine (TdUrd), a metabolite that is a poor substrate for thymidine kinase and thymidine phosphorylase, thus limiting TdUrd conversion to 4’-thio-deoxythymidine (TdThd) [9]. Pharmacokinetic studies in nude male mice confirmed rapid plasma clearance of TdCyd (10–15 min) and highlighted the persistence of TdUrd as the primary plasma metabolite (half-life of ~ 45 min) [9]. Activity in several xenografts has been reported in leukemia and lung carcinoma models [9, 13].

Epigenetic silencing of tumor suppressor genes such as p16 and p15—2 important cell cycle regulators during carcinogenesis [14]—by promoter methylation is commonly observed in human epithelial [15] and hematologic malignancies [16]. In vitro experiments have confirmed that 4’-thio-deoxycytidine analogs can reverse silencing of tumor suppressors such as p15 [16] and p16 [8]. More recently, p16 re-expression has been confirmed in circulating tumor cells (CTCs) and tumors of patients with advanced solid tumors receiving the second generation DNMT inhibitor 5-fluoro-2’-deoxycytidine (FdCyd) [17, 18]. Comparative studies indicate that thio-modified analogues retain the epigenetic reprogramming efficacy of classical agents while reducing systemic toxicity, particularly myelotoxicity, which limits the clinical use of FDA-approved DNMT inhibitors, and underpins our interest in exploring these analogues in the clinical setting. Here, we report the results of a phase 1 study exploring the safety, tolerability, and preliminary anti-tumor efficacy of TdCyd in patients with advanced solid tumors.

## Patients and methods

### Eligibility criteria

The study enrolled patients 18 years and older with histologically confirmed solid tumors for which standard therapies had been ineffective or did not exist. Patients were required to have measurable or evaluable disease, a life expectancy of > 3 months, and an ECOG performance status ≤ 2. Adequate organ and marrow function were also required, as defined by absolute neutrophil count ≥ 1,500/µL, platelet count ≥ 100,000/µL, total bilirubin < 1.5× the institutional upper limit of normal (ULN), alanine aminotransferase and/or aspartate aminotransferase ≤ 3× ULN, and creatinine < 1.5× ULN (or creatinine clearance of ≥ 60 mL/min/1.73 m^2^ for patients with levels ≥ 1.5× ULN). Patients were required to have completed any prior therapies ≥ 4 weeks or 5 half-lives (whichever was shorter) prior to enrollment and must have recovered to eligibility levels for performance status and organ function following any prior toxicities. Patients were also required to be willing to provide research blood and urine samples. Patients with known brain metastases or carcinomatous meningitis (with the exception of patients whose brain metastatic disease status had remained stable for ≥ 1 months after treatment of the brain metastases) or with clinically significant illnesses that would compromise participation in the study—including active or uncontrolled infection, immune deficiencies, known HIV infection requiring protease inhibitor therapy, hepatitis B, hepatitis C, uncontrolled diabetes, uncontrolled hypertension, symptomatic congestive heart failure, unstable angina pectoris, myocardial infarction within the past 6 months, uncontrolled cardiac arrhythmia, or psychiatric illness/social situations that would limit compliance with study requirements—were excluded from this study. Patients with malabsorption syndrome or other conditions that would interfere with intestinal absorption were also excluded.

### Trial design

TdCyd was supplied by the National Cancer Institute’s (NCI) Division of Cancer Treatment and Diagnosis [19]. Patients were administered TdCyd orally on days 1–5 and 8–12 in 21-day cycles; dose escalation (Supplementary Table S1) proceeded according to design 4 of the Simon accelerated titration designs [20], changing to a traditional 3 + 3 dose escalation design in the event of 1 dose limiting toxicity (DLT); intrapatient dose escalation was permitted. In January 2017, after 2 recorded *Pneumocystis jirovecii* pneumonia (PJP) cases on trial in non-HIV–infected patients, dose levels were amended (Supplementary Table S1) and PJP prophylaxis was added for all patients, commencing on cycle 1 (or current cycle or treatment for participants already enrolled) and continued throughout treatment. Accepted prophylaxis treatment included trimethoprim-sulfamethoxazole double-strength 1 tablet 3x/week, inhaled pentamidine, or other appropriate prophylaxis selected based on feasibility and patient tolerability (we will refer to all prophylactic treatment as PJP/antifungal prophylaxis for the remainder of the manuscript). Antifungal prophylaxis was removed in November 2018 following several recorded events of prophylaxis-related adverse events (AEs).

The primary objective was to establish the safety, tolerability, and maximum tolerated dose (MTD) of oral TdCyd in patients with refractory solid tumors under the existing treatment schedule. Secondary objectives included: determining preliminary evidence of TdCyd activity; determining the effect of TdCyd on re-expression of select genes silenced by methylation in CTCs; and determining the pharmacokinetics of oral TdCyd. Additional exploratory PK and PD evaluations were conducted.

Patients maintained a study diary to note side effects. The NCI Common Terminology Criteria for Adverse Events version 4.0 was used to grade AEs until April 1, 2018, at which time CTCAE version 5.0 was used. Tumor response was assessed by radiography at baseline and every 2 cycles thereafter and evaluated per RECIST version 1.1 [21].

The study (NCT02423057) was conducted at the National Institutes of Health (NIH) Clinical Center (Bethesda, Maryland, USA) in accordance with the principles of the Declaration of Helsinki and Good Clinical Practice guidelines and was sponsored by the NCI Division of Cancer Treatment and Diagnosis’ (DCTD) Cancer Therapy Evaluation Program (CTEP). The protocol was approved by the National Cancer Institute’s Institutional Review Board (IRB) (https://www.hhs.gov/ohrp/index.html*).* Informed consent was obtained from all participants. The latest protocol version (November 16, 2023) is available as a supplementary appendix.

### Dose-limiting toxicity definitions and safety assessments

DLTs were defined as toxicities that were at least possibly related to study drug within the first cycle of treatment and met one of the following criteria: grade ≥ 3 non-hematological toxicity (including grade 3 nausea, vomiting, or diarrhea only if refractory to standard supportive care; grade 3 electrolyte toxicities only if unable to be corrected to ≤ grade 2 within 24 h; and rise in creatinine levels to Grade 3 if not corrected to Grade 1 or less within 24 h with IV fluids); grade 4 thrombocytopenia (or grade 3 if accompanied by bleeding); grade 4 neutropenia lasting ≥ 5 days or febrile neutropenia; grade 4 lymphopenia ≥ 14 days. In response to a DLT, TdCyd was held until any toxicity resolved to ≤ grade 2 and resumed at the next-lowest dose level. All patients who received at least one dose of study drug were evaluable for safety and toxicity. The protocol was amended in January 2019 to extend the DLT assessment period to include cycle 2 of therapy. However, no patients were enrolled after the implementation of this amendment, and accrual was put on hold pending additional toxicity and efficacy studies in canine clinical trials; the phase 1 clinical study was subsequently closed early for administrative reasons.

### CellSearch^®^ analysis of CTC p16 expression in patient blood specimens

Blood specimens for CTC analysis (7.5 mL) were collected into one 10-mL CellSave preservative tube (Menarini Silicon Biosystems) prior to TdCyd administration on day 1 and then 2–4 h post dose on day 1, on day 8 at any time, and on cycle 2 day 1 at any time. Additional samples were collected on day 1 of all subsequent cycles and at disease progression. CTC samples were processed and analyzed for p16, cytokeratin (CK), and vimentin (V) as described previously [17, 18]; patients were considered assessable for CK^+^ CTC p16 response, or V^+^ CTC p16 response if the baseline specimen and at least one post-treatment specimen each contained ≥ 6 CTCs of CK^+^ or V^+^ subtype, respectively [17]. CK + and V+ CTCs were assessed independently, and not all patients were evaluable for both CTC subtypes.

### Pharmacokinetics

Blood and urine samples were collected to measure drug concentration and the concentration of 2 known TdCyd metabolites: 4’-thio-2’-deoxythymidine (TdThd; NSC 776949) and 4’-thio-2’-deoxyuridine (TdUrd; NSC 776952) (see Supplementary Figure S1 for chemical formulas) [11].

Blood specimens for pharmacokinetic analysis were collected prior to TdCyd administration, on cycle 1 day 1; 15, 30, and 60 min, and 2, 3, 4, and 24 h after agent administration (before another dose was taken); and on cycle 1 day 15 (optional). Patients who had dose-adjustments due to TdCyd-related toxicity were asked to provide an optional sample prior to TdCyd administration on day 1 and then 1, 2, and 24 h after drug administration (before another dose was taken) during the subsequent cycle. At each time point, blood was collected in two 3-mL heparinized tubes preloaded with 20 µl of a 200 mM solution of zebularine to inhibit cytidine deaminase. Plasma was immediately separated by centrifugation and frozen at -70º C until analysis. Plasma samples and plasma standards (containing 2 mM zebularine) were deproteinized by adding 3 volumes of acetonitrile containing 0.1 µM of each labelled stable thionucleoside and 0.2 µM gemcitabine to 50 µL plasma. The samples were centrifuged at 10,000 x g, for 8 min at room temperature, and 5 µL of the resulting supernatant were used per LC/MS analysis.

Compounds of interest were separated on a 2.1 × 150 mm, 5 μm Gemini C18 column (Phenomenex), at 250 µL/min flow, and eluted using a 2‒95% multistep gradient of methanol (solvent B) in 1.5 g/L ammonium acetate pH9 with NH_4_OH (solvent A). Detection and quantification of the thiopyrimidines was accomplished with a Q-Exactive Plus Mass Spectrometer (Thermo Scientific) operated at a resolution of 70,000. Two injections were made per sample for positive and negative ion analysis, respectively. In positive ion mode, compounds were analyzed as their M^+^H^+^ ions (cytidine nucleosides) with a mass tolerance of 10 ppm; in negative ion mode (uridine and thymidine nucleosides) compounds were analyzed as their M^−^H^−^ ions with a mass tolerance of 10 ppm. Quantitation was achieved by comparing ion peak areas of the compounds to their respective isotope internal standards. Standard curves demonstrated linearity between the following ranges: in plasma, 0.002‒25 µM for TdCyd, 0.002‒25 µM for TdUrd, and 0.001‒25 µM for TdThd; in urine, 0.1‒10 µM for TdCyd, and 0.05‒10 µM for TdUrd and for TdThd. Inter-assay variability in response rates was less than 10%. Area under the curve values were calculated by the trapezoidal rule and half-lives were estimated from the linear portion of the log-linear plasma curves.

A 10-mL urine sample was collected just before TdCyd administration; urine was then collected at every void from 0 to 24 h on cycle 1, day 1. Ten µL of the urine sample (containing zebularine) or urine standard (containing known amounts of TdCyd, TdUrd and TdThd) were mixed with 300 µL of a 1:1 acetonitrile: water mix containing 0.5 µM of each of the stable labelled thionucleoside, then centrifuged at 10,000 x g for 8 min at room temperature; LC/MS analysis was performed on 1.5 µL of each supernatant as described above.

TdCyd, TdUrd and TdThd standards, and their stable isotope analogs 1,3-15N2,2–13 C TdCyd, 1,3-15N2, 2,4,5,6-13C4 TdUrd and 6-D, methyl D3 TdThd, as well as zebularine and gemcitabine were obtained from the NCI chemical repository. All other reagents were obtained from commercial sources. For additional information see also Appendix D in the Protocol.

## Results

### Patient demographics

A total of 27 patients were enrolled on this study from May 2015 to January 2019 (Table 1). The median patient age was 62 years (range: 26–80 years). All patients received prior chemotherapy (median = 3, range:1–12), 24 (88.9%) had undergone surgery, and 11 (40.7%) had prior radiation therapy. The patient distribution was approximately equal between the sexes. The group of participants included 59.3% White, 25.9% Black, and 11.1% Asian subjects (Table 1).

The most frequent malignancy was colon adenocarcinoma (6/27 patients; 22.2%), followed by cholangiocarcinoma (4/27 patients; 14.8%), ovarian cancer (3/27 patients; 11.1%), and hepatocellular carcinoma (2/27 patients; 7.4%). Most patients had an ECOG performance status of 1 at baseline (see Table 1 for distribution).

**Table 1:** Patient Characteristics

Characteristics	Number of patients
**Number of patients**,** total**	27
Female (%)	12 (44.4%)
Male (%)	15 (55.6%)
**Median Age**,** years (range)**	62 (26–80)
**Race**	
Asian (%)	3 (11.1%)
Black (%)	7 (25.9%)
Unknown (%)	1 (3.7%)
White (%)	16 (59.3%)
**ECOG performance status (%)**	
0	8 (29.6%)
1	17 (62.9%)
2	2 (7.4%)
**Diagnosis**	
Colon adenocarcinoma	6
Cholangiocarcinoma	4
Ovarian	3
Hepatocellular carcinoma	2
Adenoid cystic carcinoma	1
Alveolar soft part sarcoma	1
Breast cancer	1
Endometrial carcinosarcoma	1
Endometrial adenocarcinoma	1
Malignant paraganglioma	1
Malignant peripheral nerve sheath tumor	1
Renal cell carcinoma	1
Small bowel adenocarcinoma	1
Spindle cell high-grade sarcoma	1
Undifferentiated pleomorphic sarcoma	1
Urothelial carcinoma	1
**Number of prior therapies**	
Prior chemotherapy, median (range)	3 (1–12)
Prior radiation therapy, median (range)	1 (1–3)
Prior surgery, median (range)	1 (1–5)

### Toxicity

All patients were evaluable for drug toxicity. Nineteen patients (70.3%) experienced grade ≥ 2 AEs possibly related to study drug (Table 2). The most common grade ≥ 2 drug-related AEs were lymphopenia (*n* = 13; 17.8% of total grade ≥ 2 drug-related AEs reported on study), hypophosphatemia (*n* = 7; 9.5%), and fatigue (*n* = 6; 8.2%). The highest incidence of grade ≥ 3 drug-related AEs correlated with the highest (14–20 mg) TdCyd doses (Supplementary Table S2). The most commonly occurring drug-related grade ≥ 3 AEs were lymphopenia (*n* = 9; 12.3% of total drug-related AEs), thrombocytopenia and increased aspartate aminotransferase levels (each *n* = 4; 5.5%). There was one DLT on study: patient 14 (adenoid cystic carcinoma) receiving 6 mg TdCyd experienced grade 3 increase in alanine aminotransferase and aspartate aminotransferase levels on cycle 1.

**Table 2:** **Adverse Events Attributed to 4’-thio-2’-deoxycytidine (TdCyd) **Worst-grade events (≥ grade 2) for each patient that were at least possibly related to TdCyd are shown (*n* = 27 patients)

Adverse event	Grade 2	Grade 3	Grade 4	Grade 5
*Hematological*				
Anemia	4	1		
Leukopenia	1	2		
Lymphopenia	4	9		
Neutropenia	2	1		
Thrombocytopenia		1	3	
*Gastrointestinal*				
Dysgeusia	1			
Diarrhea	1			
Flatulence	1			
Mucositis oral		1		
*Metabolism and nutrition disorders*				
Anorexia	2			
Hypoalbuminemia	1			
Hypokalemia		1		
Hypophosphatemia	6	1		
*Liver function*				
Alanine aminotransferase increased	2	2		
Alkaline phosphatase increased	1			
Aspartate aminotransferase increased	1	4		
*Renal*				
Acute kidney injury		1	1	
Creatinine increase	2	2		
*Respiratory*				
Dyspnea	1			
Hypoxia		1		
Lung infection	1	2		
Pneumonia Pneumocystis jiroveci, Mycobacteroides abscessus	1			
Respiratory failure				1
*Other*				
Fatigue	6			
Maculo-papular rash	1			

Four patients were dose-reduced on study. Two of these patients were dose-reduced due to drug-related AEs: patient 09 (ovarian cancer) was reduced in dose from 20 mg (DL7/8A) to 12 mg (DL6) TdCyd after 1 cycle at the patient’s request due to intolerable grade 2 fatigue; patient 14 (adenoid cystic carcinoma) was dose-reduced from 6 mg (DL5/5A) to 3 mg (DL4) TdCyd after experiencing a DLT during cycle 1 as noted above. Finally, 2 patients were dose-reduced after developing PJP (see details below): patient 10 (malignant paraganglioma) was dose-reduced after 8 cycles from 20 mg (DL7/8A) to 12 mg (DL6) TdCyd; patient 05 (alveolar soft part sarcoma, ASPS) initially on 2 mg TdCyd (DL3/3A) then escalated to 12 mg TdCyd (DL 6) was dose-reduced to 6 mg TdCyd (DL5/5A) on cycle 14.

Five patients withdrew from the study: 2 refused further treatment after experiencing treatment-related anorexia (patient 10), fatigue not attributed to study drug (patient 02) and low grade nausea related to study drug (both patients); 2 had worsening co-morbidities; and 1 switched to an alternative treatment after outside scans showed disease progression (new lesion).

There were 8 deaths on study, 7 of which were attributed to disease progression (Supplementary Table S2). One patient (27; metastatic cholangiocarcinoma) was receiving 14 mg TdCyd on cycle 2 when they were admitted to the hospital with pneumonia, acute kidney injury and worsening thrombocytopenia; TdCyd was held and the patient was started on broad-spectrum multi-agent antibiotic therapy, but died due to septic shock. A contributory role of TdCyd in the death could not be excluded; therefore, a potential causal association with TdCyd was considered.

Two patients were diagnosed with PJP during the study within 2 months of each other and the diagnosis was attributed to the study drug due to no other likely cause. The first patient, receiving 20 mg TdCyd (DL7/8A), was diagnosed on cycle 2 with PJP. This patient was successfully treated for the infection and subsequently received prophylactic PJP treatment starting on cycle 2 until they discontinued TdCyd at their own choice on cycle 10. A second patient, receiving 12 mg TdCyd, was diagnosed on cycle 13 and the diagnosis coincided with a simultaneous identification by bronchoscopy of *M*y*cobacteroides abscessus* which was not unexpected due to the patient’s environmental exposure; the patient remained on study for another 5 cycles, but could no longer travel. Out of an abundance of caution, PJP prophylaxis was subsequently added for all patients in January 2017. After 2 patients experienced serious AEs (respiratory, and gastro-intestinal events which required patient hospitalization) potentially related to Bactrim or Pentamidine, the prophylaxis was removed after discussion with the IRB in November 2018. This decision was also made to support antimicrobial stewardship.

### Clinical outcomes

**Fig. 1:**
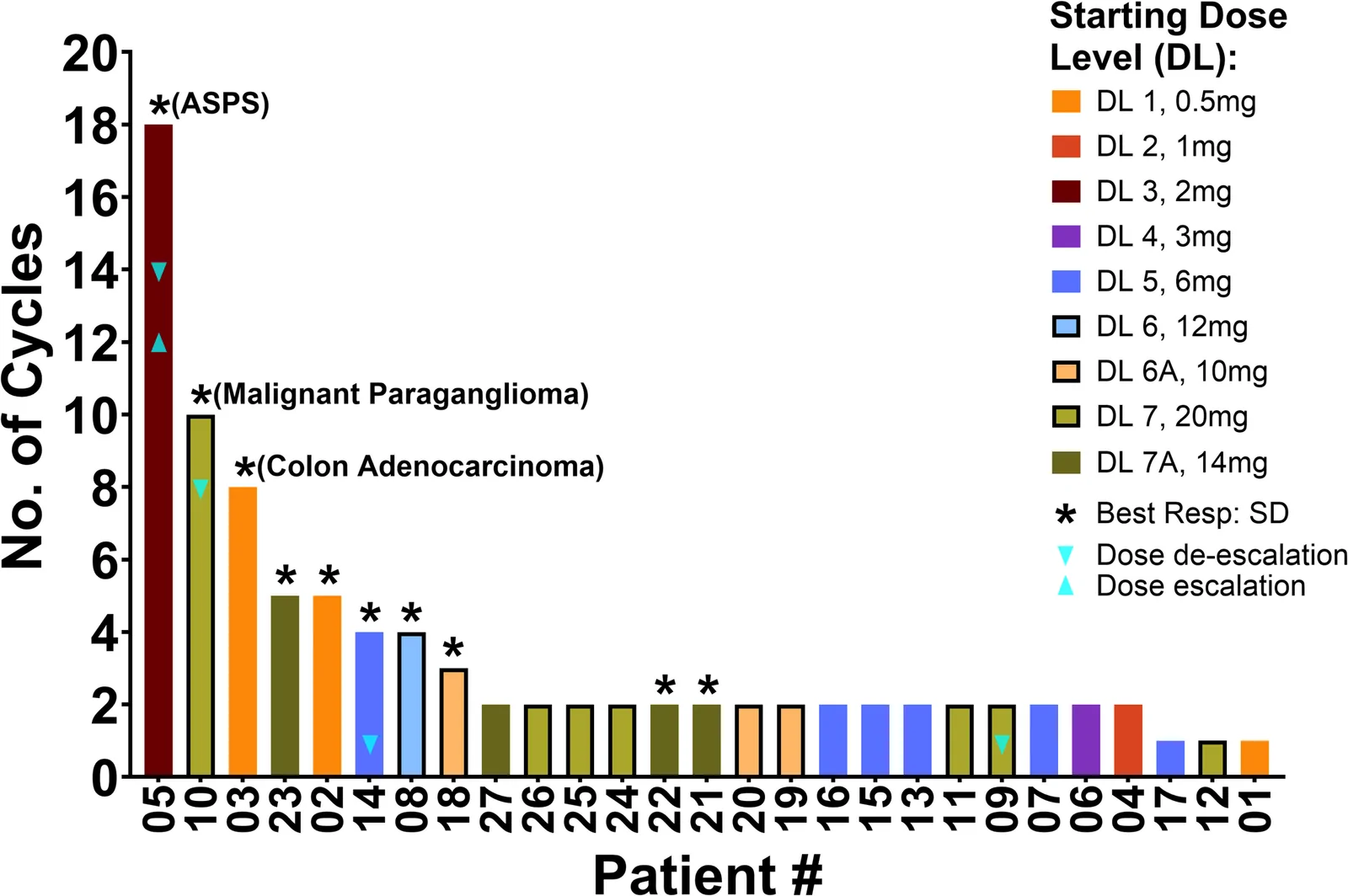
Clinical response to 4’-thio-2’-deoxycytidine (TdCyd) therapy. The number of cycles of treatment completed is shown for each patient on study, color-coded according to starting dose level; dose escalations and de-escalations are marked by teal arrows pointing up and down, respectively, positioned at the cycle when the dose adjustment took place. Asterisks mark the patients with stable disease (SD) as best response. Histology is indicated for patients with SD on study for ≥ 6 cycles. ASPS = alveolar soft part sarcoma

Twenty-three of 27 patients (85.2%) were evaluable for clinical response. The best response was prolonged stable disease (SD) (median = 4.5 cycles; range: 2–18 cycles) recorded in 10 of the 23 evaluable patients (43.5%) (Fig. 1). The median number of prior systemic therapies for patients with a best response of SD was 3 (range 1‒5).

Three patients experienced SD for ≥ 6 cycles including a patient with ASPS (05) who remained on study for 18 cycles, starting on 2 mg TdCyd. This patient was later escalated to 12 mg TdCyd on cycle 12 then dose-reduced to 6 mg TdCyd (cycle 14–onward), before progressing. The 2 other patients with prolonged SD (patients 03 and 10; colon adenocarcinoma and malignant paraganglioma, respectively) were on 0.5 mg TdCyd and 20 mg TdCyd (later de-escalated on cycle 8 to 6 mg TdCyd after developing PJP), respectively. Patients with prolonged SD had at least 2 prior lines of systemic therapy, and at least 2 additional rounds of radiation and/or surgery prior to starting TdCyd.

There was no correlation between the dose of TdCyd and length of response. Nine of the 10 (90%) patients who experienced SD had tumor scans performed; tumor shrinkage (0.5–14%) was reported for 3/9 (33.3%) of these patients, including 1 patient receiving 10 mg TdCyd and 2 patients receiving 14 mg TdCyd (Supplemental Figure S2). No tumor shrinkage was reported for any of the 3 patients with durable SD.

Thirteen of 23 evaluable patients (56.5%) experienced disease progression—of which 7 experienced clinical progression—all within 2 cycles of treatment. Progression occurred predominantly in patients with ≥ 4 prior lines of systemic therapy (median = 4 prior lines of therapy, range 1‒12 for patients with PD, compared with median = 3 prior lines of therapy, range 1‒5 for patients with a best response of SD; *p* = 0.0199, Mann-Whitney test).

Study accrual was put on administrative hold in March 2019 before MTD determination, and a decision was made to conduct additional toxicity and efficacy studies in a canine clinical trial to investigate the effect of TdCyd administration on PJP incidence; the dog studies were discontinued due to low accrual and the clinical study was subsequently closed for administrative reasons.

### Pharmacodynamic analyses

**Fig. 2:**
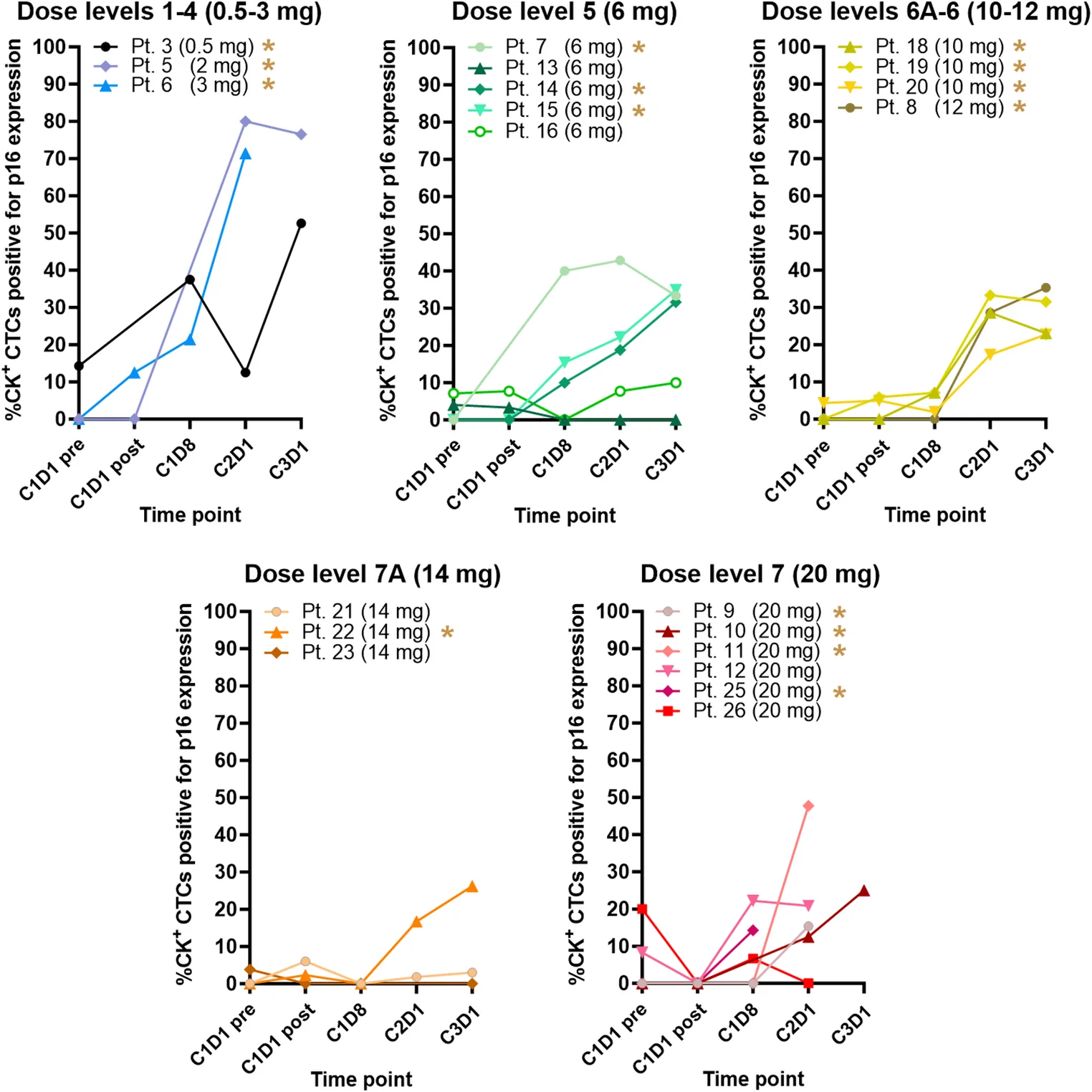
Changes in p16 expression following 4’-thio-2’-deoxycytidine (TdCyd) treatment in cytokeratin-positive (CK^+^) circulating tumor cells (CTCs). Patients were considered evaluable for CK^+^ CTC p16 response if at least one of their baseline specimens (cycle 1 day 1 [C1D1] pre-treatment and/or C1D1 2–4 h post-treatment) and at least one post-treatment specimen (C1D8, C2D1, or later) contained ≥ 6 CTCs. The percentage of CK^+^ CTCs positive for p16—from baseline through Cycle 3 Day 1 (C3D1)—is shown by starting dose level for all patients evaluable for CK^+^ CTC p16 response. All patients who achieved p16 re-expression did so by C3D1. Gold asterisks indicate patients with increased CK^+^ CTC p16 expression, as defined previously [17]. Pt = patient

Because an increase in the percentage of CTCs expressing p16 has been established as a pharmacodynamic biomarker for epigenetic effects of other DNMT inhibitors [17, 18], we measured TdCyd-induced changes in CTC p16 expression. Twenty-one patients were evaluable for assessment of agent-induced changes in the proportion of p16-expressing (p16^+^) CK^+^ CTCs per previously established criteria [17]. Re-expression of p16 was defined as a ≥ 3-fold increase in the percentage of p16-expressing CTCs at any post-treatment time point compared to baseline (wherein a baseline value is the higher of the cycle 1 day 1 pre-treatment [“C1D1 pre”] and C1D1 2–4 h post-treatment [“C1D1 post”] values); for patients with no p16^+^CK^+^ CTCs detected in either baseline sample, an increase in the proportion of p16^+^CK^+^ CTCs was defined as an increase from 0 to ≥ 2 p16^+^CK^+^ CTCs. The majority (15 of 21; 71.4%) of patients evaluable for p16 response in CK^+^ CTCs demonstrated a treatment-associated increase in the proportion of p16^+^CK^+^ CTCs, including all 3 patients with durable SD: patients 03, 05, and 10, who were on study for 8, 18, and 10 cycles, respectively (Fig. 2). For both patients 03 and 05, the percentage of p16^+^CK^+^ CTCs peaked at ~ 80% after 8 and 7 cycles of treatment, respectively (Supplementary Figure S3). For patient 05, the percentage of p16^+^CK^+^ CTCs peaked before the dose was escalated from 2 mg to 12 mg TdCyd and subsequently dropped to 20‒30% for the remaining duration of treatment, regardless of the TdCyd dose administered. For patient 10, the proportion of p16^+^CK^+^ CTCs increased slowly and constantly, peaking at ~ 40% on cycle 10, and was unaffected by the TdCyd dose reduction from 20 mg to 6 mg on cycle 8 (Supplementary Figure S3). Two of the 3 patients with reported tumor shrinkage (patients 14 and 18) had p16 re-expression in their CK^+^ CTCs by cycle 3, day 1; no p16 re-expression was recorded in the third patient (patient 23). There was no correlation between the tumor shrinkage level and the percentage of p16^+^CK^+^ CTCs in the patients’ blood. Notably, increases in the proportion of p16^+^CK^+^ CTCs occurred in patients at every dose level in which there were patients assessable for p16^+^ CK^+^ CTC response, including all 3 such assessable patients receiving the lowest dose levels (DL1, DL3/3A, and DL4; 0.5, 2, and 3 mg TdCyd, respectively). For p16^+^ CK^+^ CTC numbers recorded across the full time-course, see Supplementary Figure S3.

We also assessed p16 re-expression in another clinically significant CTC population—vimentin-positive (V+), mesenchymal-like phenotype CTCs, which are associated with poor prognosis in various tumor types [17, 22, 23]. Six of the 16 patients (37.5%) evaluable for V^+^ CTC p16 response demonstrated p16 re-expression in this CTC population (Supplementary Figure S4)—substantially lower than the prevalence measured in the CK^+^ CTC population. P16 re-expression in V^+^ CTCs also occurred in 2 patients with prolonged SD (05 and 10). There was no correlation between the dose of TdCyd treatment and V^+^ CTC p16 re-expression.

### Pharmacokinetic analyses

**Fig. 3:**
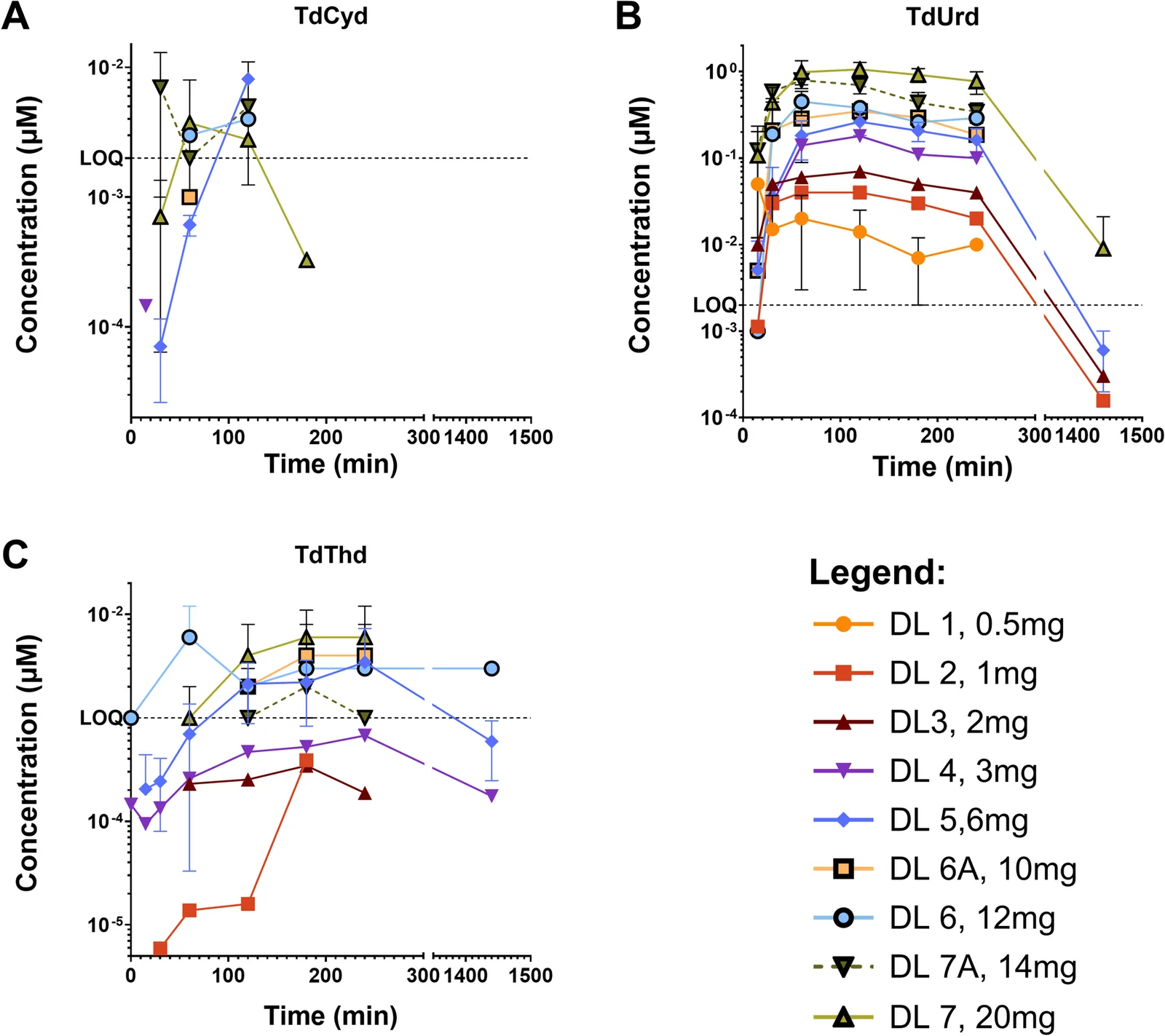
Plasma pharmacokinetic analysis of 4’-thio-2’-deoxycytidine. Mean patient plasma levels of 4’-thio-2’-deoxycytidine (TdCyd) **(A)** and metabolites 4’-thio-2’-deoxyuridine (TdUrd) **(B)** and 4’-thio-2’-deoxythymidine (TdThd) **(C)**. Error bars = standard deviation. DL = dose level. Sample size: n(DL1) = 3; n(DL2) = 1; n(DL3/3A) = 1; n(DL4) = 1; n(DL5/5A) = 7; n(DL6A) = 3; n(DL6) = 2; n(DL7A) = 4; n(DL7/8A) = 7. The following patients were evaluated at two dose levels: patient 05 (DL3/3A and DL6); and patient 10 (DL7/8A and DL5/5A). LOQ = limit of quantification

TdCyd plasma levels were below the limit of quantification (LOQ) for the majority of samples analyzed (Fig. 3A) and rapid TdCyd metabolism following delivery precluded the calculation of the elimination half-life (Supplementary Table S3). Plasma levels of metabolite TdUrd exhibited a largely dose-proportional profile, peaked at approximately 1 µM 60 min post TdCyd administration, and the elimination half-life was 7.2 h (Fig. 3B); while there was no clear correlation between the TdCyd dose administered and Cmax values, the highest median Cmax values were detected at the highest TdCyd dose administered (Supplementary Table S3). In contrast, TdThd metabolite plasma levels exceeded the LOQ for TdCyd doses ≥ 10 mg, and also exhibited a largely dose-proportional profile (Fig. 3C); the elimination half-life could not be calculated. Higher levels of TdUrd and TdThd metabolite were detected in patients with a greater number of grade ≥ 2 AEs possibly related to study drug (Supplementary Table S2), although the study was not powered to evaluate a formal association between AE incidence and metabolite plasma concentration.

TdCyd could not be detected in the urine of patients receiving treatment. The only metabolite recovered in urine was TdUrd and there was no correlation between the percentage of TdUrd recovered in urine and the TdCyd dose administered (Supplementary Table S3).

## Discussion

TdCyd offers an improvement over FDA-approved DNA methyltransferase inhibitors by virtue of a higher incorporation rate into DNA without inhibition of DNA synthesis [9, 11]. Preclinical studies showed that treatment with TdCyd at doses that were well tolerated in extended schedules inhibited tumor growth in preclinical mouse models due to DNMT1 depletion [9, 24], but biopsies were not collected on this study to confirm this mechanism of action.

Our clinical study enrolled 27 heavily pre-treated patients (3 median prior lines of systemic therapy, range 1–12), of which 23 were evaluable for response. Study accrual was put on hold, and a canine clinical trial was opened to investigate the effect of TdCyd administration on PJP incidence; the dog studies were discontinued due to low accrual and the clinical study was subsequently closed for administrative reasons, before an MTD could be determined, and no dose-efficacy relationship could be determined. The highest tested dose was 20 mg TdCyd. Although our study was not powered to define an optimal dose-range, we noted that TdCyd dose levels of 6 mg and 10 mg appeared to be the best tolerated (Supplementary Table S2); we therefore propose that DL5/5A and DL6A (6–10 mg TdCyd) should be further studied as starting doses in future dose-range finding trials. Doses above 14 mg TdCyd (DL7A) resulted in higher rates of hematological toxicities, which is a known class effect [25]. The most common grade 3/4 AEs possibly related to study drug—lymphopenia, thrombocytopenia and increased aspartate aminotransferase levels—are consistent with the drug class safety profile. There was 1 DLT on study: patient 14 (adenoid cystic carcinoma) receiving 6 mg TdCyd experienced grade 3 transaminitis on cycle 1.

Two patients, both negative for HIV-infection, developed PJP while on study, an opportunistic fungal infection that can cause severe lung illness particularly in immunocompromised patients [26]. PJP occurrence was difficult to attribute due to multiple co-morbid factors in the diagnosed patients, and resulted in significant protocol changes: from the incorporation of PJP prophylaxis at initiation, to the extension of the DLT assessment period. No new PJP cases were reported after the introduction of PJP prophylaxis; however the additional treatment was associated with multiple AEs possibly related to Bactrim or Pentamidine, which led to re-evaluation of the risk/benefit profile of the prophylaxis treatment and its subsequent removal from the protocol.

The best recorded response was SD in 10 of the 23 evaluable patients (43.5%), with 3 patients experiencing prolonged SD ≥ 6 cycles. One patient (ASPS) received 3 prior lines of systemic therapy, none lasting longer than 8 months; the patient remained on TdCyd treatment for 18 cycles and was taken off study following disease progression. A patient with malignant paraganglioma with 2 prior lines of systemic therapy came off study at the patient’s choice after 10 cycles; finally, a patient with colon adenocarcinoma who had received 3 prior lines of systemic treatment came off after 8 cycles. The duration of SD was not dose dependent. Rapid progression occurred predominantly in patients with ≥ 4 prior lines of systemic therapy (median = 4, range 1‒12) compared with median = 3 prior lines of therapy (range 1‒5) for patients with a best response of SD regardless of the TdCyd dose administered, consistent with a study population with an overall poor prognosis. However, we consider this an unfavorable factor which may be generally prognostic rather than predictive for TdCyd in particular.

Because demonstration of DNMT1 target engagement can be difficult due to interpatient heterogeneity in DNMT1 degradation and rebound kinetics in response to DNMT inhibitors [18, 27], and in light of preclinical pharmacodynamic data demonstrating substantial heterogeneity in TdCyd-induced DNMT1 decreases across responsive xenograft models (data not shown), we instead evaluated TdCyd-induced epigenetic changes by measuring CTC p16 expression—an established pharmacodynamic biomarker of the epigenetic effects of other DNMT inhibitors [17, 18]. While the small sample sizes preclude definitive conclusions from our CTC analyses, these data suggest that TdCyd has epigenetic activity in CK^+^ CTCs even at the lowest doses tested. Interestingly, dose adjustments do not appear to affect the pharmacodynamic response, with p16^+^CK^+^ CTCs percentages peaking prior to TdCyd dose escalation (e.g. patient 05, Supplementary Figure S3) and the percentage remaining relatively stable after dose reductions. There was no correlation between tumor shrinkage and the percentage of p16^+^CK^+^ CTCs after treatment. Based on these findings, we hypothesize that further evaluation of low-dose TdCyd combination therapies can be valuable, and that future studies can monitor TdCyd-mediated epigenetic modulation as a potential marker for drug activity. The discrepancy in the prevalence of TdCyd-induced p16 re-expression between CK^+^ and V^+^ CTCs has been noted in other DNMT inhibitor trials and may reflect the generally more resistant phenotype of mesenchymal-like V^+^ CTCs [17, 18]. Importantly, p16 demethylation in tumor has been confirmed in preclinical studies at doses as low as 1 mg/kg and 2 mg/kg (human equivalent dose of 3 mg/m^2^ and 6 mg/m^2^, respectively) (data not shown). While these preclinical and clinical results do not constitute proof of direct TdCyd target engagement, and the pharmacodynamic analysis was designed to be hypothesis generating not confirmatory, they indicate that lower and prolonged exposure to TdCyd warrants further evaluation to determine if clinical efficacy and tolerability could be improved without increasing TdCyd dosing.

At the time of trial launch, no studies had investigated the distribution, excretion, or metabolism of TdCyd in humans, prompting us to explore the pharmacokinetics of TdCyd in study participants. TdCyd was consistently detected in plasma at dose levels exceeding 6 mg and exceeded the LOQ for doses above 12 mg. TdUrd was the primary circulating TdCyd metabolite and demonstrated a largely dose-proportional plasma pharmacokinetic profile; its presence was predicted by preclinical studies and indicate that TdCyd is a good substrate for Cyd deaminase and a poor substrate for dThd phosphorylase [24] but is a substrate for thymidine kinase, and as the mono-phosphate, a substrate for thymidine synthetase. Of note was the presence of the TdThd metabolite, detected at plasma concentrations above the LOQ ~ 2–3 h post drug administration for patients receiving 10 mg TdCyd (DL6A) or more, and higher TdThd plasma concentrations were detected in patients with higher incidence and severity of AEs possibly related to study drug. The study was not powered to evaluate a formal association between TdThd plasma concentration and toxicity, which may be evaluated in a future trial. These observations agree with previous studies conducted in mice, that showed TdUrd and TdThd plasma concentrations increased quickly, plateaued within 2 h and remained relatively stable for up to 24 h post IP or oral drug administration [24]. Preclinical studies at the NCI show that TdCyd and the TdThd metabolite are incorporated in the DNA, indicating a mechanism of action similar to that of gemcitabine; however, TdCyd and TdThd do not appear to act as chain terminators (data not shown). Importantly, thymidine metabolites were not routinely detected in the plasma of patients receiving second generation deoxycytidine-derivative FdCyd [17, 18]; we hypothesize that TdThd accumulation contributed to TdCyd-related AEs in our current study. Future pharmacokinetic studies will be needed, to verify a potential contribution of TdThd accumulation to TdCyd toxicity and to evaluate the mechanism by which TdCyd metabolism affects DNMT1 depletion. The addition of potent inhibitors of cytidine deaminase such as tetrahydrouridine (THU) [9] may reduce TdThd formation and improve TdCyd tolerability. However, our preclinical data indicated that THU co-administration dramatically increased TdCyd exposure (AUCs up to 20-fold greater; data not shown), while decreasing TdCyd’s antitumor effects [8]. The decision was therefore made to not include THU in the clinical plan. Our previous clinical experience also indicated that THU exposure in conjunction with nucleoside analogue FdCyd is associated with higher DLT occurrence, possibly due to prolonged exposure to the deoxycytidine analogue and increased generation of phosphorylated metabolites [18].

While the study showed that TdCyd can be safely administered at least up to doses of 20 mg, increasing the TdCyd dose did not improve efficacy and amplified the number of drug-related AEs and the plasma levels of potentially toxic TdThd. Furthermore, p16 re-expression in CK^+^ CTCs, a biomarker of epigenetic activity of other DNMT inhibitors, was recorded at all tested doses, although no correlation with drug efficacy and length of response could be determined for this exploratory endpoint. We hypothesize that longer exposures to low TdCyd concentrations may be beneficial for patients; this is also acknowledged by recent paradigm shifts for dose optimization and dose selection of novel targeted oncology drugs [28]. Previous reports also indicate that prolonged administration of DNMT1 inhibitors may be required for the development and/or maintenance of clinical responses, including delayed responses [8, 29], and could be explained by the fact that DNMT1-depleting agents require multiple rounds of cell division to re-express tumor suppressor genes [9]. Furthermore, in our study doses below 14 mg TdCyd were better tolerated by patients. Future TdCyd studies will require flexible statistical designs to explore delayed toxicity and efficacy that allow the definition of an optimal biological dose instead of an MTD [30]. As efficacy may be influenced by prognostic factors, randomizing patients to various doses would be considered in future dose-finding studies for TdCyd.

Ongoing clinical studies at the NIH are also investigating alternative second generation hypomethylating agents, such as 5-aza-4’-thio-2’-deoxycytidine (Aza-TdC) (NCT03366116), which has shown higher efficacy as single agent [13] or in combination with TdCyd in preclinical studies (data not shown). Additional clinical avenues for TdCyd-based therapies in solid tumors could include combination therapies targeting immune activation—such as combinations with JAK1/2 small molecule inhibitors [31], or immune checkpoint inhibitors [32]—or combinations with other epigenetic modulators such as HDAC3 inhibitors [33] and EZH1/2 inhibitors [34].

## Supplementary Information

Below is the link to the electronic supplementary material.

Supplementary Material: Redacted Protocol Version Date November-16-2023

Supplementary Figure S2: Percentage change from baseline in tumor size over time for patients receiving TdCyd. Sixteen patients had available tumor measurements. Dotted lines represent the threshold for partial response and progressive disease according to RECIST 1.1 guidelines. Pt = patient.

Supplementary Figure S3: Changes in p16 expression following 4'-thio-2'-deoxycytidine (TdCyd) treatment in cytokeratin-positive (CK+) circulating tumor cells (CTCs) (full time course). Patients were considered evaluable for CK+ CTC p16 response if at least one of their baseline specimens (cycle 1 day 1 [C1D1] pre-treatment and/or C1D1 2-4 hours post-treatment) and at least one post-treatment specimen (C1D8, C2D1, or later) contained ≥ 6 CTCs. The percentage of CK+ CTCs positive for p16 for each collected sample is shown by starting dose level for all patients evaluable for CK+ CTC p16 response. Gold asterisks indicate patients with increased CK+ CTC p16 expression, as defined previously [17]. Pt = patient.

Supplementary Figure S1: Chemical formulas of 4'-thio-2'-deoxycytidine (TdCyd) and its main metabolites 4'-thio-2'-deoxyuridine (TdUrd), and 4'-thio-2'-deoxythymidine (TdThd).

Supplementary Figure S4: Changes in p16 expression following 4'-thio-2'-deoxycytidine (TdCyd) treatment in vimentin-positive (V+) circulating tumor cells (CTCs). Patients were considered evaluable for V+ CTC p16 response if at least one of their baseline specimens (cycle 1 day 1 [C1D1] pre-treatment and/or C1D1 2-4 hours post-treatment) and at least one post-treatment specimen (C1D8, C2D1, or later) contained ≥ 6 CTCs. The percentage of V+ CTCs positive for p16—from baseline through Cycle 3 Day 1 (C3D1)—is shown by starting dose level for all patients evaluable for V+ CTC p16 response. All patients who achieved p16 re-expression did so by C3D1. Gold asterisks indicate patients with increased V+ CTC p16 expression, as defined previously [17]. Pt = patient.

Supplementary Tables

## Data Availability

The original data presented in the study are included in the article/supplementary material. For further inquiries contact the corresponding authors.
