# Supplementary material for: A phase 1 study of 4’-thio-2’-deoxycytidine in patients with solid tumors
Source: Cancer Chemother Pharmacol. 2026 Nov 5;96(1):129. doi: 10.1007/s00280-026-04969-8 (PMC13739968; doi:10.1007/s00280-026-04969-8)
Supplement: Supplementary file 1 [file 280_2026_4969_MOESM1_ESM.pdf]

CTEP #9883
Clinical Center #15-C-0116

Phase I Trial of 4'-thio-2'-deoxycytidine (TdCyd) in Patients With Advanced Solid Tumors

Abbreviated Title: Ph I TdCyd

Coordinating Center: Developmental Therapeutics Clinic (NCIDTC)
National Cancer Institute
10 Center Drive
Bethesda, MD 20892

Principal Investigator: James H. Doroshow, MD[A-E], DCTD/NCI
[redacted]

Study Sponsor: DCTD, NCI

NCI Supplied Agent: 4'-thio-2'-deoxycytidine (TdCyd) (NSC 764276)

IND Number: 125208

NCT Number: NCT02423057

Version Date: November 16, 2023

Roles: [A]obtains information by intervening or interacting with living individuals for research purposes; [B]obtains identifiable private information about living individuals; [C]obtains the voluntary informed consent of individuals to be subjects; [D]makes decisions about subject eligibility; [E]studies, interprets, or analyzes identifiable private information or data/specimens for research purposes

PRÉCIS

Background:

- Methylation-mediated silencing of genes is an epigenetic mechanism implicated in carcinogenesis; agents that inhibit this mechanism are of clinical interest because of their potential to re-activate silenced tumor suppressor genes. Two DNA hypomethylating nucleosides, 5-azacytidine (azacytidine) and 5-aza-2'-deoxycytidine (decitabine) have been approved by the FDA for the treatment of patients with myelodysplastic syndromes and certain leukemias.
- The nucleoside analog 4'-thio-2'-deoxycytidine (TdCyd) is incorporated into DNA where it engages the active site of DNA methyltransferase I (DNMT1), a maintenance methyltransferase that contributes to the hypermethylation and silencing of tumor suppressor genes. DNMT1 can become trapped in a covalent complex with DNA, thus depleting free enzyme and inhibiting the normal maintenance methylation of CpG sites, resulting in re-activation of tumor suppressor genes.
- Data generated at Southern Research Institute and the NCI suggest a correlation between TdCyd activity in solid tumor xenograft models and decreased levels of DNMT1.
- TdCyd offers an improvement over current DNA methyltransferase inhibitors by virtue of a higher incorporation rate into DNA at lower levels of cytotoxicity; treatment with TdCyd is anticipated to result in the inhibition of tumor growth due to DNMT1 depletion at oral doses that are well tolerated in extended dosing schedules.
- Two patients on dose levels 6 and 7 developed *Pneumocystis jiroveci* pneumonia (PJP), so all patients will be monitored for PJP and treated as appropriate.

Primary Objective:

- To establish the safety, tolerability, and MTD of oral TdCyd administered daily for 5 days a week for 2 weeks, with one week off, q 21-day cycles, to patients with refractory solid tumors

Secondary Objectives:

- To determine the pharmacokinetics of oral TdCyd
- To document preliminary evidence of TdCyd activity
- To determine effect of study treatment on re-expression of select genes silenced by methylation in circulating tumor cells

Eligibility:

- Patients must have histologically documented solid tumors whose disease has progressed on standard therapy or for which there is no available standard therapy

Study Design:

- TdCyd will be administered orally once a day for 5 days of each week for 2 weeks, with one week off, in 21-day cycles.
- The trial will follow an accelerated titration design, changing to a traditional 3+3 dose escalation design (3-6 patients per cohort) once specified toxicity criteria are met. Intrapatient dose escalation will be allowed.
- Blood samples will be obtained for pharmacokinetic analysis and to isolate circulating tumor cells to assess re-expression of genes silenced by methylation.

SCHEMA

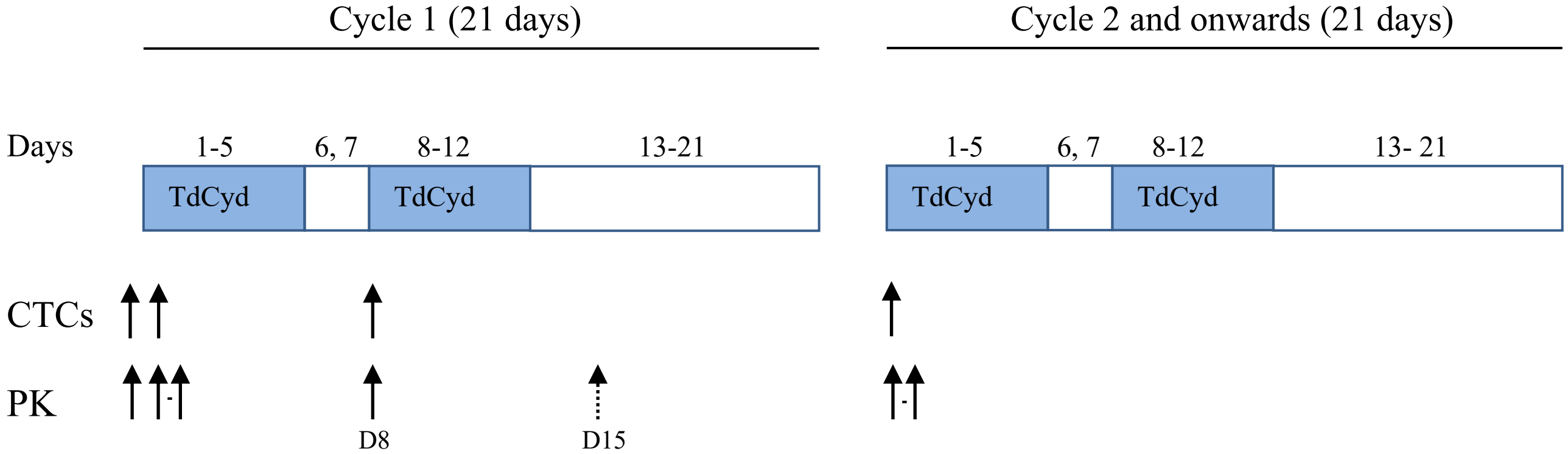

TdCyd will be administered orally once a day on days 1-5 of each week for 2 weeks, with one week off, in 21-day cycles.

Blood samples for CTCs will be collected prior to TdCyd administration on day 1 and then 2 to 4 hours post dose on day 1, on day 8 (at any time; this sample is optional), and in cycle 2 on day 1 (at any time). Additional samples will be collected on day 1 of all subsequent cycles and at time of disease progression.

Blood samples for PK analysis will be collected prior to TdCyd administration day 1 and then 1, 4, and 24 hours after agent administration (before another dose is taken) during cycle 1. Blood samples will also be collected on cycle 1 day 8 and day 15 (day 15 sample is optional), and on cycle 2 day 1 prior to TdCyd administration and then 1, 4, and 24 hours after agent administration.
*Patients who have been dose-adjusted will have optional PK samples collected prior to TdCyd administration on day 1 and then 1, 2, and 24 hours after drug administration (before another dose is taken) during cycle 1 only.

Table for Accelerated Titration Design

Dose Level	TdCyd
-1	0.5 mg for 5 days a week for *one* week, q21-day cycle
**1**	0.5 mg
**2**	1 mg
**3**	2 mg
**4**	3 mg
**5**	6 mg
**6**	12 mg
**7**	20 mg

Table for 3+3 design (after Amendment F)

Dose Level	TdCyd
**-2 (3A)**	2 mg
**-1 (4A)**	4 mg
**5A**	6 mg
**6A**	10 mg
**7A**	14 mg
**8A**	20 mg
**9A**	30 mg
**10A**	40 mg
**11A**	60 mg

TABLE OF CONTENTS

PRÉCIS 2

SCHEMA 3

1 OBJECTIVES 6

2 BACKGROUND 6
- **2.1** Methylation in Cancer 6
- **2.2** TdCyd 7
- **2.3** Preclinical Efficacy Studies 8
- **2.4** Pharmacodynamic Studies 9
- **2.5** Preclinical Pharmacology and Toxicology 10
- **2.6** Correlative Studies 16
- **2.7** Clinical Status 17
- **2.8** Rationale 18

3 PATIENT SELECTION 18
- **3.1** Eligibility Criteria 18
- **3.2** Exclusion Criteria 19
- **3.3** Inclusion of Women and Minorities 20
- **3.4** Research Eligibility Evaluation 20

4 REGISTRATION PROCEDURES 20
- **4.1** Registration Process 20

5 TREATMENT PLAN 21
- **5.1** Agent Administration 22
- **5.2** Definition of Dose-Limiting Toxicity 22
- **5.3** General Concomitant Medication and Supportive Care Guidelines 24
- **5.4** Duration of Therapy 26
- **5.5** Duration of Follow Up 27
- **5.6** Criteria for Removal from Study 27

6 DOSING DELAYS/DOSE MODIFICATIONS 27
- **6.1** Dose Modifications 27

7 ADVERSE EVENTS: LIST AND REPORTING REQUIREMENTS 28
- **7.1** Comprehensive Adverse Events and Potential Risks 28
- **7.2** Safety Reporting Requirements/Data and Safety Monitoring Plan 29
- **7.3** Expedited Adverse Event Reporting 29
- **7.4** NCI-IRB and Clinical Director Reporting 34
- **7.5** Secondary Malignancy 35
- **7.6** Second Malignancy 35

8 PHARMACEUTICAL INFORMATION 35
- **8.1** 4’-thio-2’-deoxycitidine (NSC 764276) 35
- **8.2** Agent Ordering 36
- **8.3** Agent Accountability 37

9 CORRELATIVE/SPECIAL STUDIES 37
- **9.1** Pharmacokinetic Assays 37

9.2 Pharmacodynamic Assays 39

10 STUDY CALENDAR 41

11 MEASUREMENT OF EFFECT....43
11.1 Antitumor Effect – Solid Tumors 43
11.2 Guidelines for Evaluation of Measurable Disease....45
11.3 Response Criteria....47
11.4 Duration of Response....49

12 DATA REPORTING / REGULATORY REQUIREMENTS....49
12.1 Data Reporting....49
12.2 CTEP Multicenter Guideline 50
12.3 Collaborative Agreements Language....50

13 STATISTICAL CONSIDERATIONS....50
13.1 Sample Size/Accrual Rate....50
13.2 Analysis of Secondary Endpoints 50

14 HUMAN SUBJECTS PROTECTIONS 50
14.1 Justification for Subject Selection 50
14.2 Evaluation of Benefits and Risks/Discomforts....51
14.3 Consent and Assent Process and Documentation 51
14.4 Procedure for Protecting Against or Minimizing Any Potential Risks....52
14.5 Patient Advocate 52

REFERENCES 53

APPENDIX A: Performance Status Criteria 55

APPENDIX B: Patient Study Diary 56

APPENDIX C: Patient Study Calendar....57

APPENDIX D: Procedure for Blood and Urine Samples for Pharmacokinetics....58

APPENDIX E: PK/PD Sample Collection Worksheets 61

1 OBJECTIVES

Primary Objective:

- To establish the safety, tolerability, and MTD of oral TdCyd administered daily for 5 days a week for 2 weeks, with one week off, in 21-day cycles, to patients with refractory solid tumors

Secondary Objectives:

- To determine the pharmacokinetics of oral TdCyd
- To document preliminary evidence of TdCyd activity
- To determine effect of study treatment on re-expression of select genes silenced by methylation in circulating tumor cells

2 BACKGROUND

2.1 Methylation in Cancer

Methylation-mediated silencing of genes is one epigenetic mechanism implicated in cancer [1-4]. Hypermethylation of cytosines at CpG sites in the regulatory sequences of tumor suppressor genes silences them in a manner akin to inactivating mutations, and is recognized to be far more frequent than classical genetic mutation [5-7]. Inhibition of this mechanism is established as useful for cancer therapy, and two DNA hypomethylating nucleosides (5-azacytidine and 5-aza-2'-deoxycytidine) have been approved by the FDA for the treatment of patients with myelodysplastic syndromes and certain leukemias [8].

These nucleoside analogs are incorporated into DNA and engage the active site of DNA methyltransferase I (DNMT1), but in the absence of an N5-cytosine proton, DNMT1 can become trapped in a covalent complex with DNA. Activation of proteasome-mediated degradation pathways further depletes free enzyme and inhibits normal maintenance methylation of CpG sites. The existing methylation pattern on genes is no longer maintained in replicated cells, resulting in reactivation of tumor suppressor (and other) genes.

DNMT inhibitors represent a promising class of epigenetic modulators by causing gene methylation change, growth arrest, differentiation and apoptosis or cell death. Anti-tumor activity of DNMT inhibitors has been demonstrated largely in myelodysplastic syndromes and acute myeloid leukemia in unselected patient populations, and less frequently in human solid tumors.

Optimal drugs of this class should not excessively inhibit DNA synthesis, because the process described above requires incorporation of these agents into DNA and at least two cycles of cell division to maximize the re-expression of silenced genes [9]. Long exposures to relatively low concentrations may be an optimal way to deliver these agents. The azacytidine analogs have several chemical and biochemical deficiencies (relatively inefficient incorporation into DNA, DNA synthesis inhibition, chemical instability, and metabolic instability due to deamination).

Thus, there is a need for DNA hypomethylating compounds with improved properties in patients with solid tumors.

2.2 TdCyd

4'-Thio-2'-deoxycytidine (TdCyd, NSC 764276) is a cytidine analog in which the 4'-O moiety in deoxyribose has been replaced with sulfur. Southern Research Institute first synthesized this compound in 1991 and have since shown it to have growth-inhibitory and cytotoxic activity against several cancer cell lines (IC_{50} 0.6-0.88 μM) [10]. A subsequent study demonstrated that TdCyd is robustly phosphorylated and incorporated into the DNA of human leukemia cells (CCRF-CEM) at rates exceeding those of the natural nucleoside or ara-analogs (Figure 1) [11]. Studies conducted in the Developmental Therapeutics Program, DCTD, confirmed that TdCyd and its metabolite thiothymidine are both incorporated into DNA: the rate of TdCyd incorporation into NCI-H23 cells increased over 24 to 96 hours, reaching about 12% substitution for endogenous dCyd. Global DNA methylation (i.e., the ratio of methyl-dCyd to dCyd in a digest of all DNA) was unaffected by TdCyd [12].

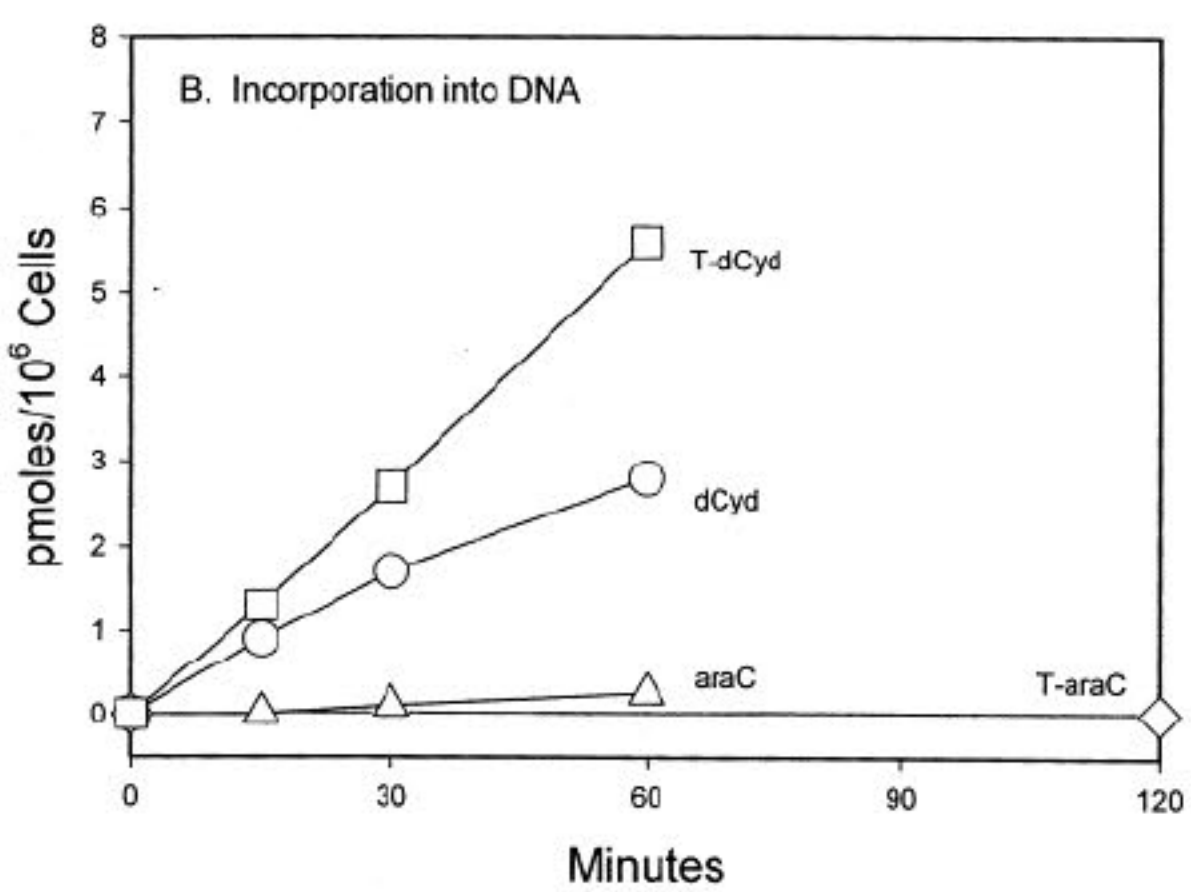

Figure 1. CEM cells incubated with 100 nM [5-^{3}H]dCyd, [5-^{3}H]T-dCyd, [5-^{3}H]araC, or [5-^{3}H]T-araC. Radiolabel incorporation was determined over time [10].

Use of TdCyd as an inhibitor of DNA methylation in human cancer cells was described in a report showing that when TdCyd was incorporated into synthetic DNA at the recognition site for the bacterial DNA methyltransferase, M.*HhaI*, methylation was markedly inhibited, even though the nucleobase in TdCyd was unmodified [13]. Further studies at Southern Research Institute showed that treatment of CCRF-CEM or KG1a leukemia or NCI-H23 lung cancer cell lines with TdCyd for 96 hr resulted in marked depletion of DNMT1 protein at nanomolar to low micromolar concentrations (Figure 2) [14].

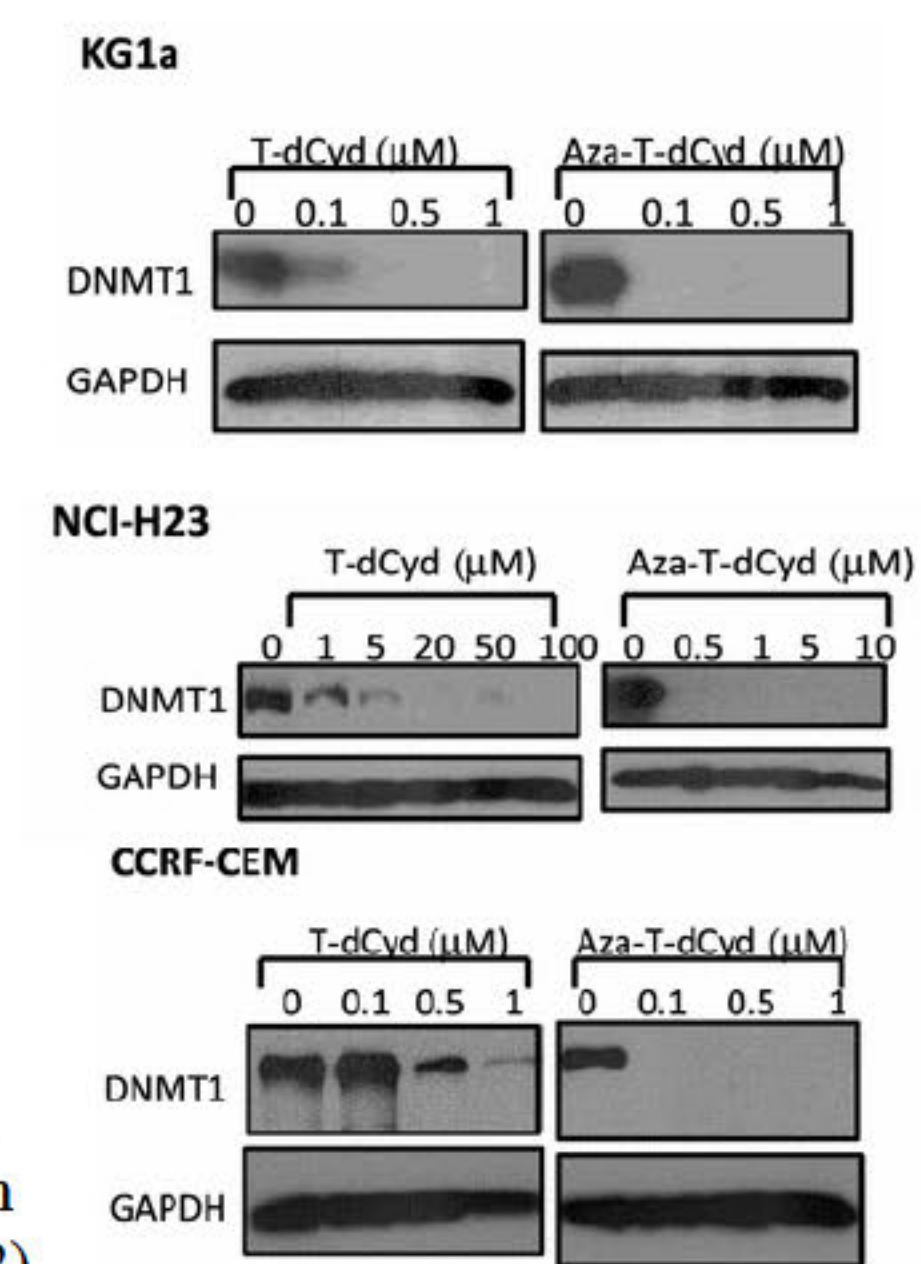

Figure 2

KG1a leukemia cells were used to investigate the effects of TdCyd on DNA methylation of a specific tumor suppressor gene, p15 [14]. These cells contain an aberrant methylation pattern in

the p15 gene that is associated with acute myeloid leukemias. Using methylation-specific PCR and bisulfite-modified DNA with primers specific for unmethylated DNA and methylated DNA, it was demonstrated that TdCyd (3 µM, 72 hr) is capable of demethylating the p15 CpG island similar to the approved cytidine analog, decitabine (1 µM). It was further shown that this effect led to the re-expression of the p15 gene as shown by semi-quantitative RT-PCR analysis.

2.3 Preclinical Efficacy Studies

Initial in vivo studies were performed by Southern Research Institute. The Biological Testing Branch (BTB), DCTD, has also evaluated TdCyd in several human tumor xenograft models to measure antitumor activity and to obtain tumor samples for pharmacodynamic assay development. TdCyd had efficacy (a T/C value of 40% or less) in the SK-ES1 Ewing sarcoma and NCI-H23 non-small cell lung cancer (NSCLC) preclinical models using the extended dosing schedules anticipated to allow incorporation of the agent into DNA. In the SK-ES1 Ewing model, TdCyd given orally at 4.0 mg/kg on a QDx5 schedule, repeated for a total of four cycles, resulted in partial tumor growth inhibition and an optimal T/C of 28%. Figure 3 shows inhibition of tumor growth in the NCI-H23 model by TdCyd, whereas decitabine has no activity.

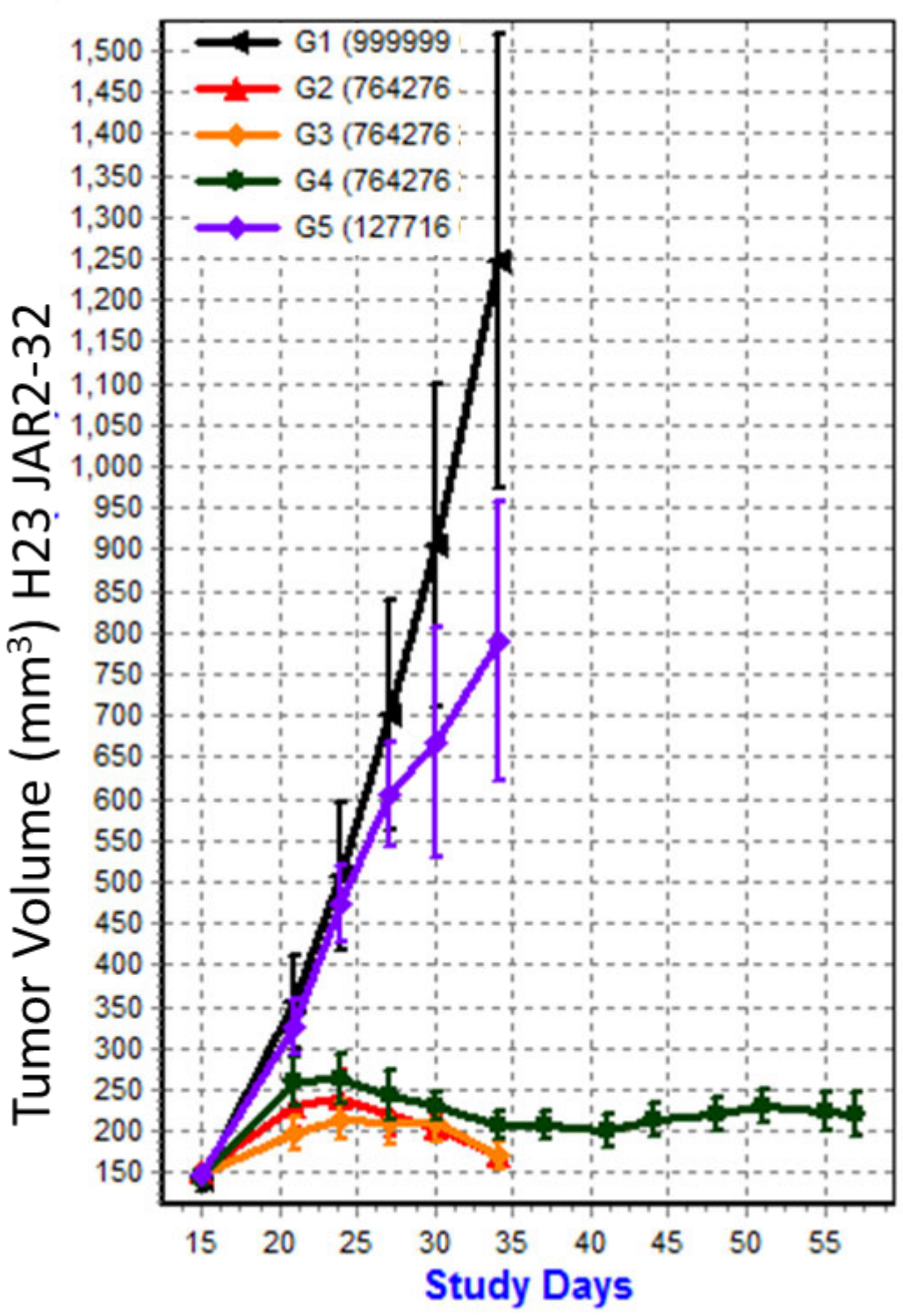

Figure 3: In the NCI-H23 NSCLC model, TdCyd was given orally at 2 or 4 mg/kg/dose on a QDx5 schedule, repeated for 3 cycles, or at 2 mg/kg on a QDx14 schedule, repeated for 2 cycles. As a control, decitabine was given IP at 0.75 mg/kg on a QDx5 schedule, repeated for 3 cycles (the NCI-H23 model is insensitive to decitabine).
TdCyd was well tolerated by the mice in these experiments. Similar tumor growth inhibition was observed at 2 and 4 mg/kg (T/C values of 30-31%). On the QDx14 schedule, TdCyd produced minimal (4.1%) weight loss, no lethality, and a T/C value of 17%. These results indicate that TdCyd has activity in a tumor model that is insensitive to decitabine.

Vehicle control qdx5
TdCyd 4 mg/kg qdx5
TdCyd 2 mg/kg qdx5
TdCyd 2 mg/kg qdx14
Decitabine 0.75 mg/kg qdx5 IP

TdCyd had modest activity in M14 melanoma, SK-OV3 ovarian, MiaPaCa-2 pancreatic, H522 lung, HCT-116 colon, and MALME3 melanoma models, but was inactive in the OVCAR-5 ovarian, COLO205 colon carcinoma, or SW48 colon adenocarcinoma models (data not shown).

2.4 Pharmacodynamic Studies

Levels of DNMT1 were measured in pre- and post-dose tumor samples collected on the preclinical xenograft model studies described above. Immunohistochemical (IHC) analysis of TdCyd-responsive H23 xenograft tumors from the efficacy experiments shown in Figure 3 (QDx5 and QDx14 dosing schedules) revealed that DNMT1 levels were depleted by ~60% following treatment (mean staining index, 56.7 versus 23.0; P<0.001; 2-sided t test). This result was confirmed by Western blot (Figure 4).

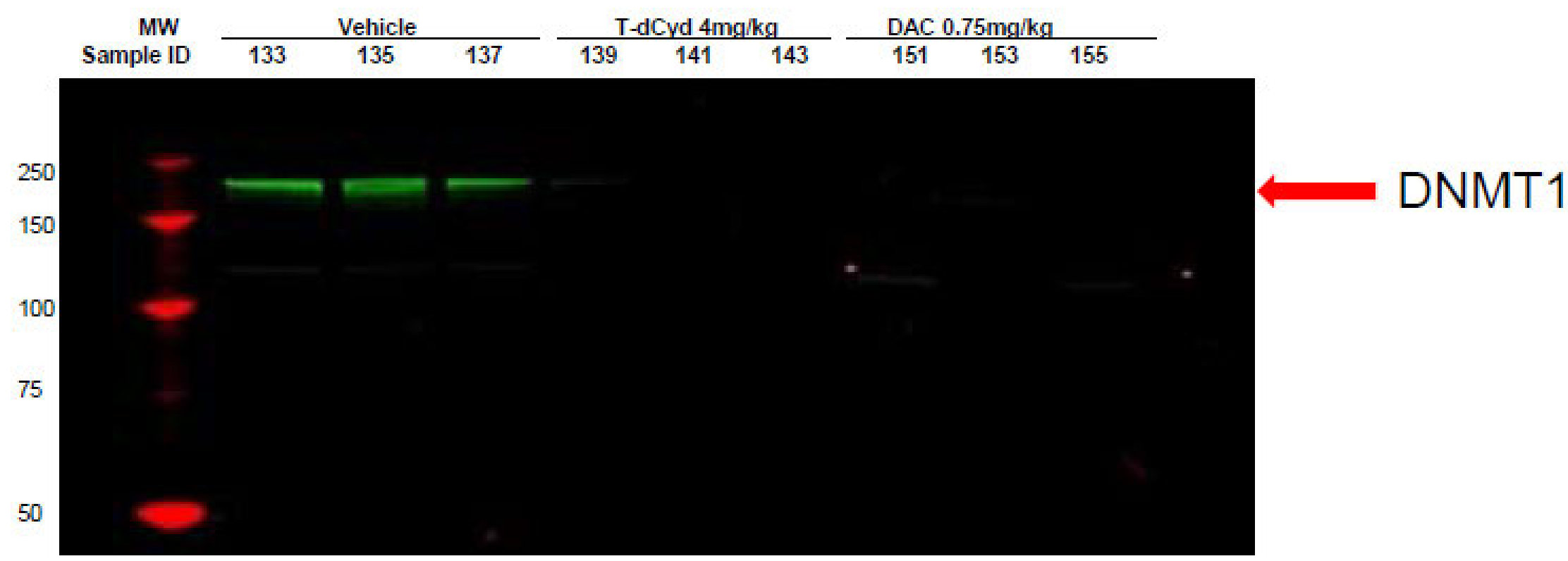

Fig 4: H23 xenograft tumors from mice treated QDx5 for 3 cycles with 4 mg/kg TdCyd, 0.75 mg/kg decitabine, or saline control were collected 6h after the last dose. Tumor protein (50 µg/well) run on a 4-12% Tris Glycine 1.5 mm gel was blotted and stained with rabbit monoclonal anti-DNMT1 (primary) and Li-Cor IRDye 800 anti-rabbit (secondary). DNMT1 levels decreased following TdCyd and decitabine treatment (unpublished data).

Although TdCyd treatment reproducibly inhibited tumor growth in the SK-ES1 Ewing sarcoma model, DNMT1 levels were not changed. DNMT data obtained to date and tabulated below, show a good correlation between DNMT1 reduction and TdCyd growth inhibitory activity with the exception of this SK-ES1 model.

	SK-ES1	NCI-H23	HCT116	DLD1
**Preclinical efficacy?**	growth inhibition	growth inhibition	weak inhibition	no effect
**DNMT1 decrease?**	no change	decrease	no change	no change
**DNMT3A decrease?**	no change	no change	no change	no change

DNMT1 is known to be increased in several different cancers [15]. It is not known whether TdCyd activity in some preclinical models is due to different levels of target DNMT1, but IHC data from DCTD's National Clinical Target Validation Laboratory indicate that DNMT1 is constitutively expressed in tumor cells at intermediate and high levels in 12.5% (7/56) of lung cancers, 7.7% (3/39) of ovarian tumors, and 15% (12/78) of lymphomas. Further studies to explore whether changes in DNMT1 expression can serve as a pharmacodynamic marker for the effects of treatment with TdCyd and other DNMT inhibitors, or whether baseline levels might

predict efficacy for prospective patient selection, are ongoing in a range of responsive and non-responsive tumor models.

Our results also show that levels of another member of the DNA methyltransferase family, DNMT3(A2), were unaffected by TdCyd treatment in any of the studies. This enzyme has been demonstrated to be euchromatin-localized within specific cell populations in normal mouse and man, including embryonic stem cells, ovary, testis, thymus and spleen [16].

2.5 Preclinical Pharmacology and Toxicology

Range-finding toxicology studies and IND-enabling studies were conducted with Sprague-Dawley rats and beagle dogs. The range-finding studies were conducted as non-GLP, but followed good and sound research practices. The objectives of the range-finding studies were to determine the maximum tolerated dose (MTD); to determine target organs of toxicity and any dose limiting toxicities (DLT); and to establish whether route of administration affected toxicity. One further objective was to determine whether pretreatment with tetrahydrouridine (THU, NSC 112907), an inhibitor of cytidine/deoxycytidine deaminase, had any effect on toxicity; this agent is co-administered in the NCI's clinical trials of fluorodeoxycytidine (FdCyd) to prevent FdCyd's rapid degradation by cytidine deaminase, and thus increase the AUC. In these range-finding studies, TdCyd was administered on a daily x 5 schedule for 2 or 4 cycles by i.v. or oral route, with and without THU pretreatment.

Preclinical data also indicated that although THU pretreatment dramatically increased TdCyd exposure (AUCs up to 20-fold greater), co-administration of THU decreased TdCyd's antitumor effects. The decision was therefore made to not include THU in the clinical plan, and thus THU was not included in IND-enabling studies.

IND-enabling studies were conducted in rats and dogs to estimate the safe starting dose for clinical studies, to further characterize toxic effects with respect to target organs (clinical pathology and histopathology), and to evaluate reversibility of drug effects. Limited toxicokinetic evaluations were performed. Because TdCyd was determined to be more efficacious following oral rather than i.p. administration in the preclinical xenograft studies, only the oral route was pursued. TdCyd was administered orally, daily x 14 with a 7-day recovery group in both rat and dog. Systemic exposure to TdCyd was also studied in both species; blood samples were collected for plasma drug analysis.

2.5.1 Range-finding study of oral and intravenous TdCyd in rats (Study 36883)

Oral TdCyd doses were 4 and 8 mg/kg/day (+/- THU); i.v. TdCyd doses were 1 and 4 mg/kg/day (+/- THU). Animals that received THU pretreatment (100 mg/kg 1 hour prior to TdCyd administration) were dosed for 2 cycles followed by 15 non-dosing days prior to necropsy, animals that did not receive THU were dosed for 4 cycles. Blood was collected 2 minutes (i.v. groups) or 30 minutes (oral groups) and 4 hours after TdCyd administration on Day 12 to assess plasma-drug concentrations. Blood was collected at 4 hours post dose administration for clinical pathology assessment on Days 5, 12, and 19 and on the day of scheduled necropsy (Day 27).

All rats administered TdCyd orally survived to scheduled termination. Five rats from the IV groups died early (days 4 through 22) and one was euthanized due to moribund condition from complications from the in-dwelling cannula. Clinical observations noted in i.v.- group rats that survived until scheduled necropsy included: thin (12/15 rats, including 2 vehicle-group rats), hunched (1/15 rats), decreased activity (1 vehicle group rat), rough coat (2/15 rats), and eye and nasal discharge (1/15 rats). Clinical observations noted in oral group were limited to eye abnormalities, which were attributed to blood collection. Microscopic observations included evidence of right atrial thrombosis and thrombosis of one or more main branches of the pulmonary artery, which in combination was considered the cause of morbidity/death of five of these early death animals. The rat that was found dead on Day 4 did not have evidence of thrombosis in the heart or lungs. However, this rat did have hepatic necrosis, as did the other unscheduled-death rats (which in those animals was considered a consequence of thromboembolism) so its death was potentially of related pathogenesis.

There were no treatment-related changes in group mean body weights or body weight gain in any of the animals. There were no treatment related changes in hematology or serum chemistry parameters following i.v. administration. Increases in neutrophil, lymphocyte, and white blood cell counts noted at Day 27 correlated with inflammation noted microscopically.

Right atrial thrombosis was identified in rats that died early (6/7, but only a single animal that survived to study conclusion), pulmonary thrombosis or secondary changes associated with this condition were widely distributed throughout all i.v.-treated animals (including 2 of 3 vehicle group animals) attributable to complications of the indwelling jugular catheter. Oral doses of 4 or 8 mg/kg/day TdCyd with or without THU did not produce any toxicity in this study.

2.5.2 Range-finding study of oral and intravenous TdCyd in dogs (Study 46509)

This study was conducted in two phases. In Phase 1, Dogs received TdCyd (0.67 mg/kg; 13.4 mg/m^2) daily x 5 x 2 for a total of 10 doses by either oral gavage or i.v. infusion (30 min), with or without oral pretreatment of THU (100 mg/kg; administered 1 hr prior to TdCyd dosing). Due to observed toxicity, the final (tenth) dose of TdCyd was omitted and all dogs receiving TdCyd in Phase I were euthanized due to moribund condition by Day 15. Toxicity was similar in all animals regardless of route of administration or pretreatment with THU.

In Phase 2, dogs were administered TdCyd, on the same schedule, but orally only, and at reduced doses (20% and 10% of the Phase I doses) of 0.134 or 0.067 mg/kg (2.7 and 1.34 mg/m^2, respectively). Due to its lack of effect in Phase 1, no dogs in Phase 2 received THU pretreatment.

Endpoints evaluated for both Phases were mortality, clinical observations, body weights, clinical pathology (hematology and serum chemistry) cardiac troponin-I (cTnI), and gross and microscopic pathology (Phase I only). Blood was collected for plasma-drug concentration and PK analysis.

Clinical signs during Phase 1 (0.67 mg/kg/day) included oral blisters and bleeding gums, excessive salivation, skin lesions (particularly around the genitalia), lethargy, thin appearance, anorexia, diarrhea, and penile discharge. Phase I dogs had up to 27% loss in body weight,

correlated with observations of thin appearance and decreased food consumption and were all attributed to the administration of TdCyd.

Changes in hematology parameters attributed to TdCyd administration for Phase 1 dogs were noted on Day 5; 7/8 animals had decreased total lymphocyte counts in comparison to vehicle group dogs. On Day 11, decreases in total lymphocyte counts were noted in all treated dogs regardless of route of administration or pretreatment with THU. Changes in serum chemistry parameters attributed to the administration of TdCyd included increased alkaline phosphatase, globulin, triglycerides, and cholesterol, and decreased albumin and albumin/globulin ratio. There were no changes in Troponin-I concentration attributed to TdCyd.

Histological findings associated with TdCyd administration included epithelial necrosis and ulceration in the oropharyngeal, esophageal, and gastrointestinal mucosa, as well as in the skin. Bone marrow cellularity was reduced in all four treated dogs evaluated microscopically, most noticeably, the two that were dosed by oral gavage. There were no adverse clinical observations or changes in clinical pathology attributed to TdCyd administration in Phase 2.

Based on these findings, the 0.67 mg/kg (13.4 mg/m^2) dose was lethal regardless of route of administration or pretreatment with THU. The target organs of toxicity are the GI mucosa and the bone marrow.

2.5.3 IND-enabling (GLP) study of oral TdCyd in rats (Study 2330-012-001)

The objective of the study was to determine the target organ toxicity of TdCyd administered orally for 14 consecutive days (followed by 7 days recovery) at doses of 0, 0.5, 2.0 and 10 mg/kg/day. Five rats per sex per group were necropsied on Day 15 and remaining rats were necropsied on Day 22. Experimental endpoints included moribundity/mortality; clinical signs of toxicity; body weights; clinical pathology parameters (coagulation, hematology, and clinical chemistry); plasma drug levels/pharmacokinetics; organ weights; gross pathology at necropsy; and microscopic pathology.

No animals in the study died prior to their scheduled necropsy and no treatment-related clinical signs of toxicity were observed. There were significantly decreased body weight gains ($p <$ 0.05 compared to control) in high-dose males during the intervals from Days 5-8, 12-14, and 1-14, and in high-dose females during the interval from Days 12-14. Effects on body weight gain in the high dose groups were reversible, as no significant differences in mean body weight changes were seen after cessation of dosing. No toxicologically significant, treatment-related differences in clinical pathology parameters (clinical chemistry, hematology, or coagulation), or organ weights were seen during the study, and no test article-related gross or microscopic changes were identified.

TdCyd at doses up to 10 mg/kg/day (60 mg/m^2) once daily for 14 consecutive days was well tolerated. With the exception of decreased but reversible body weight gain in the high-dose group, no treatment-related changes were measured. No limiting toxicity was seen in any dose group in the study.

2.5.4 IND-enabling (GLP) study of oral TdCyd in dogs (Study 49204)

The objective of this study was to determine target organ toxicities of TdCyd when administered orally to beagle dogs once daily for 14-consecutive days followed by a 7-day recovery period. Four dogs/sex/group received TdCyd at 0.067, 0.134, and 0.30 mg/kg/day via oral gavage. All surviving dogs were necropsied on Day 15. Endpoints to determine toxicity were clinical observations, body temperatures, body weights, food consumption, cardiac biomarker (Troponin-I) assessment, and clinical and anatomic pathology including organ weights. Blood specimens were collected at selected time points following dose administration on Day 1 and at a single time point following the final dose administration.

Dose administration was suspended following the 12th dose (Day 12) for the 0.30 mg/kg/day group due to adverse clinical signs: one male dog in this group was humanely euthanized due to moribund condition on Day 13. Clinical observations on Day 13 for this dog included: red gums, red penile discharge, diarrhea with red discharge, and a skin abrasion to the genitalia. Clinical observations were generally common to both sexes for a given dose group. Abnormal observations attributed to TdCyd administration were not noted until Day 8. For dogs in the 0.067 mg/kg/day group, observations attributed to TdCyd included discolored (red) gums and ears, penile/vaginal discharge (yellow/green), and changes in fecal status (soft feces). One or more of the dogs in the 0.134 mg/kg/day group presented with the abnormal observations noted in the 0.067 mg/kg/day dose group and also were noted with skin abrasions (limited to males, scrotum, at this dose level) and/or yellow/green eye discharge, also attributed to TdCyd administration. Observations only noted in the 0.30 mg/kg/day group dogs and attributed to TdCyd administration included decreased activity, lethargy, nasal discharge, rough coat, skin ulcers on the fore- and hind-limbs, hunched posture, red penile discharge, and skin abrasions on the head/neck and nose/muzzle. Many of the observations persisted until the day of scheduled necropsy (Day 15 or Day 22).

There were no changes in body temperatures when compared to baseline values or to the control group and no detectable cardiac Troponin-I. While there were no statistically significant differences in group mean body weights compared to the control group, the 0.30 mg/kg/day male group mean body weight on Day 22 was 9 percent lower when compared to the Day 1 mean body weight for this group. This loss was attributed to TdCyd administration. The body weight loss in the 0.30 mg/kg/day group males correlated with lower food consumption values. Group mean feed consumed per day values were lower when compared to the control group beginning on Day 5 for males (noted as statistically lower on Days 11, 12, and 21) and on Day 13 for females (noted as statistically lower on Day 19).

Clinical pathology changes attributed to TdCyd administration noted in the 0.134 and 0.30 mg/kg/day groups on Day 15 did not exhibit recovery by Day 22, or only exhibited partial recovery. Clinical pathology changes in the 0.134 and 0.30 mg/kg/day group males and females included increased fibrinogen; changes in the 0.134 mg/kg/day group females and the 0.30 mg/kg/day group males and females included decreased red cell counts, hemoglobin, hematocrit and reticulocyte counts and increased total white cell counts, neutrophils, monocytes, and large unstained cells, as well as, decreased lymphocytes, total lymphocytes, eosinophils, and basophils. Decreased total protein, albumin, albumin/globulin ratio, and calcium were noted in the 0.30 mg/kg/day group males.

Daily x 14 oral administration of TdCyd at 0.134 and 0.30 mg/kg/day (12 days for the 0.30 mg/kg/day group) led to epidermal necrosis and ulceration on Day 15 that increased in incidence and progressed to involve epidermal hyperplasia and hyperkeratosis at recovery necropsy on Day 22. These keratinocyte-targeted lesions resembled erythema multiforme microscopically. At doses of targeted 0.30 mg/kg/day, necrosis and ulceration were also noted on mucous membranes, persisting in the oral mucosa or tongue in the Day 22 recovery group. While only one 0.30 mg/kg/day male dog, terminated moribund on Day 13, had gastric ulceration, several dogs administered 0.134 and 0.30 mg/kg/day had suppurative inflammation and hemorrhage in the intestinal tract associated with Peyer's patches on Days 15 or 22. Thymic atrophy (involution) was increased in incidence and/or severity in both males and females in the targeted 0.30 mg/kg/day groups on Days 15 and 22. There were no test article-related lesions noted in dogs administered 0.067 mg/kg/day TdCyd.

A no-observed-adverse-effect level (NOAEL) was established for males and females at 0.067 mg/kg/day. There were no changes in body weights, food consumption values, or changes to clinical or anatomic pathology parameters attributed to TdCyd for this group. The clinical observations attributed to the administration of TdCyd noted for the 0.067 mg/kg/day dose group were limited to red gums, changes in fecal status, and yellow/green vaginal/penile discharge. The red gums were noted beginning Day 11, which was later than noted for the 0.134 and 0.30 mg/kg/day groups, and resolved during the 7-day recovery period. Changes in fecal status (soft feces, mucoid feces, and diarrhea) are an expected consequence following administration of chemotherapeutics and, with management of hydration, were not considered adverse. Yellow/green penile/vaginal discharge was limited to two males and one female, and did not have any correlating changes in clinical pathology parameters or microscopic correlates and therefore, were not considered adverse in that dose group.

2.5.5 Toxicology Summary

Based on these results, when TdCyd is administered daily for 14 consecutive days, the dog is the most sensitive species, with a Highest Non-Severely Toxic Dose (HNSTD) of 0.067 mg/kg/day (1.34 mg/m^2/day) and an MTD of >0.134 mg/kg <0.3 mg/kg.

Daily oral administration of TdCyd at 0.134 and 0.30 mg/kg/day led to epidermal necrosis and ulceration on Day 15 that increased in incidence and progressed to involve epidermal hyperplasia and hyperkeratosis at recovery necropsy on Day 22. These keratinocyte-targeted lesions resembled erythema multiforme microscopically. There were no test article-related lesions noted in dogs administered 0.067 mg/kg/day.

To obtain the first-in-human starting dose, we multiplied the HNSTD (in mg/m^2) by 1/5 and then multiplied that product by the average human BSA (1.8 m^2). The TdCyd HNSTD is 0.067 mg/kg (1.34 mg/m^2) x 1/5 = 0.27 mg/m^2. Multiplying 0.27 mg/m^2 by 1.8 yields 0.48 mg, which was rounded to 0.5 mg based on capsule size.

A list of adverse events that occurred during the animal studies is included in Section 7.1.

2.5.6 Pharmacokinetics

TdCyd exposures calculated from blood samples collected on the mouse efficacy studies and from rats and dogs after a single oral or i.v. dose on the NCI-sponsored toxicology studies) are summarized in the table below (similar doses adjusted for body-surface area). The oral formulation of TdCyd shows adequate bioavailability.

Species	Dose		PO AUC (min• µM)	PO Cmax (µM)	IV Cmax (µM)
	mg/kg	mg/m^2			
Mouse	5*	15	9.45	0.45	12.2
Rat	2	12	307.8	7.1	9.5
Dog	0.67**	13.4	25.0	2.2	6.5

*The estimated efficacious dose in mice is 2 to 4 mg/kg; **Exceeds the MTD

Following oral administration, TdCyd systemic availability was low in mice (8.4%) and high in rats (124.7%). In the dog, systemic availability was 60.1% without THU; absorption appeared to be linear over the dose range tested. Due to the rapid metabolism of TdCyd to TdUrd (see below), it is likely that TdCyd bioavailability is considerably higher. In the dog, this is reflected in a 20% increase in bioavailability when animals were pretreated with THU (80%)

In in vivo metabolism studies, 4'-thio-thymidine (TdThd; NSC 776949) and 4'-thio-2'-deoxyuridine (TdUrd; NSC 776952) were the 2 major metabolites identified in mice, rats, and dogs following TdCyd dosing. As shown in Figure 5, plasma levels of TdCyd rose rapidly after a single oral dose, reaching a peak within 60 minutes in all three species. Levels of TdUrd also increased quickly, with the mouse attaining levels almost 40-fold that of TdCyd. In the rat, TdUrd concentrations remained below those of TdCyd throughout the 4 hours following dosing, while in the dog, TdUrd levels rose and peaked similarly to those of TdCyd. However, in contrast to TdCyd, TdUrd concentrations remained above 1 µM for 60 min through 4 hours post dose. Both the rat and dog formed appreciable levels of TdThd, with the dog forming greater than 10x the level seen in mice. There was no detectable TdThd in the rat.

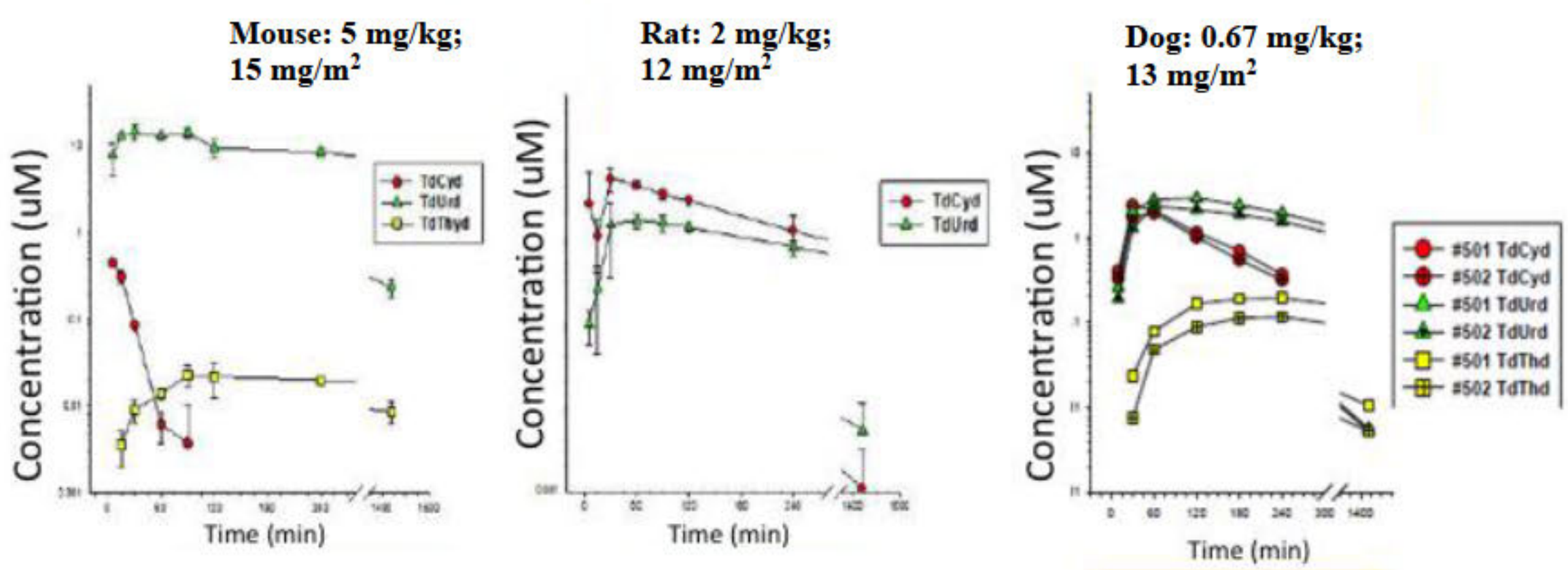

Fig 5: Plasma v. time plots for TdCyd (red) and 2 of its metabolites TdUrd (green) and TdThd (yellow) in mouse, rat, and dog following oral administration of TdCyd at similar doses (adjusted for body surface area).

The relative activity of TdCyd and its 2 major metabolites was assessed in vitro using the NCI-60 panel of human-derived tumor cells. Concentrations for all 3 compounds ranged from 0.01 to 100 micromolar, and the incubation period was 48 hours. Total growth inhibition (TGI) was not reached for any compound in any cell line, but these results do provide a frame of reference for evaluation of circulating concentrations to be measured during the clinical study.

No distribution, excretion, or in vitro metabolism studies have been conducted with TdCyd at this time. As a secondary objective on this study, blood and urine samples will be collected during cycle 1 to measure the pharmacokinetics of TdCyd (see Section 9.1).

2.6 Correlative Studies

Preclinical pharmacodynamic assessment of TdCyd has focused on DNMT1 as a clinically validated drug target in at least some cancers. Although DNMT1 levels have been measured in tumors by Western blot and immunohistochemistry, the optimum method to measure DNMT1 levels in tumor tissue has not yet been determined. For this reason we will not be collecting tumor biopsies during the initial dose escalation phase of this study; an amendment will be submitted to allow collection of biopsies on an expansion cohort once the MTD has been established and a method has been validated. An immunoassay is also in development to measure DNMT1 in CTCs.

Recognizing that inhibition of DNMT1, and in turn DNA methylation, can result in re-expression of tumor suppressor genes (i.e., p16, p15, MGMT, DAPK, TIMP3, RASSF1A), the Pharmacodynamic Assay Development Lab (PADIS) at the Frederick National Laboratory has developed and validated an immunofluorescence p16INK4A expression assay for CTCs using the CellSearch system and a p16/INK4a monoclonal antibody conjugated to Alexa Fluor 488 (p16/INK4a-AF488). Biomarker utility for the p16INK4A assay has been demonstrated in patients on an NCI clinical trial of fluorodeoxycytidine (FdCyd) and tetrahydrouridine (THU) (09-C-00214) (Figure 6).

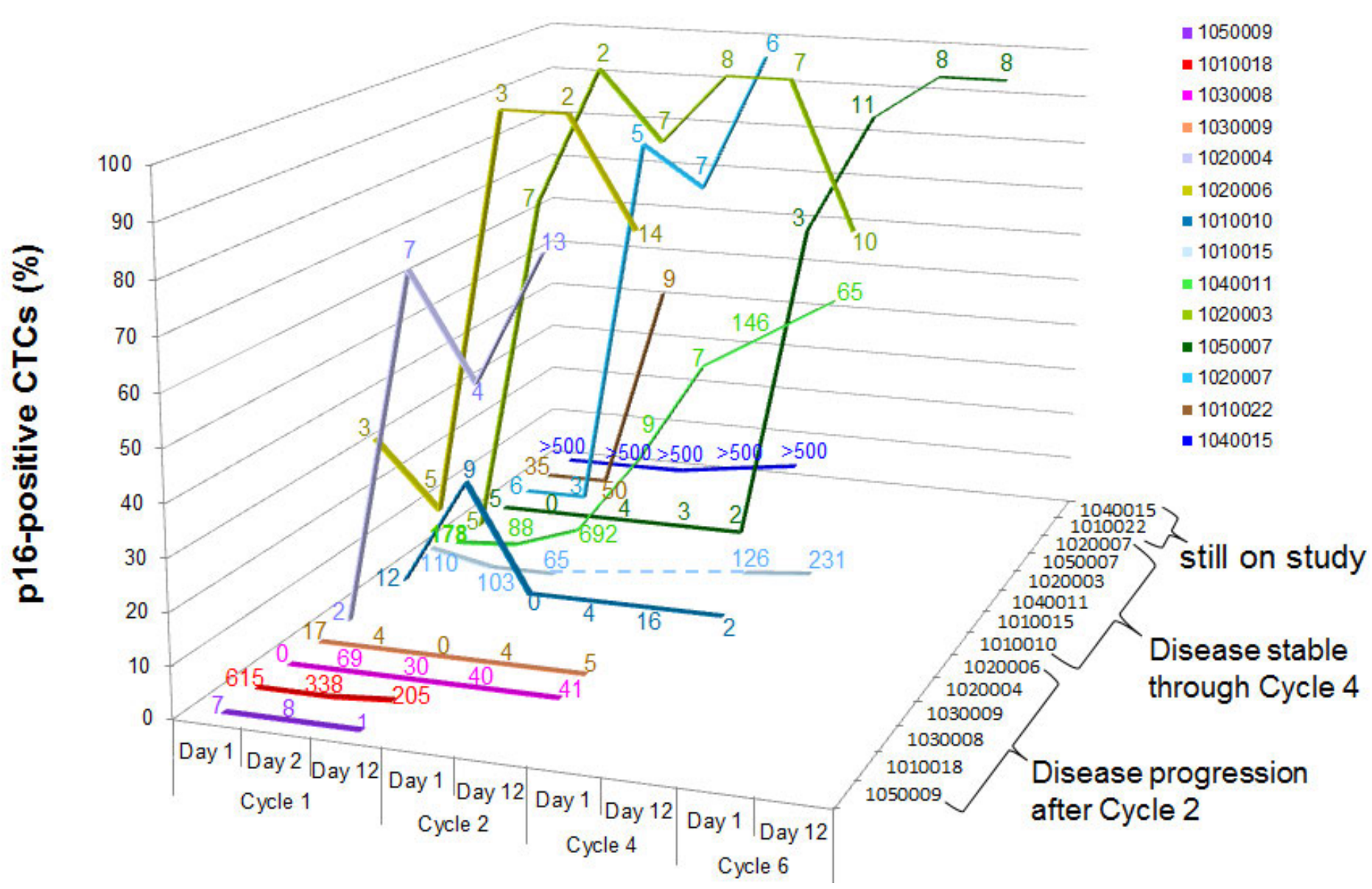

Figure 6: p16Ink4A - positive CTC changes during treatment with FdCyd + THU in evaluable patients. The number labeled on each line is the CTC count obtained from each 7.5 mL specimen.

While TdCyd can induce re-expression of

methylation-inhibited tumor suppressor genes, it can also potentially affect the expression of other genes necessary for normal cellular function. Although no comprehensive survey is technically possible at present, PADIS measured the expression levels of a panel of 32 housekeeping genes following 96-hour TdCyd or decitabine exposure in vitro, and determined that the expression of these housekeeping genes was generally unaffected (data presented at AACR 2014).

CTCs will be isolated from whole blood samples collected throughout the study for assessment of p16/INK4a, TIMP3, and DNMT1 expression. Development and validation of PCR-based assays for the other tumor suppressor genes listed above are in progress.

CTCs will also be collected at baseline and then throughout the study to evaluate whether we can measure changes in the number and phenotype (epithelial-mesenchymal transition) of CTCs in patients over time to explore any correlation with response to treatment or disease progression. This analysis will be performed in Dr. Kinders' lab with the ApoStream instrument, which uses antibody-independent CTC isolation technology that can isolate viable CTCs from epithelial and non-epithelial cancers.

2.7 Clinical Status

Twenty-six patients have been accrued as of January 2019 (Amendment J). One patient on DL7 was diagnosed with grade 3 *Pneumocystis jiroveci* pneumonia (PJP) after his first cycle, and a second patient who had been on DL 3 for 10 cycles and was intrapatient dose-escalated to DL6 was also found to be infected with PJP (grade 3). Both patients had bronchoscopy performed. Both patients had lymphopenia and negative HIV tests, and both patients were treated for PJP with resolution of their pulmonary symptoms. Dose escalation was held and then resumed at DL5A (6 mg) with a traditional 3+3 design with appropriate prophylaxis and monitoring. One patient was accrued to DL5 on the accelerated titration schema, and 5 additional patients received subsequent therapy at DL5A on the revised 3+3 design dose level table (Section 5), with one DLT (transaminitis) observed. No DLTs were observed in the patients enrolled on DL6A and DL7A, and patients are currently being accrued to DL8A. However, a third NCI DTC patient was diagnosed with PJP prior to their enrollment on any DTC study, and another patient enrolled on an unrelated NCI DTC study was also diagnosed with PJP, indicating that PJP incidence may not be related to TdCyd.

As of November 2018, 2 patients (enrolled on DL7A and DL8A, respectively) have undergone hospitalization (during Cycle 4 and Cycle 2, respectively) due to adverse events that are not clearly related to TdCyd and may instead be related to the antifungal prophylaxis being administered to prevent PJP (Bactrim or Pentamidine): itching eyes, lower extremity edema, rhinorrhea, and cough (both patients), as well as borderline pulmonary vascular congestion, mild pulmonary edema, mild peribronchial thickening, and shortness of breath for the patient enrolled on DL7A, and diarrhea, eye soreness, facial rash/hyperpigmentation, and anemia for the patient enrolled on DL8A; these events were managed and resolved for both patients by holding Bactrim or Pentamidine. Because the antifungal prophylaxis may be related to these adverse events and the association of PJP with TdCyd is not clear, antifungal prophylaxis was eliminated as of Amendment I (11/28/18). Instead, patients are being closely monitored for

symptoms of PJP and treated with antifungal agents as clinically indicated. An additional PK sampling time point (cycle 1 day 8) was included to assess occurrence of any TdCyd metabolite accumulation that may contribute to the observed toxicities.

As of January 2019 (Amendment J), DLT monitoring will be extended to cycle 2 due to the death of a patient on dose level 8A following their hospitalization during cycle 2 with grade 5 pneumonia, grade 4 acute renal failure, and grade 4 thrombocytopenia. The pneumonia was postobstructive due to tumor and unlikely to be attributed to TdCyd, with no signs of *Pneumocystis jiroveci* infection. The latter 2 adverse events were likely due to the infection, but the possibility of attribution to TdCyd could not be ruled out. Because of this concern, an additional 3 patients will be enrolled on the previously cleared, next-lowest dose level (7A). We plan to extend our DLT monitoring for MTD determination to 2 cycles (cycle 1 and cycle 2), and additional PK sampling has been added on cycle 2 day 1 to assess any TdCyd metabolite accumulation that may contribute to the toxicities observed during cycle 2.

2.8 Rationale

TdCyd offers an improvement over current DNA methyltransferase inhibitors by virtue of a higher incorporation rate into DNA at lower cytotoxicity levels. TdCyd is anticipated to have improved activity over the nucleoside analog FdCyd without the need for co-administration of the cytidine/deoxycytidine deaminase inhibitor THU. Preclinical data suggest that treatment with TdCyd at doses that are well tolerated in extended schedules will inhibit tumor growth due to DNMT1 depletion. Measurement of tumor suppressor p16 re-expression in patient circulating tumor cells has already been demonstrated on the NCI's clinical trial of FdCyd plus THU, and this approach will be used to measure the pharmacodynamic effect of TdCyd.

3 PATIENT SELECTION

3.1 Eligibility Criteria

3.1.1 Patients must have histologically documented solid tumors whose disease has progressed on standard therapy or for which there is no available standard therapy.

3.1.2 Patients must have measurable or evaluable disease.

3.1.3 Age ≥18 years of age.

3.1.4 ECOG performance status ≤ 2 (see Appendix A).

3.1.5 Life expectancy > 3 months.

3.1.6 Patients must have normal organ and marrow function as defined below:

– absolute neutrophil count	≥1,500/mcL
– platelets	≥100,000/mcL
– total bilirubin	≤1.5 X institutional upper limit of normal
– AST(SGOT)/ALT(SGPT)	≤3 X institutional upper limit of normal

– creatinine	≤1.5X institutional upper limit of normal
	OR
– creatinine clearance	≥60 mL/min/1.73 m^2 for patients with creatinine levels above 1.5X institutional normal

3.1.7 Because nucleoside analogs are known to be teratogenic, women of child-bearing potential and men must agree to use two forms of contraception (hormonal or barrier method of birth control; abstinence; sterilization) prior to study entry, for the duration of study participation, and for 3 months after completing study treatment. Should a woman become pregnant or suspect she is pregnant while she or her partner is participating in this study, she should inform her treating physician immediately. Men treated or enrolled on this protocol must also agree to use two forms of contraception prior to the study, for the duration of study participation, and for 3 months after completion of administration of TdCyd.

3.1.8 Patients must have completed any chemotherapy, radiation therapy, or biologic therapy ≥ 4 weeks or 5 half-lives (whichever is shorter) (6 weeks for nitrosoureas or mitomycin C) prior to entering the study. Patients must be ≥ 2 weeks since any prior administration of a study drug in a phase 0 or equivalent study and be ≥ 2 weeks since any prior palliative radiation or cyberknife therapy. Patients must have recovered to grade 1 from prior toxicity or adverse events. Patients with bone metastases or hypercalcemia on intravenous bisphosphonate treatment prior to study entry may continue this treatment.

3.1.9 Ability to understand and the willingness to sign a written informed consent document.

3.1.10 Willingness to provide blood and urine samples for research purposes.

3.1.11 Ability to swallow pills/capsules.

3.2 Exclusion Criteria

3.2.1 Patients who are receiving any other investigational agents.

3.2.2 Pregnant women and women who are breastfeeding are excluded from this study.

3.2.3 Patients with clinically significant illnesses which would compromise participation in the study, including, but not limited to active or uncontrolled infection, immune deficiencies, known HIV infection requiring protease inhibitor therapy, Hepatitis B, Hepatitis C, uncontrolled diabetes, uncontrolled hypertension, symptomatic congestive heart failure, unstable angina pectoris, myocardial infarction within the past 6 months, uncontrolled cardiac arrhythmia, or psychiatric illness/social situations that would limit compliance with study requirements.

3.2.4 Patients with known brain metastases or carcinomatous meningitis are excluded from this clinical trial, with the exception of patients whose brain metastatic disease status has remained stable for ≥ 1 month after treatment of the brain metastases.

Patients should not be on anti-seizure medications. These patients may be enrolled at the discretion of the Principal Investigator.

3.2.5 Malabsorption syndrome or other conditions that would interfere with intestinal absorption.

3.3 Inclusion of Women and Minorities

Both men and women of all races and ethnic groups are eligible for this trial.

3.4 Research Eligibility Evaluation

3.4.1 A complete history and physical examination (including height, weight, vital signs, and performance score) will be conducted within 1 week prior to enrollment. This will include evaluation of measurable disease, ECG, and determination of performance status.

3.4.2 Diagnostic imaging studies must be performed within 30 days prior to the start of agent administration.

3.4.3 Laboratory Evaluation: Baseline laboratory data are to be obtained within 1 week prior to enrollment:

- Hematological Profile: CBC with differential, platelets.
- Biochemical Profile: electrolytes, BUN, creatinine, glucose, AST, ALT, bilirubin, calcium, phosphorous, albumin, magnesium, alkaline phosphatase, LDH
- Urinalysis
- Serum or urine pregnancy test for female participants of childbearing potential.

4 REGISTRATION PROCEDURES

4.1 Registration Process

Authorized staff must register an eligible candidate with NCI Central Registration Office (CRO) within 24 hours of signing consent. A registration Eligibility Checklist from the Web site (http://home.ccr.cancer.gov/intra/eligibility/welcome.htm) must be completed and sent via encrypted email to: NCI Central Registration Office (HOIS) (ncicentralregistration-l@mail.nih.gov). After confirmation of eligibility at Central Registration Office, CRO staff will call pharmacy to advise them of the acceptance of the patient on the protocol prior to the release of any investigational agents. Verification of Registration will be forwarded electronically via e-mail to the research team.

Off Protocol Therapy and Off-Study Procedure: Authorized staff must notify the Central Registration Office (CRO) when a patient is taken off protocol therapy and when a

patient is taken off-study. A Participant Status Updates Form from the Web site (http://home.ccr.cancer.gov/intra/eligibility/welcome.htm) main page must be completed and sent via encrypted email to: NCI Central Registration Office (HOIS) (ncicentralregistration-l@mail.nih.gov).

5 TREATMENT PLAN

This is an open-label Phase I trial of oral TdCyd in patients with refractory solid tumors whose disease has progressed on standard therapy that is known to be associated with a survival advantage or have disease for which there is no known standard therapy.

Patients will be required to maintain a study diary documenting when the doses were taken, and any associated side effects (Appendix B). Patients will be asked to return the diary to the clinic staff at the end of each cycle.

History and physical exam will be performed at baseline, during weeks 1 and 2 of cycle 1, and at the start of every cycle thereafter (within 3 days prior to treatment).

- Labs (CBC with differential; serum chemistries, platelets) will be performed at baseline, during weeks 1 and 2 of cycle 1, and at the start (+/- 1 day during the cycle and up to 3 days before start of a new cycle) and during week 1 of every cycle thereafter. Lymphocyte subset phenotypical analysis will be performed before each cycle.

ECG will be performed at baseline, on cycle 1 day 8 (+/- 1 day), and as clinically indicated.

Blood and urine samples for correlative PD and PK studies will be collected from all patients as described in Section 9.

CT scans will be performed at baseline; repeat-imaging scans will be performed every 2 cycles for disease restaging (every 3 cycles for patients on study for more than one year, 4 cycles for those on study more than 3 years).

This study will follow design 4 of the Simon accelerated titration designs [18], with dose escalation proceeding in 1-patient cohorts. We will accrue to the next dose level only after we have established safety at lower dose levels. This accelerated titration will stop due to DLT (defined in Section 5.2), and the study will now (with Amendment F) follow a traditional 3+3 design, with the next patient being enrolled at DL5A and the Table for 3+3 Design followed (see Section 5.2). Patients will be closely monitored for side effects and tolerability of the regimen. Dose escalation will continue until the MTD is established.

The starting dose will be 0.5 mg for 5 days a week for 2 weeks, with one week off, in 21-day cycles. Intra-patient dose escalation will be permitted if: **a)** there is no toxicity > Grade 1 that is related (possibly, probably, or definitely) to the study drug at the initial dose level experienced by that patient, **b)** higher doses have been evaluated and completed without DLT,

and **c)** the patient's disease has not progressed. Doses may be escalated, provided conditions a–c are met, up to the last evaluated dose level NOT associated with DLT.

Table for Accelerated Titration Design

Dose Level	TdCyd
-1	0.5 mg for 5 days a week for *one* week, q21-day cycle
**1**	0.5 mg
**2**	1 mg
**3**	2 mg
**4**	3 mg
**5**	6 mg
**6**	12 mg
**7**	20 mg

Table for 3+3 design (after Amendment F)

Dose Level	TdCyd
**-2 (3A)**	2 mg
**-1 (4A)**	4 mg
**5A**	6 mg
**6A**	10 mg
**7A**	14 mg
**8A**	20 mg
**9A**	30 mg
**10A**	40 mg
**11A**	60 mg

5.1 Agent Administration

5.1.1 TdCyd Administration

Oral TdCyd will be administered daily for 5 days a week for 2 weeks, with one week off, in 21-day cycles,

Reported adverse events and potential risks are described in Section 7. Appropriate dose modifications are described in Section 6. No investigational or commercial agents or therapies other than those described below may be administered with the intent to treat the patient's malignancy.

5.2 Definition of Dose-Limiting Toxicity

As of Amendment J (1/16/19), determination of dose-limiting toxicity (DLT) will be based on toxicities observed in the first and second cycles of therapy and felt to be at least possibly related to study drug. Prior to Amendment J, DLT determination was based on toxicities observed during the first cycle of therapy only.

This study will follow an accelerated titration design; dose escalation rules are based on definitions of DLT and of Grade 2 toxicity. Dose levels will increase as specified in the

Dose Escalation Table until DLT or two instances of Grade 2 toxicity are observed at the same dose level during cycle 1 of treatment.

During the accelerated phase, there will be one patient per dose level. When the first instance of Grade 2 toxicity is observed (during the first cycle), two additional patients must be treated at that dose, without experiencing Grade ≥2 toxicity during the first cycle, in order for the accelerated phase to continue. The accelerated phase will end when one patient experiences DLT during the first cycle of treatment, or when two different patients at the same dose level experience Grade 2 toxicity during cycle 1 of treatment. All decisions on dose expansion and DLT will be made on the first cycle of treatment. The accelerated phase ended with Amendment F (1/4/17).

When the accelerated phase ends, the trial will go back to a traditional 3+3 design. That is, additional patients will be placed on the dose level at which the last new patient was treated.

In the standard phase of dose escalation, 3 patients will be treated initially at each new dose level. As of Amendment J (1/16/19), dose escalation will be based on DLTs observed during the first 2 cycles of treatment, and will proceed within each cohort according to the following scheme:

Number of Patients with DLT at a Given Dose Level	Escalation Decision Rule
0 out of 3	Enter 3 patients at the next dose level.
≥2	Dose escalation will be stopped. This dose level will be declared the maximally administered dose (highest dose administered). Three (3) additional patients will be entered at the next lowest dose level if only 3 patients were treated previously at that dose.
1 out of 3	Enter at least 3 more patients at this dose level.<br>• If 0 of these 3 patients experience DLT, proceed to the next dose level.<br>• If 1 or more of this group suffer DLT, then dose escalation is stopped, and this dose is declared the maximally administered dose. Three (3) additional patients will be entered at the next lowest dose level if only 3 patients were treated previously at that dose.
≤1 out of 6 at highest dose level below the maximally administered dose	This is generally the recommended phase 2 dose. At least 6 patients must be entered at the recommended phase 2 dose.

If ≥2 patients experience DLT on dose level -1, the study will be placed on hold and all data reviewed to make a decision about further evaluation of the study drug.

DLT is defined as an adverse event that is related (possibly, probably, or definitely) to administration of study drugs and fulfills one of the following criteria:

5.2.1 Inability to administer ≥ 75% of doses of study drug.

5.2.2 Bullous dermatitis, skin ulceration, or erythema multiforme involving > 5% of the BSA, grade 2 erythroderma, or any grade Stevens-Johnson Syndrome.

5.2.3 Grade ≥ 3 Non-Hematological Toxicity

Grade ≥ 3 non-hematological toxicity felt to be related to study medications will be considered dose-limiting with the following clarifications:

5.2.3.1 Diarrhea Grade 3 will only be considered dose-limiting if it is refractory to treatment as outlined in Section 5.3.2, and unable to be corrected to Grade 2 or less within 24 hours. Bloody or Grade 4 diarrhea will be dose-limiting.

5.2.3.2 Nausea and vomiting Grade 3 will only be considered dose-limiting if it is refractory to anti-emetic therapy and unable to be corrected to Grade 1 or less within 24 hours as outlined in Section 5.3.1.

5.2.3.3 Rise in creatinine to Grade 3, not corrected to Grade 1 or less within 24 hours with IV fluids will be considered dose-limiting. All Grade 4 rises in creatinine will be dose limiting.

5.2.3.4 Grade 3 electrolyte abnormalities will only be considered dose limiting if they are unable to be corrected to grade 2 or less within 24 hours.

5.2.4 Grade 3 thrombocytopenia with bleeding.

5.2.5 Grade 4 thrombocytopenia.

5.2.6 Grade 4 neutropenia ≥ 5 days or febrile neutropenia.

5.2.7 Any degree of anemia, or leukopenia in the absence of grade 4 neutropenia ≥ 5 days, will not be considered dose limiting.

5.2.8 Grade 4 lymphopenia ≥ 14 days will be considered dose limiting; intrapatient dose escalation will also be stopped.

5.2.9 Alopecia will not be considered dose limiting.

5.3 General Concomitant Medication and Supportive Care Guidelines

The predominant toxicities observed in non-clinical toxicology studies were epithelial necrosis and GI inflammation. All patients will be provided with the best available

supportive care. All concurrent medications should be documented prior to initiation of treatment, and be periodically reviewed with the patient.

5.3.1 Nausea/Vomiting

Anti-emetics will not be administered routinely prior to TdCyd. However, if a patient develops nausea/vomiting, anti-emetics such as but not limited to prochlorperazine, metoclopramide, 5-HT3 antagonists, or aprepitant may be given. In addition, if a patient develops nausea and/or vomiting that is Grade 2 or greater, anti-emetics may be instituted prophylactically at the discretion of the investigator. Nausea and vomiting will be considered refractory if it does not resolve to grade 1 or less with a combination of at least 2 anti-emetics within 24 hours.

5.3.2 Diarrhea

Subjects will not be given anti-diarrhea medication prophylactically. If diarrhea develops and does not have an identifiable cause other than study drug administration, addition of anti-motility agents such as tincture of opium (deodorized 10%), Lomotil (diphenoxylate HCl 2.5 mg + atropine sulfate 0.025 mg/tablet) dosed according to the package insert, or loperamide 4 mg po after the first unformed stool with 2 mg po with every 2 hours as long as unformed stools continue (4 mg every 4 hours while asleep) may be used. No more than 16 mg of loperamide should be taken during a 24-hour period). This regimen can be repeated for each diarrheal episode and will continue for 12 hours after the first formed stool. Diarrhea will be considered refractory if it does not resolve within 24 hours ≤ to Grade 2 with the above measures. If the patient develops blood or mucus in the stool, dehydration, or hemodynamic instability, or fever along with the diarrhea, anti-diarrheals will be discontinued and the patient will be treated with IV fluids and antibiotics as medically indicated. Other potentially helpful treatments may also be administered, such as somatostatin analogues, propantheline bromide, etc.

5.3.3 Electrolyte Abnormalities

If hypokalemia, hypophosphatemia, or hypomagnesemia occur, the patient may receive oral or IV supplementation to correct the abnormality. If hyponatremia occurs, the patient may receive 0.9% sodium chloride intravenously to correct the abnormality.

5.3.4 Neutropenia

Febrile neutropenia is a life-threatening complication requiring hospitalization and urgent broad-spectrum antibiotics, as well as an aggressive search for the source and microbial cause of the episode. If clinically indicated, filgastrim will be administered according to accepted guidelines (ASCO or Clinical Center). Study medication will not be reinitiated until at least 24 hours after filgastrim administration.

5.3.5 Anemia

Symptomatic anemia should be treated with red blood cell transfusion and is recommended if the hemoglobin falls below 8 g/dL. The initiation of erythropoietic therapy for the management of chemotherapy-induced anemia follows the American Society of Hematology/ASCO clinical practice guidelines (http://www.asco.org).

5.3.6 Thrombocytopenia

Thrombocytopenia will be treated conservatively. In the absence of bleeding, or a necessary invasive procedure, platelet transfusions should be given for a platelet count ≤10,000/mm^3. If invasive procedure(s) is (are) planned, or the patient develops bleeding, platelet transfusions should be administered in accordance with the standard of practice, usually maintaining a platelet count above 50,000/mm^3.

5.3.7 Dermatologic toxicities

For patients experiencing bullous dermatitis, skin ulceration, or erythema multiforme involving > 5% of the BSA, study drug will be held until resolution. If lesions do not improve by withholding the agent, a dermatology consult should be obtained.

5.3.8 Infection

With Amendment I (11/28/18), *Pneumocystis jiroveci* treatment, such as TMP/SMX DS 1 tablet 3x/week, inhaled Pentamidine, or other appropriate treatment, will be administered as clinically indicated. Beta-(1-3)-D glucan has been noted to be highly sensitive and specific for PJP [17]; this will be checked as clinically indicated. Lymphocyte subset phenotypical analysis will also be performed as clinically indicated. As of Amendment F (v1/4/17) and continuing until Amendment I (v11/28/18), PJP treatment was given prophylactically, starting with cycle 1 and continuing throughout treatment (see Section 2.7).

5.4 Duration of Therapy

In the absence of treatment delays due to adverse events, treatment may continue until one of the following criteria applies:

- Disease progression,
- Intercurrent illness that prevents further administration of treatment,
- Significant toxicity occurs despite 2 dose reductions or no lower dose level exists, or recurrent dermatologic toxicity after one dose reduction as noted in Section 5.3.7
- Stevens-Johnson Syndrome or other life-threatening skin toxicity
- Pregnancy
- Patient decides to withdraw from the study, or
- General or specific changes in the patient's condition render the patient unacceptable for further treatment in opinion of the PI.

5.5 Duration of Follow Up

Patients will be followed for 30 days after the last dose is administered or until one of the following occurs: patient enrolls on another protocol, patient receives standard of care, or death, whichever comes first. The follow-up will consist of a phone call between Days 27-30 after the last dose to evaluate adverse events that were ongoing and any new events that might be deemed related to the therapy. Unacceptable toxicities (i.e., adverse events related to the intervention) that have not resolved by day 30 post-treatment will be followed via biweekly phone calls until stabilization or resolution.

5.6 Criteria for Removal from Study

Patients will be removed from study for one of the following reasons: completed 30-day follow up period or toxicities are unresolved but stabilized, patient enrolls on another protocol, or patient receives standard of care. The reason for study removal and the date the patient was removed must be documented in the medical record and communicated to Central Registration per Section 4.

6 DOSING DELAYS/DOSE MODIFICATIONS

Doses of TdCyd will be held or modified only for adverse events that are felt to be at least possibly related to study drug. Treatment may be delayed for a maximum of 2 weeks for resolution of toxicities; in case toxicities do not resolve as defined below, the patient will not receive further therapy on this protocol and will be followed for resolution of toxicities. Dose modifications are intended for within-cycle and start of next cycle changes. If administration of TdCyd is interrupted for any reason, it will not be made up, and counting of the cycle days will continue.

Patients will be allowed up to 2 dose reductions. For patients on dose level 1, the dose will be reduced to dose level -1 (0.5 mg a day for one week only, q 21-day cycles). Patients on DL5A who require dose reduction will receive DL4A (4 mg) and then DL3A (2 mg). Patients who require a dose reduction will not have the dose re-escalated.

Treatment may be delayed for a maximum of ± 1 day during a cycle due to unavoidable scheduling conflicts.

6.1 Dose Modifications

6.1.1 Drug-related bullous dermatitis, skin ulceration, or erythema multiforme involving > 5% of BSA: TdCyd will be held until resolution. The patient will resume drug at the next lower dose level. Treatment will be discontinued and the patient taken off study if the patient does not improve from a previous episode within 2 weeks, or has a second occurrence. Patients will be taken off study if they develop Stevens-Johnson Syndrome or life- threatening skin toxicity.

6.1.2 Grade 2 drug-related toxicity: Therapy will not be interrupted for Grade 2 hematologic toxicities.

6.1.3 Grade 2 drug-related erythroderma, skin rash, or mucositis: TdCyd will be held for up to 2 weeks until resolution to less than grade 2 and will be restarted at the next lower dose level. Treatment will be discontinued and the patient taken off study if the patient does not improve from a previous episode within 2 weeks, or has a second occurrence.

6.1.4 Grade 3-4 drug-related non-hematologic toxicities: TdCyd will be held until toxicities recover to ≤ grade 1 or baseline (with the exception of alopecia or as noted in Section 6.1.1) prior to re-initiating therapy at the next lower dose level. Electrolyte abnormalities will not require dose reduction if resolution to grade 1 or less is documented within 48 hours. Dose modifications for nausea, vomiting, and diarrhea will be made only if they are refractory to treatment (refer to Section 5.3).

6.1.5 Grade 3 hematologic toxicities: Drug will be held for grade 3 hematologic toxicities, except leucopenia in the absence of neutropenia, and grade 3 lymphopenia, till resolution of toxicity to at least a grade 1 or baseline. Dose modification for grade 3 hematologic toxicity will be made only if the patient has recurrent grade 3 hematologic toxicity despite appropriate supportive care, e.g., growth factors; those experiencing recurrent grade 3 hematological toxicity despite optimal management will be dose reduced to the lower dose level.

6.1.6 The patient will be removed from the study for life-threatening drug-related skin toxicity or Stevens-Johnson Syndrome. No further doses will be administered.

6.1.7 Grade 4 drug-related hematologic toxicities of thrombocytopenia and neutropenia: TdCyd will be held until toxicities recover to ≤ grade 2 and therapy re-initiated at the next lower dose level. TdCyd will not be held or dose modified for leucopenia in the absence of neutropenia, and lymphopenia.

6.1.8 All patients with grade 4 cardiac, respiratory, or neurologic toxicities at least possibly attributable to the investigational drug will be taken off study.

7 ADVERSE EVENTS: LIST AND REPORTING REQUIREMENTS

Adverse event (AE) monitoring and reporting is a routine part of every clinical trial. The following list of AEs will determine whether the event requires expedited reporting (via CTEP-AERS) **in addition** to routine reporting.

7.1 Comprehensive Adverse Events and Potential Risks

The Comprehensive Adverse Event and Potential Risks (CAEPR) list provides a single, complete list of reported and/or potential AEs associated with an agent, using a uniform presentation of events by body system.

7.1.1 Human data: There is no human experience with TdCyd.

7.1.2 Animal data: The starting dose of TdCyd (0.5 mg) was based on 1/5th the TdCyd HNSTD in the most sensitive species, the dog.

There is no ASAEL or CAEPR list for TdCyd. Listed below are toxicities have been observed in animal studies with this agent:

Blood/bone marrow: Anemia; Decreased erythrocytes, reticulocytes, eosinophils, lymphocytes, basophils; Increased neutrophils, monocytes
Constitutional symptoms: Fatigue; Lethargy
Gastrointestinal: Anorexia; Diarrhea; Nausea; Emesis; Mucositis; Vomiting
Metabolic: Weight loss
Dermatologic: Erythema multiforme

7.2 Safety Reporting Requirements/Data and Safety Monitoring Plan

7.2.1 Adverse Event Characteristics

CTCAE term (AE description) and grade: The descriptions and grading scales found in the revised NCI Common Terminology Criteria for Adverse Events (CTCAE) version 4.0 will be utilized for AE reporting until March 31, 2018. CTCAE version 5.0 will be utilized for AE reporting beginning April 1, 2018. All appropriate treatment areas should have access to a copy of the CTCAE version 5.0. A copy of the CTCAE version 5.0 can be downloaded from the CTEP Web site http://ctep.cancer.gov/protocolDevelopment/electronic_applications/ctc.htm.

Attribution of the AE:

1. Definite – The AE *is clearly related* to the study treatment.
2. Probable – The AE *is likely related* to the study treatment.
3. Possible – The AE *may be related* to the study treatment.
4. Unlikely – The AE *is doubtfully related* to the study treatment.
5. Unrelated – The AE *is clearly NOT related* to the study treatment.

7.3 Expedited Adverse Event Reporting

7.3.1 Expedited AE reporting for this study must use CTEP-AERS (CTEP Adverse Event Expedited Reporting System), accessed via the CTEP Web site (http://ctep.cancer.gov). The reporting procedures to be followed are presented in the “NCI Guidelines for Investigators: Adverse Event Reporting Requirements for DCTD (CTEP and CIP) and DCP INDs and IDEs” which can be downloaded from the CTEP Web site (http://ctep.cancer.gov). These requirements are briefly outlined in the tables below (Section 7.3.2).

In the rare occurrence when Internet connectivity is lost, a 24-hour notification is to be made to CTEP by telephone at 301-897-7497. Once Internet connectivity is restored, the 24-hour notification phoned in must be entered electronically into CTEP-AERS by the original submitter at the site.

7.3.2 Expedited Reporting Guidelines

Use the NCI protocol number and the protocol-specific patient ID assigned during trial registration on all reports.

Note: A death on study requires both routine and expedited reporting regardless of causality, unless as noted below. Attribution to treatment or other cause must be provided.

Death due to progressive disease should be reported as Grade 5 "Disease Progression" in the system organ class (SOC) General disorders and administration site conditions." Evidence that the death was a manifestation of underlying disease (e.g., radiological changes suggesting tumor growth or progression: clinical deterioration associated with a disease process) should be submitted.

Phase 1 and Early Phase 2 Studies: Expedited Reporting Requirements for Adverse Events that Occur on Studies under an IND/IDE within 30 Days of the Last Administration of the Investigational Agent/Intervention [1, 2]

FDA REPORTING REQUIREMENTS FOR SERIOUS ADVERSE EVENTS (21 CFR Part 312)

NOTE: Investigators **MUST** immediately report to the sponsor (NCI) **ANY** Serious Adverse Events, whether or not they are considered related to the investigational agent(s)/intervention (21 CFR 312.64)

An adverse event is considered serious if it results in **ANY** of the following outcomes:

1) Death
2) A life-threatening adverse event
3) An adverse event that results in inpatient hospitalization or prolongation of existing hospitalization for ≥ 24 hours
4) A persistent or significant incapacity or substantial disruption of the ability to conduct normal life functions
5) A congenital anomaly/birth defect.
6) Important Medical Events (IME) that may not result in death, be life threatening, or require hospitalization may be considered serious when, based upon medical judgment, they may jeopardize the patient or subject and may require medical or surgical intervention to prevent one of the outcomes listed in this definition. (FDA, 21 CFR 312.32; ICH E2A and ICH E6).

ALL SERIOUS adverse events that meet the above criteria MUST be immediately reported to the NCI via CTEP-AERS within the timeframes detailed in the table below.

Hospitalization	Grade 1 and Grade 2 Timeframes	Grade 3-5 Timeframes
Resulting in Hospitalization ≥ 24 hrs	10 Calendar Days	24-Hour 5 Calendar Days
Not resulting in Hospitalization ≥ 24 hrs	Not required	

NOTE: Protocol specific exceptions to expedited reporting of serious adverse events are found in the Specific Protocol Exceptions to Expedited Reporting (SPEER) portion of the CAEPR.

Expedited AE reporting timelines are defined as:

- "24-Hour; 5 Calendar Days" – The AE must initially be reported via CTEP-AERS within 24 hours of learning of the AE, followed by a complete expedited report within 5 calendar days of the initial 24-hour report.
- "10 Calendar Days" – A complete expedited report on the AE must be submitted within 10 calendar days of learning of the AE.

[1]Serious adverse events that occur more than 30 days after the last administration of investigational agent/intervention and have an attribution of possible, probable, or definite require reporting as follows:
Expedited 24-hour notification followed by complete report within 5 calendar days for:
- All Grade 3, 4, and Grade 5 AEs

Expedited 10 calendar day reports for:
- Grade 2 AEs resulting in hospitalization or prolongation of hospitalization

[2]For studies using PET or SPECT IND agents, the AE reporting period is limited to 10 radioactive half-lives, rounded UP to the nearest whole day, after the agent/intervention was last administered. Footnote "1" above applies after this reporting period.

Effective Date: May 5, 2011

7.3.3 Protocol-specific expedited AE reporting exclusions

Lymphopenia (any grade), alopecia (any grade), anemia (grade 2), electrolytes (grade 2: sodium, potassium, phosphorous, and magnesium), albumin (grade 2), hyperuricemia (grade 3), INR (grade 2), and PTT (grade 2) will not be reported via CTEP-AERS but will be included in the routine data submissions.

7.3.4 Pregnancy, Fetal Death, and Death Neonatal

NOTE: When submitting CTEP-AERS reports for "Pregnancy", "Pregnancy loss", or "Neonatal loss", the Pregnancy Information Form should also be completed for patients who became pregnant on study, and faxed along with any additional medical information to 301-230-0159. The potential risk of exposure of the fetus to the investigational agent(s) or chemotherapy agent(s) should be documented in the "Description of Event" section of the CTEP-AERS report.

7.3.4.1 **Pregnancy**

- Because patients who become pregnant on study risk intrauterine exposure of the fetus to agents which are teratogenic, DCTD/DCP is requesting that pregnancy should be reported in an expedited manner via CTEP-AERS as Grade 3 "*Pregnancy, puerperium and perinatal conditions - Other (pregnancy)*" under the *Pregnancy, puerperium and perinatal conditions* SOC.
- The pregnancy outcome for patients on study should be reported via CTEP-AERS at the time the outcome becomes known, accompanied by the same Pregnancy Report Form used for the initial report.

7.3.4.2 **Pregnancy loss**

- Pregnancy loss is defined in CTCAE as "Death in utero."Any pregnancy loss should be reported expeditiously, as Grade 4 "Pregnancy loss" under the Pregnancy, puerperium and perinatal conditions SOC.
- A pregnancy loss should NOT be reported as a Grade 5 event under the Pregnancy, puerperium and perinatal conditions SOC, as currently CTEP-AERS recognizes this event as a patient death.

7.3.4.3 **Death Neonatal**

- Neonatal death, defined in CTCAE as "A disorder characterized by cessation of life occurring during the first 28 days of life" that is felt by the investigator to be at least possibly due to the investigational agent/intervention, should be reported expeditiously.
- A neonatal death should be reported expeditiously as Grade 4 "Death neonatal" under the General disorders and administration SOC.
- Neonatal death should NOT be reported as Grade 5 "Death neonatal" under the General disorders and administration SOC. If reported as such, the CTEP-AERS interprets this as a death of the patient being treated.

7.3.5 NCI-IRB Expedited Reporting of Adverse Events, Unanticipated Problems, and Deaths

7.3.5.1 **Definitions**

Adverse event

An adverse event is defined as any reaction, side effect, or untoward event that occurs during the course of the clinical trial associated with the use of a drug in humans, whether or not the event is considered related to the treatment or clinically significant. For this study, AEs will include events reported by the patient, as well as clinically significant abnormal findings on physical examination or laboratory evaluation. A new illness, symptom, sign or clinically significant laboratory abnormality or worsening of a pre-existing condition or abnormality is considered an AE. All AEs must be recorded on the AE case report form unless otherwise noted above.

All AEs, including clinically significant abnormal findings on laboratory evaluations, regardless of severity, will be followed until satisfactory resolution. AEs should be reported up to 30 days following the last dose of study drug.

An abnormal laboratory value will be considered an AE if the laboratory abnormality is characterized by any of the following:

- Results in discontinuation from the study
- Is associated with clinical signs or symptoms
- Requires treatment or any other therapeutic intervention
- Is associated with death or another serious adverse event, including hospitalization
- Is judged by the Investigator to be of significant clinical impact
- If any abnormal laboratory result is considered clinically significant, the investigator will provide details about the action taken with respect to the test drug and about the patient's outcome.

Suspected adverse reaction

Suspected adverse reaction means any adverse event for which there is a reasonable possibility that the drug caused the adverse event. For the purposes of IND safety reporting, 'reasonable possibility' means there is evidence to suggest a causal relationship between the drug and the adverse event. A suspected adverse reaction implies a lesser degree of certainty about causality than adverse reaction, which means any adverse event caused by a drug.

Unexpected adverse reaction
An adverse event or suspected adverse reaction is considered "unexpected" if it is not listed in the investigator brochure or is not listed at the specificity or severity that has been observed; or, if an investigator brochure is not required or available, is not consistent with the risk information described in the general investigational plan or elsewhere in the current application. "Unexpected" also refers to adverse events or suspected adverse reactions that are mentioned in the investigator brochure as occurring with a class of drugs or as anticipated from the pharmacological properties of the drug, but are not specifically mentioned as occurring with the particular drug under investigation.

Serious
An Unanticipated Problem or Protocol Deviation is serious if it meets the definition of a Serious Adverse Event or if it compromises the safety, welfare or rights of subjects or others.

Serious Adverse Event
An adverse event or suspected adverse reaction is considered serious if in the view of the investigator or the sponsor, it results in any of the following:

- Death,
- A life-threatening adverse drug experience
- Inpatient hospitalization or prolongation of existing hospitalization
- Persistent or significant incapacity or substantial disruption of the ability to conduct normal life functions
- A congenital anomaly/birth defect.
- Important medical events that may not result in death, be life-threatening, or require hospitalization may be considered a serious adverse drug experience when, based upon appropriate medical judgment, they may jeopardize the patient or subject and may require medical or surgical intervention to prevent one of the outcomes listed in this definition.

Disability
A substantial disruption of a person's ability to conduct normal life functions.

Life-threatening adverse drug experience
Any adverse event or suspected adverse reaction that places the patient or subject,

in the view of the investigator or sponsor, at immediate risk of death from the reaction as it occurred, i.e., it does not include a reaction that had it occurred in a more severe form, might have caused death.

Protocol Deviation (NIH Definition)
Any change, divergence, or departure from the IRB-approved research protocol.

Non-compliance (NIH Definition)
Failure to comply with applicable NIH Human Research Protections Program (HRPP) policies, IRB requirements, or regulatory requirements for the protection of human research subjects.

Unanticipated Problem
Any incident, experience, or outcome that:

- Is unexpected in terms of nature, severity, or frequency in relation to
  (a) the research risks that are described in the IRB-approved research protocol and informed consent document; Investigator's Brochure or other study documents, and
  (b) the characteristics of the subject population being studied; A**ND**
- Is related or possibly related to participation in the research; **AND**
- Suggests that the research places subjects or others at a g*reater risk of harm* (including physical, psychological, economic, or social harm) than was previously known or recognized.

7.4 NCI-IRB and Clinical Director Reporting

7.4.1 NCI-IRB Expedited Reporting of Unanticipated Problems and Deaths

The Protocol PI will report in the NIH Problem Form to the NCI-IRB and NCI Clinical Director:

- All deaths, except deaths due to progressive disease
- All Protocol Deviations
- All Unanticipated Problems
- All non-compliance

Reports must be received by the NCI-IRB within 7 days of PI awareness via iRIS.

7.4.2 NCI-IRB Requirements for PI Reporting at Continuing Review

System Organ Class	CTCAE Term	Grade	# of Events since last CR	Total # of Events	Attribution to Research	Serious?	Unexpected?

The protocol PI will report to the NCI-IRB:

1. A summary of all protocol deviations in a tabular format to include the date the deviation occurred, a brief description of the deviation and any corrective action.

2. A summary of any instances of non-compliance
3. A tabular summary of the following adverse events:
    - All Grade 2 **unexpected** events that are possibly, probably or definitely related to the research;
    - All Grade 3 and 4 events that are possibly, probably or definitely related to the research;
    - All Grade 5 events regardless of attribution;
    - All Serious Events regardless of attribution.

7.4.3 NCI-IRB Reporting of IND Safety Reports

Only IND Safety Reports that meet the definition of an unanticipated problem will need to be reported to the NCI IRB.

7.5 Secondary Malignancy

A *secondary malignancy* is a cancer caused by treatment for a previous malignancy (e.g., treatment with investigational agent/intervention, radiation, or chemotherapy). A secondary malignancy is not considered a metastasis of the initial neoplasm.

CTEP requires all secondary malignancies that occur following treatment with an agent under an NCI IND/IDE be reported via CTEP-AERS. Three options are available to describe the event:

- Leukemia secondary to oncology chemotherapy (e.g., acute myelocytic leukemia [AML])
- Myelodysplastic syndrome (MDS)
- Treatment-related secondary malignancy

Any malignancy possibly related to cancer treatment (including AML/MDS) should also be reported via the routine reporting mechanisms outlined in each protocol.

7.6 Second Malignancy

A second malignancy is one unrelated to the treatment of a prior malignancy (and is **NOT** a metastasis from the initial malignancy). Second malignancies require **ONLY** routine reporting via CDUS unless otherwise specified.

8 PHARMACEUTICAL INFORMATION

A list of the adverse events and potential risks associated with the investigational administered in this study can be found in Section 7.

8.1 4’-thio-2’-deoxycitidine (NSC 764276)

Chemical Name: 4'-thio-2'-deoxycytidine

Other Names: T-dCyd

Classification: DNA methyltransferase I inhibitor

Molecular Formula: $C_9H_{13}N_3O_3S$ **M.W.:** 243.1

Mode of Action: Incorporates into DNA and inhibits DNA methylation resulting in a reduction of tumor growth.

Description: 4'-thio-2'-deoxycytidine drug substance is a white powder.

How Supplied: 4'-thio-2'-deoxycytidine is supplied by the DCTD, NCI and distributed by the Pharmaceutical Management Branch, CTEP, DCTD, NCI as 2 mg and 10 mg capsules.

- 2 mg capsules are hard gelatin, Opaque white, size 4 and contain the inactive ingredient: microcrystalline cellulose. Capsules are packaged in 60 cc HDPE bottles with induction seals and child-resistant polypropylene plastic caps. Each bottle contains 30 capsules with a silica gel desiccant.
- 10 mg capsules are hard gelatin, Swedish orange, size 4 and contain the inactive ingredient: microcrystalline cellulose. Capsules are packaged in white, 40 cc HDPE bottles with induction seals and child-resistant polypropylene plastic caps. Each bottle contains 10 capsules with a silica gel desiccant.

Storage: Store bottles refrigerated at 2-8ºC in the original manufacturer's container. Do not remove the desiccant.

If a storage temperature excursion is identified, promptly return TdCyd capsules to 2°-8°C and quarantine the supplies. Provide a detailed report of the excursion (including documentation of temperature monitoring and duration of the excursion) to PMBAfterHours@mail.nih.gov for determination of suitability.

Stability: Shelf life stability testing of the intact bottles is on-going. Dispense intact bottles of the original manufacturer's container when possible. If necessary, capsules may be repackaged and dispensed in a standard pharmacy prescription bottle. If re-packaged, capsules should be assigned a14-day expiration.

Route and Method of Administration: Oral. Take capsules by mouth on an empty stomach, either 1 hour before or 2 hours after meals.

8.2 Agent Ordering

NCI-supplied agents may be requested by the Principal Investigator (or their authorized designee) at each participating institution. Pharmaceutical Management Branch (PMB) policy requires that agent be shipped directly to the institution where the patient is to be treated. PMB does not permit the transfer of agents between institutions (unless prior approval from PMB is obtained). The CTEP-assigned protocol number must be used for ordering all CTEP-supplied investigational agents. The responsible investigator at each

participating institution must be registered with CTEP, DCTD through an annual submission of FDA Form 1572 (Statement of Investigator), Curriculum Vitae, Supplemental Investigator Data Form (IDF), and Financial Disclosure Form (FDF). If there are several participating investigators at one institution, CTEP-supplied investigational agents for the study should be ordered under the name of one lead investigator at that institution.

Active CTEP-registered investigators and investigator-designated shipping designees and ordering designees can submit agent requests through the PMB Online Agent Order Processing (OAOP) application (https://eapps-ctep.nci.nih.gov/OAOP/pages/login.jspx). Access to OAOP requires the establishment of a CTEP Identity and Access Management (IAM) account (https://ctepcore.nci.nih.gov/iam/index.jsp) and the maintenance of an "active" account status and a "current" password. For questions about drug orders, transfers, returns, or accountability, call (240) 276-6575 Monday through Friday between 8:30 am and 4:30 pm (ET) or e-mail PMBAfterHours@mail.nih.gov anytime.

8.3 Agent Accountability

Agent Inventory Records – The investigator, or a responsible party designated by the investigator, must maintain a careful record of the inventory and disposition of all agents received from DCTD using the NCI Drug Accountability Record Form (DARF). (See the CTEP Web site at http://ctep.cancer.gov for Procedures for Drug Accountability and Storage and to obtain a copy of the DARF Clinical Drug Request form.)

9 CORRELATIVE/SPECIAL STUDIES

Blood and urine will be obtained from all patients to determine the PK of TdCyd. Blood samples will also be collected for isolation of circulating tumor cells (CTCs) to measure re-expression of genes silenced by methylation.

Record the date, planned time and actual time of collection for each specimen on the PK/PD Sample Collection Sheet (Appendix E); the CTEP protocol number, unique patient accession number, and 3-digit sample number should be included on this form. In addition, the date and exact time when TdCyd was administered should be recorded on the form.

9.1 Pharmacokinetic Assays

TdCyd pharmacokinetics will be measured in plasma and urine from all patients during cycles 1-2 only. All PK measurements for this study will be analyzed in Dr. Larry Anderson's laboratory at FNLCR.

One 3-mL heparinized (green-top) tube preloaded with 30 µL of 100 mg/mL of ZEB will be obtained for each of the time points. The actual time of collection will be noted for each sample, and the sample will be placed immediately on ice. See Appendix D for reagents and the procedure for PK studies.

As of Amendment J (1/16/19), blood will be collected at the following times:

- prior to TdCyd administration
- cycle 1 day 1 at 1, 4, and 24 hours after TdCyd administration (before another dose is taken)
- cycle 1 day 8 prior to TdCyd administration
- cycle 1 day 15 (this sample is optional)
- cycle 2 day 1 prior to TdCyd administration and 1, 4, and 24 hours after TdCyd administration

(Prior to Amendment J [1/16/19], blood for PK was collected prior to TdCyd administration; cycle 1 day 1 at 15, 30, and 60 minutes and 2, 3, 4, and 24 hours after TdCyd administration [before another dose is taken]; cycle 1 day 8 prior to TdCyd administration; and optionally on cycle 1 day 15.)

*Patients who have been dose-adjusted will have optional PK samples collected prior to TdCyd administration on day 1 and then 1, 2, and 24 hours after drug administration (before the another dose is taken) during cycle 1 only.

The PK blood tubes/samples should be kept on ice until plasma can be recovered, at which time the plasma samples should be frozen (-70°C). The plasma samples should be stored at -70°C, and shipped to the PK lab at the address below on dry ice.

Based upon the results of the initial measurements, sampling times may be adjusted but neither the total number of samples nor the total amount of blood will be increased.

A baseline urine collection of 10 mL will be collected just before TdCyd administration. Urine will then be collected at every void from 0 to 24 hours on cycle 1, day 1 for PK analysis. Samples should be placed on wet ice immediately after collection and then refrigerated (see Appendix D). The total volume of urine collected for the 24-hour time period will be documented and a 10-mL sample will be taken from the total volume and saved; the rest can be discarded. PK samples will be shipped to:

Attn: [redacted]
Frederick National Laboratory for Cancer Research
OAD/DTP
[redacted]
[redacted]@mail.nih.gov [redacted]

9.2 Pharmacodynamic Assays

9.2.1 Blood Collection for CTC Studies

Blood will be collected for detection of CTCs to measure expression of genes silenced by methylation (e.g., p16, p15, MGMT, DAPK, TIMP3, RASSF1A). Whole blood will be collected aseptically by venipuncture or from a venous port into one 7.5 mL CellSave tube.

Blood samples for CTCs will be collected at the following times during cycles 1 and 2:

- cycle 1 prior to TdCyd administration
- cycle 1 day 1, 2 to 4 hours after TdCyd administration
- cycle 1 day 8 (at any time; this sample is optional)
- cycle 2 day 1 (at any time)
- day 1 of every subsequent cycle (at any time)

One additional blood sample will be collected at time of disease progression.

9.2.2 Laboratory Contact

At least 24 hours prior to tumor biopsy or blood sample collection, the research nurse will contact the NCI Phase I/II PK/PD Support Group in NIH Building 10: E-mail NCIPK-PDsupportgroup@mail.nih.gov, Pager: 102-12798 Phone: 301-451-1169 Fax: 301-480-5871. Initial processing and shipping of the samples will be completed as described below.

Testing and data analysis will be performed by Dr. Bob Kinders (PADIS/LHTP/FNLCR).

9.2.3 Sample Collection and Processing

Biospecimens will be collected and processed using validated SOPs that will ensure both specimen quality and patient confidentiality pursuant to informed consent provisions. Information about each specimen (e.g., blood, tumor biopsy, circulating tumor cells, per specific protocol) will be recorded on a PK/PD collection worksheet included in Appendix E.

Using a computerized inventory system and a backup hardcopy process, all specimen collection and processing steps will be documented and the specific location of each specimen will be tracked. Each new specimen collected will be assigned a unique barcode identifier that can be linked to the original specimen collected and other relevant information within the inventory system. To ensure patient confidentiality, only containers used for the initial specimen collections will be labeled with patient identifiers.

Only the barcode identifier will be applied to all subsequent specimen containers. When specimens are processed and aliquoted, no patient information will be included on the new containers. Original specimen containers will be discarded. Only barcode-labeled specimens without patient identifiers will be shipped for analysis and/or storage. Specimen labels will indicate: CTEP protocol number, unique patient accession number, 3-digit sample number (see list below), collection time, and total volume collected, as appropriate. Samples from sets of at least three patients will be grouped for scientific analysis.

Standardized 3-digit sample collection numbers:
100 series: urine for PK
200 series: blood for PK
300 series: blood for PD
400 series: blood for circulating tumor cells (CTCs)

The inventory process contains other security provisions sufficient to safeguard patient privacy and confidentiality. Access to the inventory system and associated documents will be restricted to appropriate individuals. Requests to use specimens stored in the repository must be approved. The only patient information available in the inventory system will be the patient sex, diagnosis, and level of informed consent given. SOPs ensure that any changes in informed consent made by a patient and relayed to the PI will be reflected in the inventory system to ensure that specimens are destroyed as appropriate. All laboratory personnel will be trained to adhere to SOPs and will be monitored for high-quality performance.

Any new use of these samples will require prospective IRB review and approval. Access to these samples will only be granted following IRB approval of an additional protocol, granting the rights to use the material.

If at any time, a patient withdraws from the study and does not wish for their existing samples to be utilized, the individual must provide a written request. Following receipt of this request, the samples will be destroyed (or returned to the patient, if so requested), and reported as such to the IRB. Any samples lost (in transit or by a researcher) or destroyed due to unknown sample integrity (i.e., broken freezer allows for extensive sample thawing, etc.) will be reported as such to the IRB.

9.2.4 Human Data Sharing Plan

What data will be shared?
We will share human data generated in this research for future research as follows:
x De-identified data in an NIH-funded or approved public repository
x Identified data in BTRIS (automatic for activities in the Clinical Center)
x De-identified or identified data with approved outside collaborators under appropriate agreements

How and where will the data be shared?
Data will be shared through:
x An NIH-funded or approved public repository: clinicaltrials.gov
x BTRIS (automatic for activities in the Clinical Center)
x Approved outside collaborators under appropriate individual agreements
x Publication and/or public presentations

When will the data be shared?
x At the time of publication or shortly thereafter

10 STUDY CALENDAR

Baseline evaluations are to be conducted within 1 week prior to start of protocol therapy. Diagnostic imaging must be done within 30 days prior to the start of therapy. Start of next cycle may be delayed for up to 1 week to accommodate scheduling conflicts. Treatments within a cycle may be delayed up to +/- 1 day to accommodate scheduling conflicts. History and physical examination and laboratory evaluations can be performed up to 3 days before the start of the next cycle. The research team may perform additional safety/monitoring tests as clinically indicated.

	Pre-Study	Cycle 1			Cycle 2			Cycle 3 onwards
		Wk1	Wk2	Wk3	Wk1	Wk2	Wk3	
TdCyd[a]		X	X		X	X		X
Informed consent	X							
Demographics	X							
Med. History	X				X			prior to treatment
Physical exam[b]	X	X	X		X			prior to treatment
ECG[b]	X		X					
Urinalysis	X							
Height and Weight	X				X			X
Performance Status	X	X	X		X			X
CBC w/diff, plts[c]	X	X	X		X			X
Serum chemistry[c]	X	X	X		X			X
Tumor measurement[d]	X							X
Radiologic evaluation[d]	X							X
B-HCG[e]	X				X			prior to treatment
PK blood and urine samples[f]	X	X	X		X			
Blood samples for CTCs[g]	X	X	X		X			start of every cycle
B-(1-3)-D glucan[h]								As clinically indicated
Adverse event evaluation		X……………………………………………………………………...…X						

a. TdCyd will be given orally on days 1-5 of each week for 2 weeks, with one week off, each 21-day cycle +/- 1 day for scheduling reasons (e.g., weekends, holidays, or patient convenience) as specified in the dose escalation table.

b. A physical examination including vital signs will be conducted pre-study, during weeks 1 and 2 of cycle 1, and then at the start of every cycle thereafter (within 3 days prior to treatment). ECG will be performed pre-study, on cycle 1 day 8 (+/-1 day), and as clinically indicated.

c. Serum chemistries (sodium, potassium, chloride, bicarbonate, BUN, creatinine, glucose, AST, ALT, bilirubin, calcium, phosphorous, albumin, magnesium, alkaline phosphatase, LDH) and CBC with differential and platelets will be performed pre-study, during weeks 1 and 2 of cycle 1, and then and at the start (within 3 days prior to treatment) during week 1 of every cycle thereafter. *Additional samples may be drawn in week 2 as clinically indicated to ensure patient safety.*

d. Radiologic examination will be performed at baseline and then every 2 cycles (3 cycles for patients on study for more than one year, 4 cycles for those on study more than 3 years).

e. Urine or serum pregnancy test (women of childbearing potential) will be conducted within 24 hours prior to TdCyd administration at baseline, and within 24 hours prior to TdCyd at the start of each cycle thereafter.

f. Blood and urine for PK analysis will be obtained from all patients as specified in Section 9.1.

g. Blood samples for CTCs will be collected from patients as specified in Section 9.2.
h. Serum samples for beta B-(1-3)-D glucan assay analysis will be collected as clinically indicated for *Pneumocystis jiroveci* pneumonia (PJP)

11 MEASUREMENT OF EFFECT

11.1 Antitumor Effect – Solid Tumors

Although response is not the primary endpoint of this trial, patients with measurable disease will be assessed by standard criteria. For the purposes of this study, patients should be re-evaluated for response every 6 weeks (every 2 cycle; 3 cycles for patients on study more than one year, 4 cycles for those on study more than 3 years). In addition to a baseline scan, confirmatory scans should also be obtained at least 4 weeks following initial documentation of objective response.

Response and progression will be evaluated in this study using the new international criteria proposed by the revised Response Evaluation Criteria in Solid Tumors (RECIST) guideline (version 1.1) [*Eur J Ca* 45:228-247, 2009]. Changes in the largest diameter (unidimensional measurement) of the tumor lesions and the shortest diameter in the case of malignant lymph nodes are used in the RECIST criteria.

11.1.1 Definitions

Evaluable for toxicity: All patients will be evaluable for toxicity from the time of their first treatment.

Evaluable for objective response: Only those patients who have measurable disease present at baseline, have received at least one cycle of therapy, and have had their disease re-evaluated will be considered evaluable for response. These patients will have their response classified according to the definitions stated below. (Note: Patients who exhibit objective disease progression prior to the end of cycle 1 will also be considered evaluable.)

Evaluable Non-Target Disease Response: Patients who have lesions present at baseline that are evaluable but do not meet the definitions of measurable disease, have received at least one cycle of therapy, and have had their disease re-evaluated will be considered evaluable for non-target disease. The response assessment is based on the presence, absence, or unequivocal progression of the lesions.

11.1.2 Disease Parameters

Measurable disease: Measurable lesions are defined as those that can be accurately measured in at least one dimension (longest diameter to be recorded) as ≥20 mm by chest x-ray or as ≥10 mm with CT scan, MRI, or

calipers by clinical exam. All tumor measurements must be recorded in millimeters (or decimal fractions of centimeters).

Malignant lymph nodes: To be considered pathologically enlarged and measurable, a lymph node must be ≥15 mm in short axis when assessed by CT scan (CT scan slice thickness recommended to be no greater than 5 mm). At baseline and in follow-up, only the short axis will be measured and followed.

Non-measurable disease: All other lesions (or sites of disease), including small lesions (longest diameter <10 mm or pathological lymph nodes with ≥10 to <15 mm short axis), are considered non-measurable disease. Bone lesions, leptomeningeal disease, ascites, pleural/pericardial effusions, lymphangitis cutis/pulmonitis, inflammatory breast disease, and abdominal masses (not followed by CT or MRI), are considered as non-measurable.

Note: Cystic lesions that meet the criteria for radiographically defined simple cysts should not be considered as malignant lesions (neither measurable nor non-measurable) since they are, by definition, simple cysts.

'Cystic lesions' thought to represent cystic metastases can be considered as measurable lesions, if they meet the definition of measurability described above. However, if non-cystic lesions are present in the same patient, these are preferred for selection as target lesions.

Target lesions: All measurable lesions up to a maximum of 2 lesions per organ and 5 lesions in total, representative of all involved organs, should be identified as **target lesions** and recorded and measured at baseline. Target lesions should be selected on the basis of their size (lesions with the longest diameter), be representative of all involved organs, but in addition should be those that lend themselves to reproducible repeated measurements. It may be the case that, on occasion, the largest lesion does not lend itself to reproducible measurement in which circumstance the next largest lesion which can be measured reproducibly should be selected. A sum of the diameters (longest for non-nodal lesions, short axis for nodal lesions) for all target lesions will be calculated and reported as the baseline sum diameters. If lymph nodes are to be included in the sum, then only the short axis is added into the sum. The baseline sum diameters will be used as reference to further characterize any objective tumor regression in the measurable dimension of the disease.

Non-target lesions: All other lesions (or sites of disease) including any measurable lesions over and above the 5 target lesions should be identified as **non-target lesions** and should also be recorded at baseline.

Measurements of these lesions are not required, but the presence, absence, or in rare cases unequivocal progression of each should be noted throughout follow-up.

11.2 Guidelines for Evaluation of Measurable Disease

All measurements should be taken and recorded in metric notation using a ruler or calipers. All baseline evaluations should be performed as closely as possible to the beginning of treatment and never more than 4 weeks before the beginning of the treatment.

The same method of assessment and the same technique should be used to characterize each identified and reported lesion at baseline and during follow-up. Imaging-based evaluation is preferred to evaluation by clinical examination unless the lesion(s) being followed cannot be imaged but are assessable by clinical exam.

Clinical lesions: Clinical lesions will only be considered measurable when they are superficial (e.g., skin nodules and palpable lymph nodes) and ≥10 mm diameter as assessed using calipers (e.g., skin nodules). In the case of skin lesions, documentation by color photography, including a ruler to estimate the size of the lesion, is recommended.

Chest x-ray: Lesions on chest x-ray are acceptable as measurable lesions when they are clearly defined and surrounded by aerated lung. However, CT is preferable.

Conventional CT and MRI: This guideline has defined measurability of lesions on CT scan based on the assumption that CT slice thickness is 5 mm or less. If CT scans have slice thickness greater than 5 mm, the minimum size for a measurable lesion should be twice the slice thickness. MRI is also acceptable in certain situations (e.g. for body scans).

As with CT, if an MRI is performed, the technical specifications of the scanning sequences used should be optimized for the evaluation of the type and site of disease. Furthermore, as with CT, the modality used at follow-up should be the same as was used at baseline and the lesions should be measured/assessed on the same pulse sequence. It is beyond the scope of the RECIST guidelines to prescribe specific MRI pulse sequence parameters for all scanners, body parts, and diseases. Ideally, the same type of scanner should be used and the image acquisition protocol should be followed as closely as possible to prior scans. Body scans should be performed with breath-hold scanning techniques, if possible.

PET-CT: At present, the low dose or attenuation correction CT portion of a combined PET-CT is not always of optimal diagnostic CT quality for use with RECIST measurements. However, if the site can document that the CT performed as part of a PET-CT is of identical diagnostic quality to a diagnostic CT (with IV and oral contrast), then the CT portion of the PET-CT can be used for RECIST measurements and can be used interchangeably with conventional CT in accurately measuring cancer lesions over time. Note, however, that the PET portion of the CT introduces additional data which may bias an investigator if it is not routinely or serially performed.

Ultrasound: Ultrasound is not useful in assessment of lesion size and should not be used as a method of measurement. Ultrasound examinations cannot be reproduced in their entirety for independent review at a later date and, because they are operator dependent, it cannot be guaranteed that the same technique and measurements will be taken from one assessment to the next. If new lesions are identified by ultrasound in the course of the study, confirmation by CT or MRI is advised. If there is concern about radiation exposure at CT, MRI may be used instead of CT in selected instances.

Endoscopy, Laparoscopy: The utilization of these techniques for objective tumor evaluation is not advised. However, such techniques may be useful to confirm complete pathological response when biopsies are obtained or to determine relapse in trials where recurrence following complete response (CR) or surgical resection is an endpoint.

Tumor markers: Tumor markers alone cannot be used to assess response. If markers are initially above the upper normal limit, they must normalize for a patient to be considered in complete clinical response. Specific guidelines for both CA-125 response (in recurrent ovarian cancer) and PSA response (in recurrent prostate cancer) have been published [*JNCI* 96:487-488, 2004; *J Clin Oncol* 17, 3461-3467, 1999; *J Clin Oncol* 26:1148-1159, 2008]. In addition, the Gynecologic Cancer Intergroup has developed CA-125 progression criteria which are to be integrated with objective tumor assessment for use in first-line trials in ovarian cancer [*JNCI* 92:1534-1535, 2000].

Cytology, Histology: These techniques can be used to differentiate between partial responses (PR) and complete responses (CR) in rare cases (e.g., residual lesions in tumor types, such as germ cell tumors, where known residual benign tumors can remain).

The cytological confirmation of the neoplastic origin of any effusion that appears or worsens during treatment when the measurable tumor has met criteria for response or stable disease is mandatory to differentiate between response or stable disease (an effusion may be a side effect of the treatment) and progressive disease.

FDG-PET: While FDG-PET response assessments need additional study, it is sometimes reasonable to incorporate the use of FDG-PET scanning to complement CT scanning in assessment of progression (particularly possible 'new' disease). New lesions on the basis of FDG-PET imaging can be identified according to the following algorithm:

a. Negative FDG-PET at baseline, with a positive FDG-PET at follow-up is a sign of PD based on a new lesion.
b. No FDG-PET at baseline and a positive FDG-PET at follow-up: If the positive FDG-PET at follow-up corresponds to a new site of disease confirmed by CT, this is PD. If the positive FDG-PET at follow-up is not confirmed as a new site of disease on CT, additional follow-up CT scans are needed to determine if there is truly progression occurring at that site (if so, the date of PD will be the date of the initial abnormal FDG-PET scan). If the positive FDG-PET at follow-up corresponds to a pre-existing site of disease on CT that is not progressing on the basis of the anatomic images, this is not PD.

c. FDG-PET may be used to upgrade a response to a CR in a manner similar to a biopsy in cases where a residual radiographic abnormality is thought to represent fibrosis or scarring. The use of FDG-PET in this circumstance should be prospectively described in the protocol and supported by disease-specific medical literature for the indication. However, it must be acknowledged that both approaches may lead to false positive CR due to limitations of FDG-PET and biopsy resolution/sensitivity.

Note: A 'positive' FDG-PET scan lesion means one which is FDG avid with an uptake greater than twice that of the surrounding tissue on the attenuation corrected image.

11.3 Response Criteria

11.3.1 Evaluation of Target Lesions

<u>Complete Response (CR)</u>: Disappearance of all target lesions. Any pathological lymph nodes (whether target or non-target) must have reduction in short axis to <10 mm.

<u>Partial Response (PR)</u>: At least a 30% decrease in the sum of the diameters of target lesions, taking as reference the baseline sum diameters.

<u>Progressive Disease (PD)</u>: At least a 20% increase in the sum of the diameters of target lesions, taking as reference the smallest sum on study (this includes the baseline sum if that is the smallest on study). In addition to the relative increase of 20%, the sum must also demonstrate an absolute increase of at least 5 mm. (Note: the appearance of one or more new lesions is also considered progressions).

<u>Stable Disease (SD)</u>: Neither sufficient shrinkage to qualify for PR nor sufficient increase to qualify for PD, taking as reference the smallest sum diameters while on study.

11.3.2 Evaluation of Non-Target Lesions

<u>Complete Response (CR)</u>: Disappearance of all non-target lesions and normalization of tumor marker level. All lymph nodes must be non-pathological in size (<10 mm short axis).

Note: If tumor markers are initially above the upper normal limit, they must normalize for a patient to be considered in complete clinical response.

<u>Non-CR/Non-PD:</u> Persistence of one or more non-target lesion(s) and/or maintenance of tumor marker level above the normal limits.

<u>Progressive Disease (PD)</u>: Appearance of one or more new lesions and/or *unequivocal progression* of existing non-target lesions. *Unequivocal progression* should not normally trump target lesion status. It must be representative of overall disease status change, not a single lesion increase.

Although a clear progression of "non-target" lesions only is exceptional, the opinion of the treating physician should prevail in such circumstances, and the progression status should be confirmed at a later time by the review panel (or Principal Investigator).

11.3.3 Evaluation of Best Overall Response

The best overall response is the best response recorded from the start of the treatment until disease progression/recurrence (taking as reference for progressive disease the smallest measurements recorded since the treatment started). The patient's best response assignment will depend on the achievement of both measurement and confirmation criteria.

For Patients with Measurable Disease (i.e., Target Disease)

Target Lesions	Non-Target Lesions	New Lesions	Overall Response	Best Overall Response when Confirmation is Required*
CR	CR	No	CR	≥ 4 wks. Confirmation**
CR	Non-CR/Non-PD	No	PR	≥ 4 wks. Confirmation**
CR	Not evaluated	No	PR	
PR	Non-CR/Non-PD/not evaluated	No	PR	
SD	Non-CR/Non-PD/not evaluated	No	SD	Documented at least once ≥ 4 wks. from baseline**
PD	Any	Yes or No	PD	no prior SD, PR or CR
Any	PD***	Yes or No	PD	
Any	Any	Yes	PD	

* See RECIST 1.1 manuscript for further details on what is evidence of a new lesion.
** Only for non-randomized trials with response as primary endpoint.
*** In exceptional circumstances, unequivocal progression in non-target lesions may be accepted as disease progression.

<u>Note</u>: Patients with a global deterioration of health status requiring discontinuation of treatment without objective evidence of disease progression at that time should be reported as "*symptomatic deterioration.*" Every effort should be made to document the objective progression even after discontinuation of treatment.

For Patients with Non-Measurable Disease (i.e., Non-Target Disease)

Non-Target Lesions	New Lesions	Overall Response
CR	No	CR
Non-CR/non-PD	No	Non-CR/non-PD*
Not all evaluated	No	not evaluated
Unequivocal PD	Yes or No	PD
Any	Yes	PD
* 'Non-CR/non-PD' is preferred over 'stable disease' for non-target disease since SD is increasingly used as an endpoint for assessment of efficacy in some trials so to assign this category when no lesions can be measured is not advised		

11.4 Duration of Response

Duration of overall response: The duration of overall response is measured from the time measurement criteria are met for CR or PR (whichever is first recorded) until the first date that recurrent or progressive disease is objectively documented (taking as reference for progressive disease the smallest measurements recorded since the treatment started).

The duration of overall CR is measured from the time measurement criteria are first met for CR until the first date that progressive disease is objectively documented.

Duration of stable disease: Stable disease is measured from the start of the treatment until the criteria for progression are met, taking as reference the smallest measurements recorded since the treatment started, including the baseline measurements.

12 DATA REPORTING / REGULATORY REQUIREMENTS

Adverse event lists, guidelines, and instructions for AE reporting can be found in Section 7.

12.1 Data Reporting

12.1.1 Method

This study will be monitored by the Clinical Data Update System (CDUS). Cumulative CDUS data will be submitted quarterly to CTEP by electronic means. Reports are due January 31, April 30, July 31, and October 31.

Note: **All** adverse events that have occurred on the study, including those reported through CTEP-AERS, must be reported via the monitoring method identified above.

12.1.2 Responsibility for Data Submission

N/A

12.2 CTEP Multicenter Guideline

N/A

12.3 Collaborative Agreements Language

N/A

13 STATISTICAL CONSIDERATIONS

The primary objective of this trial is to establish the safety, tolerability, and MTD of oral TdCyd administered daily for 5 days a week for 2 weeks, with one week off, in 21-day cycles, to patients with refractory solid tumors. This study will follow design 4 of the Simon accelerated titration designs [18], with dose escalation proceeding in 1-patient cohorts. We will accrue to the next dose level only after we have established safety at lower dose levels. This accelerated titration will stop in the event of one DLT (defined in Section 5.2) at which point the study will convert to the traditional 3+3 dose escalation design (see Section 5.2). This study permits intra-patient dose modification (defined in Section 5).

Secondary objectives are to determine the pharmacokinetics of oral TdCyd, to document preliminary evidence of TdCyd activity, and to determine effect of study treatment on re-expression of select genes silenced by methylation in circulating tumor cells.
Exploratory evaluations will be performed on all PD and PK parameters, with results of these tests reported with appropriate caveats about the exploratory nature of the analysis, and without formal adjustment for multiple comparisons.

13.1 Sample Size/Accrual Rate

If all dose levels are evaluated, we anticipate enrolling a minimum of 6 patients and a maximum of 42 patients. In order to allow for a small number of patients who may not be evaluable, the accrual ceiling for this trial is set at 46 patients. It is anticipated that 2-3 patients may be enrolled per month onto this study. It is expected that 48 months will be required to accrue the number of patients necessary to complete the trial.

13.2 Analysis of Secondary Endpoints

N/A

14 HUMAN SUBJECTS PROTECTIONS

14.1 Justification for Subject Selection

This study will be open to all individuals regardless of gender, ethnicity, or race, provided that the aforementioned inclusion and exclusion criteria are met. Patients for this study will

be recruited through internal referral, our physician referral base, and through various cancer information hotlines (i.e., Clinical Studies Support Center, 1-800-4Cancer). To date, there is no information that suggests that differences in drug metabolism or effect on tumor would be expected in one ethnic group compared to another. Efforts will be made to extend accrual to each representative population, but a balance must be struck between participant safety considerations and limitations on the number of individuals exposed to potentially ineffective treatments on the one hand and the need to explore racial/ethnic aspects of clinical research on the other hand. If differences in outcome that correlate to ethnic identity are noted, a follow-up study may be written to investigate those differences more fully.

Because TdCyd is a nucleoside analog likely to have teratogenic effects, pregnant and nursing women will be excluded from this trial. Patients with unstable or serious medical conditions are excluded due to the possibility that the underlying condition may obscure the attribution of effect and adverse events with respect to TdCyd, and may limit study compliance.

14.1.1 Participation of Children

This study includes patients 18 years of age and older. Because no clinical dosing or adverse event data are currently available on the use of TdCyd in patients <18 years of age, children are excluded from this study, but may be eligible for future pediatric trials.

14.2 Evaluation of Benefits and Risks/Discomforts

There may or may not be any clinical benefit to a patient from participation in this trial. Their participation will benefit future cancer patients. Potential risks include the possible occurrence of any of a range of side effects that are listed in the consent document. The procedure for protecting against or minimizing risks will be to medically evaluate patients as described in Section 5 and Section 6. Although no compensation is available, any injury will be fully evaluated and treated in keeping with the benefits or care to which participants are entitled under applicable regulations.

14.3 Consent and Assent Process and Documentation

An associate or principal investigator on the trial will inform patients of the purpose, alternatives, drug administration plan, research objectives, and follow-up of this trial. The patient will be provided an IRB-approved consent for review and signature and his/her questions will be answered. After a decision is made to enroll into the study, a signature will be obtained from the patient. The original signed consent goes to Medical Records; a copy will be placed in the research record. Patients will not be consented by telephone.

All patients must have a signed informed consent form and an on-study (confirmation of eligibility) form filled out and signed by a participating investigator before entering on study.

Adults unable to give consent are excluded from enrolling in the protocol. However, re-consent may be necessary and there is a possibility, though unlikely, that subjects could become decisionally impaired. For this reason and because there is a prospect of direct benefit from research participation, all subjects ≥ age 18 at the NCI only will be offered the opportunity to fill in their wishes for research and care, and assign a substitute decision maker on the “NIH Advance Directive for Health Care and Medical Research Participation” form so that another person can make decisions about their medical care in the event that they become incapacitated or cognitively impaired during the course of the study. Note: The PI or AI will contact the NIH Ability to Consent Assessment Team for evaluation. For those subjects that become incapacitated and do not have pre-determined substitute decision maker, the procedures described in MEC Policy 87-4 for appointing a surrogate decision maker for adult subjects who are (a) decisionally impaired, and (b) who do not have a legal guardian or durable power of attorney, will be followed.

14.4 Procedure for Protecting Against or Minimizing Any Potential Risks

All care will be taken to minimize side effects, but they can be unpredictable in nature and severity. This study may involve risks to patients, which are currently unforeseeable. All patients will be monitored for side effects from taking study medication. This research represents a greater than minimal risk to participants, but presents the prospect of direct benefit to individual subjects.

14.5 Patient Advocate

The patients’ rights representative is available to patients receiving treatment on this protocol at the NIH Clinical Center at (301) 496-2626 in Building 10 of the Clinical Research Center, Room 1-3521, on the Bethesda NIH campus. Patients will be informed that they can contact the study PI or RN at any time with questions about their medical care, and that the patients’ rights representative is also available to answer non-medical questions about the study.

REFERENCES

1. Jones, P.A. and S.B. Baylin, *The fundamental role of epigenetic events in cancer.* Nat Rev Genet, 2002. **3**(6): p. 415-428.

2. Jones, P.A. and S.B. Baylin, *The Epigenomics of Cancer.* Cell, 2007. **128**(4): p. 683-692.

3. Baylin, S.B. and J.E. Ohm, *Epigenetic gene silencing in cancer - a mechanism for early oncogenic pathway addiction?* Nat Rev Cancer, 2006. **6**(2): p. 107-116.

4. Cowan, L.A., S. Talwar, and A.S. Yang, *Will DNA methylation inhibitors work in solid tumors? A review of the clinical experience with azacitidine and decitabine in solid tumors.* Epigenomics, 2010. **2**(1): p. 71-86.

5. Esteller, M., et al., *A Gene Hypermethylation Profile of Human Cancer.* Cancer Research, 2001. **61**(8): p. 3225-3229.

6. Ooi, S.K.T. and T.H. Bestor, *Cytosine Methylation: Remaining Faithful.* Current Biology, 2008. **18**(4): p. R174-R176.

7. Ooi, S.K.T., A.H. O'Donnell, and T.H. Bestor, *Mammalian cytosine methylation at a glance.* Journal of Cell Science, 2009. **122**(16): p. 2787-2791.

8. Singh, V., P. Sharma, and N. Capalash, *DNA Methyltransferase-1 Inhibitors as Epigenetic Therapy for Cancer.* Current Cancer Drug Targets, 2013. **13**(4): p. 379-399.

9. Jones, P.A., et al., *Cell cycle-specific reactivation of an inactive X-chromosome locus by 5-azadeoxycytidine.* Proceedings of the National Academy of Sciences, 1982. **79**(4): p. 1215-1219.

10. Secrist, J.A., et al., *Synthesis and biological activity of 2'-deoxy-4'-thio pyrimidine nucleosides.* Journal of Medicinal Chemistry, 1991. **34**(8): p. 2361-2366.

11. Parker, W.B., et al., *Metabolism of 4'-thio-β-d-arabinofuranosylcytosine in CEM cells.* Biochemical Pharmacology, 2000. **60**(12): p. 1925-1932.

12. Kinders, R.J., et al., *Abstract 2306: Pre-clinical development of 4'-thio-2'-deoxycytidine (TdCyd) as a DNA-demethylating agent for use in treating solid tissue tumors.* Cancer Research, 2014. **74**(19 Supplement): p. 2306.

13. Kumar, S., et al., *DNA containing 4'-thio-2'-deoxycytidine inhibits methylation by HhaI methyltransferase.* Nucleic Acids Research, 1997. **25**(14): p. 2773-2783.

14. Thottassery, J., et al., *Novel DNA methyltransferase-1 (DNMT1) depleting anticancer nucleosides, 4'-thio-2'-deoxycytidine and 5-aza-4'-thio-2'-deoxycytidine.* Cancer Chemotherapy and Pharmacology, 2014. **74**(2): p. 291-302.

15. Daniel, F.I., et al., *The role of epigenetic transcription repression and DNA methyltransferases in cancer.* Cancer, 2011. **117**(4): p. 677-87.

16. Chen, T., et al., *A Novel Dnmt3a Isoform Produced from an Alternative Promoter Localizes to Euchromatin and Its Expression Correlates with Activede Novo Methylation.* Journal of Biological Chemistry, 2002. **277**(41): p. 38746-38754.

17. Onishi, A., et al., *Diagnostic Accuracy of Serum 1,3-β-d-Glucan for Pneumocystis jiroveci Pneumonia, Invasive Candidiasis, and Invasive Aspergillosis: Systematic Review and Meta-Analysis.* Journal of Clinical Microbiology, 2012. **50**(1): p. 7-15.

18. Simon, R., et al., *Accelerated titration designs for phase I clinical trials in oncology.* J Natl Cancer Inst, 1997. **89**(15): p. 1138-47.

APPENDIX A: Performance Status Criteria

ECOG Performance Status Scale	
Grade	Descriptions
0	Normal activity. Fully active, able to carry on all pre-disease performance without restriction.
1	Symptoms, but ambulatory. Restricted in physically strenuous activity, but ambulatory and able to carry out work of a light or sedentary nature (e.g., light housework, office work).
2	In bed <50% of the time. Ambulatory and capable of all self-care, but unable to carry out any work activities. Up and about more than 50% of waking hours.
3	In bed >50% of the time. Capable of only limited self-care, confined to bed or chair more than 50% of waking hours.
4	100% bedridden. Completely disabled. Cannot carry on any self-care. Totally confined to bed or chair.
5	Dead.

APPENDIX B: Patient Study Diary

Today's date ___________________ **Agent: TdCyd**

Patient Name___________________(*initials acceptable*) **Patient Study ID _________________**

INSTRUCTIONS TO THE PATIENT:

1. Complete one form for each cycle (21 days) of treatment.
2. You will take ________ capsules (____ mg per capsule) of **TdCyd** on days 1-5 and 8-12 of each cycle only. TdCyd should be taken with water only, at least 1 hour before or 2 hours after a meal.
3. Record the date and times when you took TdCyd.
4. If you miss or vomit the dose, please make a note of this in the Comments column and do not make up the dose.
5. If you have any comments or notice any side effects, please record them in the Comments column.
6. Please bring this form and any unused **TdCyd** when you return for each appointment.

Day	Date	Dose Level	Time of TdCyd dose	Dose Taken	Comments
1					
2					
3					
4					
5					
6	----------		----------		
7	----------		----------		
8					
9					
10					
11					
12					
13	----------		----------		
14	----------		----------		
15	----------		----------		
16	----------		----------		
17	----------		----------		
18	----------		----------		
19	----------		----------		
20	----------		----------		
21	----------		----------		

Patient's signature:____

APPENDIX C: Patient Study Calendar

The study drugs are given over a 21-day period of time called cycles. The 21-day treatment cycle will be repeated as long as you are tolerating the medications and your cancer is either stable or getting better. The chart shows what will happen to you during cycle 1 and future cycles after you sign the consent form and start the study. Each cycle is numbered. The left-hand column shows the day in the cycle, and the right-hand column tells you what will happen on that day.

Day	What to do and what will happen to you
**Before starting study drug**	• Have a history taken of how you feel and undergo a physical examination by a Health Care Provider<br>• Get routine blood and urine tests<br>• Pregnancy test for women who are able to become pregnant<br>• CT or MRI scan will be done<br>• ECG will be done to check your heart
**Cycle 1, Days 1-5**	• Have a history taken of how you feel and undergo a physical examination by a Health Care Provider<br>• Get routine blood tests<br>• Start taking TdCyd by mouth each day for 5 days<br>• Blood draws for research will be obtained<br>• Urine samples for research will be obtained over a 24-hour period
**Cycle 1, Days 6,7**	• No treatment
**Cycle 1, Days 8-12**	• Have a history taken of how you feel and undergo a physical examination by a Health Care Provider<br>• Get routine blood tests<br>• Take TdCyd each day for 5 days<br>• Blood draws for research will be obtained on day 8 (optional)<br>• ECG will be done to check your heart on day 8
**Cycle 1, Day 15**	• No treatment<br>• Blood draws for research will be obtained (optional)
**Cycle 2, Days 1-5**	• Get routine blood tests<br>• Blood draws for research will be obtained on days 1 and 2<br>• Take TdCyd each day for 5 days
**Cycle 2, Days 6, 7**	• No treatment
**Cycle 2, Days 8-12**	• Take TdCyd each day for 5 days
**Cycle 2, Days 13-21**	• No treatment
**Cycle 3 and onwards**	• Blood draws for research will be obtained on day 1 of every cycle<br>• Imaging tests to determine how your tumor is responding to the treatment will be done every 6 weeks (9 weeks for patients on study for more than one year, 12 weeks for those on study more than 3 years)<br>• Pregnancy test for women who are able to become pregnant

APPENDIX D: Procedure for Blood and Urine Samples for Pharmacokinetics

Preparation of tubes:
Zebularine (ZEB) bulk powder will be provided upon request to the NCI Phase I/II PK/PD Support Group who will make up a stock solution at 100 mg/mL (wt of zebularine/volume of water added) and prepare 1-mL frozen aliquots.

Blood Draw Tubes:
ZEB can be added to Vacutainer tubes up to 1 day in advance without causing significant loss of vacuum. Using a 3/10 cc insulin syringe or other similar sized syringe with a fine needle, draw up 30 µL of the ZEB solution and transfer it to a 3-mL heparinized Vacutainer tube (cat #366667) by piercing the stopper. Do not draw up ZEB for more than 1 tube at a time. You will not be able to control the amount of ZEB solution that leaves the needle, as it is sucked out by the vacuum. Because of the fine needle, you will not lose the vacuum (apart from the volume added) in the collection tube.

Urine Storage Cryogenic Vials:
Add 5 µL of the 100 mg/mL ZEB solution to each cryogenic vial to accommodate the 0.5 mL urine sample. Because the cryogenic tubes are not under vacuum, you can use any appropriate device to measure the 5 µL, e.g., a micropipettor such as Pipetman. This will yield the same final concentration of ZEB in the urine samples as in the blood samples (final concentration 1 mg/mL).

Plasma:
Supplies for each sample:
- One (1) 3-mL heparinized (green-top) tube (Vacutainer · cat #366667 or equivalent) **with zebularine**
- It is critical that the vials containing ZEB be used to minimize ex vivo deamination of TdCyd during processing and storage.
- Three (3) 1-mL cryogenic vials

Procedure: Obtain blood in a 3-mL heparinized (green-top) tube (Vacutainer cat #366667 or equivalent) preloaded with 30 µL of 100 mg/mL of **ZEB**. Gently invert 3 times to mix and place immediately on ice. For each time point, label 3 cryogenic vials with the CTEP protocol number, unique patient accession number, 3-digit sample number, date, and time. Do not include patient identifiers on the sample labels. Centrifuge the blood at 600 x g for 10 minutes at 4°C. Transfer 0.5 mL of the plasma into each of 2 cryogenic vials and the remainder of the plasma into the third cryogenic vial, if possible without disturbing the cell pellet. Keep the plasma on ice and freeze at or below -20°C within 1 hour.

Urine:
Supplies for each sample:
- One (1) screw-capped centrifuge tube (15 mL)
- Three (3) 1-mL cryogenic vials with 5 µL of 100 mg/mL zebularine (ZEB)
- It is critical that the vials containing ZEB be used to minimize ex vivo deamination of TdCyd during storage.

Procedure: Urine will be collected at every void from 0 to 24 hours post-treatment on day 1 in cycle 1 for PK analysis. Start urine collection immediately after study drug is administered, store refrigerated (2-8°C), and at the end of 24 hours retain a 10 mL aliquot only; place on ice and discard the remainder. Pour the urine specimen into a screw-capped centrifuge tube and centrifuge at 600 g for 1 minute at 4°C to sediment debris. Transfer 0.5 mL of the urine into each of 3 cryogenic vials **with ZEB**. Label the vials with the CTEP protocol number, unique patient accession number, 3-digit sample number, and date (no patient identifiers). Freeze the cryogenic vials of urine at or below -20°C.

Materials and Methods for Analysis of Thiopyrimidines in Plasma

4'-Thio-2-'deoxycytidine (TdCyd; NSC 764276), 4'-Thio-2'-deoxyuridine (TdUrd; NSC 776952) 4'-Thio-thymidine (TdThd; NSC 776949), and 2',2''-difluorodeoxycytidine (gemcitabine; NSC 613327) were obtained from DTP, NCI. $^{13}C_4$-2'-deoxyuridine ($^{13}C_4$-dUrd) and $^{13}C_5$-thymidine ($^{13}C_5$-dThd) were obtained from Cambridge Isotopes Labs. All other chemicals and reagents were obtained from Sigma-Aldrich.

Blood samples were collected into tubes containing heparin-lithium and tetrahydrouridine (THU), and immediately put on ice. Plasma was separated by centrifugation, and stored at -20°C until analysis. 100 µL aliquots of thawed plasma were mixed with 3.0 volumes of acetonitrile that contained 0.15 µM gemcitabine, 0.1 µM $^{13}C_4$-2'-deoxyuridine, and 0.1 µM $^{13}C_5$-thymidine as internal standards. The samples were vortexed for at least 10 sec, and then centrifuged at 10,000g for 10 minutes. Supernatant was collected and dried under vacuum centrifugation. The residue was re-suspended in 100 µL phosphate buffered saline, and 10 µL aliquots were injected into the LC/MS. Plasma standards were prepared by adding known amounts of the 4'-thiopyrmidnes.

Separation was accomplished on a 2.0 x 150 mm Gemini C18, 5 µM column (Phenomenex). The mobile phase consisted of a two component mixture; component A was 13 mM ammonium acetate, pH 9.0 with NH4OH buffer and component B was methanol. Initial conditions were flow at 0.2mL/min and 2% methanol. A multistep gradient program was used to elute the analytes as follows; zero to 0.5 min, 2% methanol; 0.5 to 1.0 min, 7% methanol; 1.0 to 10 min, 25% methanol; 10 to 11 min, 85% methanol; and 11 to 13 min held at 85% methanol. The column was re-equilibrated for 5.5 min at 2% methanol, flow rate 0.25 mL/min prior to the next injection. The compounds of interest eluted from the column in the time range of 7 to 11min.

Detection and quantitation of the 4'-thio-pyrmidines were accomplished by using a Thermo Q-Exactive Mass Spectrometer (Thermo). Compounds eluting from the LC were ionized using a heated electrospray source (280°C vaporizer, 25 units sheath gas, 285°C transfer tube). The mass spectrometer was operated in full scan mode (100-500amu) and at a resolution of 70,000. Samples were injected twice to collect negative and positive ions. Peak areas from either the $M-H^-$ or $M+H^+$ ion tracings with a mass tolerance of 20 ppm of the compounds of interest were used for analysis. Peak area ratios of the molecular ions of TdCyd to gemcitabine, TdUrd to $^{13}C_4$-dUrd, and TdThd to $^{13}C_5$-dThd were used to construct response curves from the plasma standards and applied to the plasma samples. Plasma standards were found to exhibit a linear response in

relation to concentration over the range of 25 nM to 25 μM for TdCyd, 5 μM to 25 μM for TdUrd, and 2.5 nM to 25 μM for TdThd when analyzing samples and standards in negative ion mode. A linear response to plasma concentration of 5 nM to 10 μM was found when TdCyd in analyzed in positive ion mode.

APPENDIX E: PK/PD Sample Collection Worksheets

**CTEP Protocol P9883**<br>**Dose level:** **TdCyd Dose:**<br>**Patient ID:**		**Page ____________**<br>**for Sample Pick-up**<br>**Lab phone: ___________**	**Research Nurse:**<br>Phone:<br>**Lead AI/PI:**<br>Phone: Pager:
**PLEASE ENTER DATE, TIME, and VOLUME OF EACH VOID FOR 24 HOURS POST DRUG ADMINISTRATION**<br>**KEEP SAMPLES REFRIGERATED UNTIL PICKED UP**			
**Date**	**Time**	**Volume of Void**	**Record comments (i.e., if collection missed), and sign each time you collect a sample**
**Pre-Drug Administration (Sample 100)**			
**DAY 1 Start 24 hour urine collection immediately after study drug administration, keep on ice. Record volume of each void on this sheet and record total 24 hour volume at the end of the collection. At the end of 24 hours, retain 10 mL aliquot only, place on ice and discard remainder of urine.**			
**Date**	**Time** (start of 24 hour collection time)	**Volume of Void**	**24-hour urine collection**
			**Volume 1**
			**Volume 2**
			**Volume 3**
			**Volume 4**
			**Volume 5**
			**Volume 6**
			**Volume 7**
			**Volume 8**
			**Volume 9**
			**Volume 10**
**Total** (24-hour volume)			**10 mL aliquot obtained** ☐<br>**(please check box)** **Sample #101**
**End 24-hour Collection Time:**			

Date:	PK/PD BLOOD SAMPLE COLLECTION SHEET: CYCLE 1 DAY 1				
**CTEP Protocol P9883**<br>**Dose level:**	 **TdCyd Dose:**		**Page ______________**<br>**for Sample Pick-up**<br>**Lab phone: ____________**		**Research Nurse:**<br>Phone:
**Patient ID:**<br>*******ONLY PK SAMPLES GO ON ICE********					**Lead AI/PI:**<br>Phone: Pager:
**PLEASE LABEL EACH TUBE WITH ACTUAL DATE AND TIME OF SAMPLE COLLECTION**					
**Day**	**Time**	**Instructions**	**Ideal Time**	**Actual Time**	**Record comments (i.e., if collection missed), and sign each time you collect a sample**
**Day 1**	**Prior to drug administration**	**PK 200** Heparin + ZEB (green top) 3 mL<br>Label tube: sample number, date and time<br>**PLACE ON ICE**	**Morning of drug administration**		
**Day 1**	**Prior to drug administration**	**PD 400** 7.5mL CellSave<br>Label tube: sample number, date and time	**Morning of drug administration**		
**Administer TdCyd, Time:**					
**Day 1**	**1 hour after drug**	**PK 201** Heparin + ZEB (green top) 3 mL<br>Label tube: sample number, date and time<br>**PLACE ON ICE**			
**Day 1**	**2-4 hours after drug**	**PD 401** 7.5mL CellSave<br>Label tube: sample number, date and time			
**Day 1**	**4 hours after drug**	**PK 202** Heparin + ZEB (green top) 3 mL<br>Label tube: sample number, date and time<br>**PLACE ON ICE**			
**Day 1**	**24 hours after drug**	**PK 203** Heparin + ZEB (green top) 3 mL<br>Label tube: sample number, date and time<br>**PLACE ON ICE**			

Date:		PK/PD BLOOD SAMPLE COLLECTION SHEET: CYCLE 1 DAY 8			
CTEP Protocol P9883<br>Dose level: TdCyd Dose:<br>Patient ID:<br>*****ONLY PK SAMPLES GO ON ICE******			Page ______________<br>for Sample Pick-up<br>Lab phone: ___________		Research Nurse:<br>Phone:<br>Lead AI/PI:<br>Phone: Pager:
PLEASE LABEL EACH TUBE WITH ACTUAL DATE AND TIME OF SAMPLE COLLECTION					
**Day**	**Time**	**Instructions**	**Ideal Time**	**Actual Time**	**Record comments (i.e., if collection missed), and sign each time you collect a sample**
Day 1	Prior to drug administration	**PK 204** Heparin + ZEB (green top) 3 mL<br>Label tube: sample number, date and time<br>**PLACE ON ICE**			
Day 8	Any	**PD 402** 7.5mL CellSave<br>Label tube: sample number, date and time			

Date:		PK/PD BLOOD SAMPLE COLLECTION SHEET: CYCLE 1 DAY 15			
CTEP Protocol P9883<br>Dose level: TdCyd Dose:<br>Patient ID:<br>*****ONLY PK SAMPLES GO ON ICE******			Page ______________<br>for Sample Pick-up<br>Lab phone: ___________		Research Nurse:<br>Phone:<br>Lead AI/PI:<br>Phone: Pager:
PLEASE LABEL EACH TUBE WITH ACTUAL DATE AND TIME OF SAMPLE COLLECTION					
**Day**	**Time**	**Instructions**	**Ideal Time**	**Actual Time**	**Record comments (i.e., if collection missed), and sign each time you collect a sample**
Day 15	Any	**PK 205** Heparin + ZEB (green top) 3 mL<br>Label tube: sample number, date and time<br>**PLACE ON ICE**			

Date:	PK/PD BLOOD SAMPLE COLLECTION SHEET: CYCLE 2 DAY 1				
**CTEP Protocol P9883**<br>**Dose level:** **TdCyd Dose:**<br>**Patient ID:**			**Page ____________**<br>**for Sample Pick-up**<br>**Lab phone: __________**		**Research Nurse:**<br>Phone:<br>**Lead AI/PI:**
*****ONLY PK SAMPLES GO ON ICE******					

PLEASE LABEL EACH TUBE WITH ACTUAL DATE AND TIME OF SAMPLE COLLECTION

Day	Time	Instructions	Ideal Time	Actual Time	Record comments (i.e., if collection missed), and sign each time you collect a sample
Day 1	Prior to drug administration	**PK 206** Heparin + ZEB (green top) 3 mL<br>Label tube: sample number, date and time<br>**PLACE ON ICE**	Morning of drug administration		
Day 1	1 hour after drug	**PK 207** Heparin + ZEB (green top) 3 mL<br>Label tube: sample number, date and time<br>**PLACE ON ICE**			
Day 1	4 hours after drug	**PK 208** Heparin + ZEB (green top) 3 mL<br>Label tube: sample number, date and time<br>**PLACE ON ICE**			
Day 1	24 hours after drug	**PK 209** Heparin + ZEB (green top) 3 mL<br>Label tube: sample number, date and time<br>**PLACE ON ICE**			
Day 1	Any	**PD 403** 7.5mL CellSave<br>Label tube: sample number, date and time			

Date:	PK/PD BLOOD SAMPLE COLLECTION SHEET: CYCLE 3 DAY 1				
CTEP Protocol P9883<br>Dose level:<br>Patient ID:	TdCyd Dose:		Page ______________<br>for Sample Pick-up<br>Lab phone: ___________		Research Nurse:<br>Phone:<br>Lead AI/PI:<br>Phone: Pager:
PLEASE LABEL EACH TUBE WITH ACTUAL DATE AND TIME OF SAMPLE COLLECTION					
**Day**	**Time**	**Instructions**	**Ideal Time**	**Actual Time**	**Record comments (i.e., if collection missed), and sign each time you collect a sample**
Day 1	Any	**PD 404** 7.5mL CellSave<br>Label tube: sample number, date and time			

Date:	PK/PD BLOOD SAMPLE COLLECTION SHEET: CYCLE 4 DAY 1				
CTEP Protocol P9883<br>Dose level:<br>Patient ID:	TdCyd Dose:		Page ______________<br>for Sample Pick-up<br>Lab phone: ___________		Research Nurse:<br>Phone:<br>Lead AI/PI:<br>Phone: Pager:
PLEASE LABEL EACH TUBE WITH ACTUAL DATE AND TIME OF SAMPLE COLLECTION					
**Day**	**Time**	**Instructions**	**Ideal Time**	**Actual Time**	**Record comments (i.e., if collection missed), and sign each time you collect a sample**
Day 1	Any	**PD 405** 7.5mL CellSave<br>Label tube: sample number, date and time			

Date:	PK/PD BLOOD SAMPLE COLLECTION SHEET: DAY 1 EACH CYCLE/ DETERMINED BY PI				
**CTEP Protocol P9883**<br>**Dose level:**<br>**Patient ID:**	**TdCyd Dose:**		**Page _______________**<br>**for Sample Pick-up**<br>**Lab phone: ____________**		**Research Nurse:**<br>Phone:<br>**Lead AI/PI:**<br>Phone: Pager:
**PLEASE LABEL EACH TUBE WITH ACTUAL DATE AND TIME OF SAMPLE COLLECTION**					
**Day**	**Time**	**Instructions**	**Ideal Time**	**Actual Time**	**Record comments (i.e., if collection missed), and sign each time you collect a sample**
	**Any**	**PD 40X** 7.5mL CellSave<br>Label tube: sample number, date and time			
